# The impact of peer attachment on prosocial behavior, emotional difficulties and conduct problems in adolescence: The mediating role of empathy

**DOI:** 10.1371/journal.pone.0227627

**Published:** 2020-01-10

**Authors:** Konstanze Schoeps, Estefanía Mónaco, Amparo Cotolí, Inmaculada Montoya-Castilla

**Affiliations:** Department of Personality, Assessment and Psychological Treatment, University of Valencia, Valencia, Spain; Institute of Physiology and Basic Medicine, RUSSIAN FEDERATION

## Abstract

Attachment theories postulate that during adolescence, peer relationships become more important as a predictor of positive social, emotional and behavioral outcomes. Adolescents develop the ability to empathize with others, which is related to healthy functioning and positive peer relationships. Empathy has been studied as a potential mechanism that may help to explain how strong and healthy emotional bonds are associated with less emotional disorders and conduct problems in youth. The main purpose of this study was to examine the relationship between peer attachment and strengths and difficulties during adolescence, considering empathy as a potential mediator of this association. A total of 800 Spanish adolescents (56.65% girls), aged between 12 and 15 years (*M* = 14.02, *SD* = 1.21), completed measures of peer attachment, empathy, conduct problems, emotional difficulties and prosocial behavior. Structural equation models indicated that peer attachment was negatively associated with conduct problems and emotional difficulties but positively related to prosocial behavior. In general, empathy mediated the link between peer attachment and both emotional and behavioral outcomes, without significant group differences between boys and girls. The discussion focuses on the importance of healthy peer relationships as a powerful predictor of emotional well-being and psychological problems in adolescence.

## Introduction

### Emotional and behavioral strengths and difficulties in adolescence

Emotional and behavioral problems in adolescence have been a major concern to both professionals and families [1]. Emotional difficulties refer to symptoms of depression or anxiety, while behavioral problems involve disruptive behaviors such as getting into fights, behaving impulsively, losing self-control, disobeying, lying or stealing [2,3]. Although these difficulties do not reach the severity of psychopathological disorder, they interfere in adolescents’ psychological functioning and how they interact with their environment [4]. Adolescence is a developmental stage of increasing vulnerability for the emergent emotional and behavioral problems, because the individual is in process of building his or her identity [5]. In addition, adolescents are facing a variety of social and psychological stressors due to changes in their physical, mental, emotional, and social development [6].

According to epidemiological studies carried out in Spain [7], emotional and behavioral difficulties are common during adolescence. Between 11.6% and 34.6% of Spanish adolescents present emotional symptoms, while between 4.9% and 25.7% present behavioral problems. It is well-known that emotional and behavioral difficulties have a significant impact on adolescents’ personal, academic, family, and social lives [4]. Thus, adolescents who are emotionally unstable and/or engage in disruptive behavior also present academic difficulties, interpersonal conflicts, risky behaviors and physical health problems more frequently [8,9]. Furthermore, people who present emotional and/or behavioral symptoms during adolescence are more likely to develop a mental disorder subsequently during adulthood [5]. Such prolongation of psychopathology has relevant consequences for society by becoming a public health problem and an economic burden [10].

Previous research has focused on the study of difficulties and problematic circumstances during adolescence, while other complementary investigations emphasize the importance of promoting adolescents’ strengths, such as socio-emotional competences and prosocial behavior [11]. Prosocial behavior is a voluntary behavior which main intention is to benefit others, for instance, by sharing generously, cooperating with minority groups, helping and comforting peers [12]. Adolescents who are more prosocial, tend to be less aggressive and get along better with their peers. Therefore, teenagers who engage in such helping activities are more likely to have better socioemotional adjustment and well-being [13]. Further studies suggest that, prosocial behaviors increase during adolescence [7]. These findings emphasize the need to enhance and promote adolescents’ strengths such as prosocial behavior [13].

### Importance of secure peer attachment relationships

During the transition from infancy to adolescence, children become more independent from their parents and turn to their peers for social support [14]. As peers become a strong socializing agent, attachment to parents remains an equally essential part of their lives [15]. However, it is important to study adolescents’ attachment relationships with their peers, and how such relationships affect their emotions and behaviors [16].

Attachment is a concept that has been traditionally studied regarding parent-child relationships [17]. According to Bowlby's attachment theory [18], it is an emotional bond that is developed in early childhood based on how the primary caregiver (parent) responds to the needs of the baby. Therefore, early bonding experiences established with the parents will influence adolescents' emotional development and their relationship skills with their peer group [19]. Bretherton and Munholland [20] argue the child-parent relationship leads to the development of an *internal working model* (IWM). This mental scheme involves a cognitive framework about the world and other people, including a mental representation of oneself in relation to the environment. Thus, the early patterns of interactions and the resulting mental representations usually persist along the individual's life, guiding his or her attitudes, behaviors, and expectations about others [21].

Individuals who developed a secure attachment to their parents are more likely to establish a secure attachment bond with their peers [22]. The study of peer attachment is of great interest especially in adolescence, when social relationships become more complex [23,24]. A secure peer attachment is defined as a relationship established on a basis of trust, along with the belief that the other person will respect one’ own needs and desires, and she will be able to both understand and respond if when communicating our feelings [25]. Thus, secure peer attachment relationships in adolescence are based on mutual understanding, trust and good communication quality [23,26]. On the contrary, insecure peer attachment is characterized by the feeling of alienation and isolation from the peer-group. This feeling might be due to a fear of rejection at the same time to a desire of proximity and affiliation, along with poor communication and mistrust [27]. Hence, adolescents who perceive their peers as neither supportive nor trustworthy and often feel isolated from their peer group, tend to present emotional and behavioral difficulties [28]. Previous research has supported the role of peer attachment in explaining adolescent’s engagement in behavioral problems such as aggressive behavior and substance abuse [29,30]. Additionally, attachment insecurity increases the possibility of emotional symptoms such as depression and anxiety [31]. These difficulties might be related to the feeling of agony when an adolescent feels unable to trust others and communicate their own emotional dilemma, especially when group affiliation is of such importance in this developmental stage [28].

Furthermore, adolescents who establish secure peer attachment relationships, are more likely to engage in prosocial behaviors [32]. They have developed positive internal working models which motivates adolescents to collaborate with each other because they believe it is worth it [33]. Such secure attachment may contribute to children’s ability to form meaningful peer relationships when they enter school [34]; these relationships may provide increased opportunities to participate in prosocial behaviors, which in turn sustain positive interactions with peers into adolescence, reinforcing children’s views of others as good and worthy of care [35]. On the contrary, insecure attached adolescents have negative expectations and hostile attributions about peer behaviors that likely undermines prosocial behavior [36,37].

### The mediating role of empathy in the interplay between peer attachment and adolescents’ difficulties and strengths

Recent studies have started to examine empathy as one of the potential mechanisms that may help explain how attachment relationships are associated with emotional and behavioral difficulties and strengths [38]. The definition of empathy has been debated over decades [39]. Some authors [40] have proposed a multidimensional approach, while others have separated the construct into cognitive empathy (ability to understand other person’s feelings) and affective or emotional empathy (the experience of emotion, elicited by an emotional event or experience of someone) [41]. Thus, empathy can be defined as an emotional response of one individual based on his or her own perception and understanding, regarding the observed experience of another [39,42].

Regarding the association between peer attachment and the development of empathic capacities, empirical studies provide evidence that empathic responses to self and others are based on positive internal working models (IWMs) [25]. Adolescents with secure attachment, that is, with positive IWMs about themselves and their environment, perceive others with greater esteem and acceptance, trust in them and attribute a positive intention to other behavior rather than hostile attitudes, and they also perceive themselves as capable of taking care of others [20]. Thus, the internal working models that are at the base of our social functioning could be one of the mechanisms that explain the interaction between attachment and empathy [43].

The impact of the ability to empathize with others on emotional and behavioral functioning depends on the developmental phase [44]. In childhood, low empathy is related to poor peer relationships, victimization and bullying behavior [45,46]. In adolescence, low empathy manifests in aggression and antisocial behavior, as well as emotional difficulties [43,47,48]. Conversely, greater empathy is associated with social competence and prosocial behavior across the lifespan, more empathic individuals are more likely to share resources, to provide help to those in need, and to care for others in distress [25]. Thus, adolescents who develop optimal levels of empathy are more likely to show altruistic and prosocial behaviors, which in turn are associated with healthy socio-emotional functioning [32,49].

Previous research suggests that empathy might be mediating the relationship between peer attachment and emotional and behavioral outcomes [23,33,50]. Laible [31] examined the simultaneous effect of parent and peer attachment on aggressive and prosocial behavior in late adolescents, and the results showed that for this age-group parent and peer attachment were indirectly related to aggressive behavior through its link with empathy. Similarly, Carlo et al. [33] observed in a sample of Latino college students that both parent and peer attachment were indirectly associated with social behaviors through empathy, though empathy significantly accounted for the relations of peer (but not parent) attachment and social behaviors. In Korea, You et al. [50] examined how various forms of attachment (maternal, peer, and school) were directly or indirectly related to bullying, and whether cognitive and affective empathy mediates the relationship. Interestingly, different patterns were observed for male and female middle school students. However, the results obtained in other countries should not be generalized due to possible cultural and contextual differences in the psychological variables studied [51], so it is necessary to carry out studies in Spanish adolescent population.

### Present study

While previous research has established overall links between attachment security to parents and children’s behavior and emotional functioning, more research is needed to study the role of attachment to peers in adolescence, given the importance of the peer group in this developmental stage [16,19]. Furthermore, no research has been found in Spanish adolescent population investigating the possible mediating role of cognitive and affective empathy in the relationship between peer attachment and behavioral and emotional strengths and difficulties [52].

Another issue that needs to be further explored is the influence of gender on the proposed relationship. Although previous literature suggests that there are differences between girls and boys in the variables studied, those findings are inconsistent. With regard to peer attachment, some authors suggest that girls establish more secure attachment in their peers relationships than boys, based on trust and more efficient communication skills [53]. Furthermore, there is often a consensus that boys report more externalized problems related to their behavior (such as physical aggressive behavior), while girls report more frequently internalized symptoms (such as emotional distress, anxious and depressive symptoms) [54]. This could be related, among other things, to a particularly high affective empathy [55]. In other words, empathic capacity and the tendency to adopt and understand friend’s point of view is associated with higher attachment quality [22]. However, adolescents who feel more sympathy and concern for others in need might experience more emotional distress due to their heightened sensitivity and investment in peer relationships, especially girls [49].

Hence, the purpose of this study was to examine whether adolescents’ peer attachment (trust, communication and alienation) might influence the develop of emotional and behavioral strengths and difficulties, and whether this relationship is mediated by cognitive or/and affective empathy, considering potential gender differences.

Based on the theoretical and empirical literature, it was expected that (1) adolescents with secure peer relationships (high levels of trust and communication and low levels of alienation) would report higher levels of empathy (cognitive and affective) and prosocial behavior, and lower levels of emotional difficulties and conduct problems; (2) adolescents with higher cognitive and affective empathy were expected to relate higher prosocial behavior and less conduct and emotional problems; and (3) empathy (cognitive and affective) would mediate the associations between peer attachment and social-emotional difficulties (both behavioral and emotional problems) and strengths (prosocial behavior). In addition, differences between boys and girls in the proposed model will be explored.

## Materials and methods

### Participants

Participants were 800 students (56.65% girls), aged between 12 and 15 years (*M*age = 14.02, *SD* = 1.21), from 1^st^ to 4^th^ grade of compulsory secondary education at five public and three private secondary schools from Valencian Community, Spain. Subsamples were distinguished in order to examine potential gender differences: girls (*N* = 456, *M* = 13.62, *SD* = 1.17) versus boys (*N* = 349, *M* = 13.79, *SD* = 1.26), considering differences in attachment patterns and in vulnerability regarding emotional and behavioral strengths and difficulties according to gender.

### Procedure

For this study, a cross-sectional design with self-report data from a convenience sample was used. All procedures were conducted following the ethical standards of the Helsinki Declaration [56]. Prior to data collection, permission to conduct the study was obtained from the Department of Education, Culture and Sport of Valencia, as well as written informed consent from parents of participating students. Information sessions were organized for school staff, parents and students, where they were briefed about the voluntary nature of their participation, including confidentiality and their right to refuse any answers or withdraw from the study at any time. Data were collected at the beginning of the school year, during regular class time in classrooms and took approximately 50 minutes. In each session, at least two trained psychologists were present to clarify any doubts about the items or questions.

### Instruments

The measures used in this study are self-report instruments, adapted and validated for the Spanish population. The reliability values Cronbach’s α, Average Variance Extracted (AVE) and Composite Reliability Coefficient (CRC) are based on the sample from this study.

#### Peer attachment

To assess student’s peer attachment, the Inventory of Parent and Peer Attachment (IPPA) was used; a self-report questionnaire developed by Armsden and Greenberg [57], adapted into Spanish by Gallarin and Alonso-Arbiol [58]. The measurement is a 75-item scale, which is divided into three 25-item subscales: one for measuring the attachment towards the father, one for assessing attachment towards the mother, and the third for assessing peer attachment. In this study, we were only interested in this last subscale that comprised three subscales with a five-point Likert scale (*1 = Never True; 5 = Always True*): trust, which refers to the perception of mutual trust and respect for each other’s needs and desires (e.g., “My friends accept me as I am.”), communication means the perceived quality of involvement, responsiveness and verbal communication regarding adolescent’s emotional states (e.g., “My friends can tell when I’m upset about something.”) and alienation involves the feeling of social isolation, anger and detachment from peers, but with the recognition of the need to be closer to them (e.g., “I feel alone or apart when I am with my friends.”). In a validation study carried out with Spanish adolescents, satisfactory internal consistency coefficients (α ≥ 0.68) for each scale were found [59]. The reliability obtained with the data from this study was excellent, except for alienation: trust (α = .84; AVE = .52; CRC = .80), communication (α = .84; AVE = .51; CRC = .93), alienation (α = .57; AVE = .30; CRC = .55).

#### Social and emotional strengths and difficulties

To measure adolescents’ prosocial behavior, conduct problems and emotional difficulties, we assessed the Spanish adaptation [54] of the Strengths and Difficulties Questionnaire (SDQ) developed by Goodman [60]. From the 25-item questionnaire only three out of five scales were used for the purposes of the present study: prosocial behavior, (e.g., “I try to be nice to other people. I care about their feelings.”), emotional difficulties (e.g., “I get many headaches, stomach-aches or sicknesses.”), conduct problems (e.g., “I get very angry and often lose my temper.”). Respondents are required to indicate how much the attribute applies to them on a three-point Likert scale (*1 = Not True; 3 = Certainly True*). With data from this sample the reliability was rather critical but sufficient for all subscales: prosocial behavior (α = .59; AVE = .38; CRC = .77), emotional difficulties (α = .68; AVE = .37; CRC = .78), conduct problems (α = .53; AVE = .46; CRC = .83).

#### Empathy

Student’s empathy was measured with the Basic Empathy Scale (BES) [61], using the Spanish adaption [62]. The instrument is a 20-item scale that assesses emotional and cognitive empathy (e.g., “After being with a friend who is sad about something, I usually feel sad.” and “I can understand my friend’s happiness when she/he does well at something.”). Participants were required to answer on a 5-point Likert scale (*1 = do not agree; 5 = totally agree)*. The scale shows good psychometric properties in Spanish young people [48]. The reliability corresponding to the data was acceptable: Emotional Empathy (α = .61; AVE = .36; CRC = .84), Cognitive Empathy (α = .75; AVE = .41; CRC = .85).

### Data analyses

A five-step procedure with two-phase SEM analysis was adopted to test our hypothesis [63]: (a) analysis of the reliability of the instruments, using the Cronbach alpha coefficient (α), the Composite Reliability Coefficient (CRC), and the Average Variance Extracted (AVE), which have been reported above in the method section; (b) bivariate correlations between the studied variables were performed; (c) Confirmatory Factor Analysis (CFA) to test the adequacy of the measurements and structural portions of the model separately and address misspecifications before assessing the structure among latent variables (measurement phase); (d) Structural Equation Modeling (SEM) analysis with latent factors was conducted to predict the pathways from peer attachment based on trust, communication and alienation to prosocial behavior, emotional difficulties and conduct problems, acting as a mediator emotional and cognitive empathy (structural phase); (e) multi-group analyses to identify gender differences.

Regarding reliability measures, Cronbach's α > .70 is a widely used cut-off point, but the critical value of α > .50 is sufficient for building structural equation models [64]. In addition, alternative estimators for composite reliability have been established with AVE levels > .50 and CRC levels > .70 that are considered adequate [65,66].

Confirmatory models were estimated using Weighted Least Squares (WLSMV), has been shown to perform very well in the case of ordinal (less than seven categories) and non-normal data [66,67]. The fit of the model was estimated using four indices recommended by Hu and Bentler [68]: Chi-Square Test of Model Fit (χ^2^), Comparative Fit Index (CFI), Tucker Lewis Index (TLI), and Root Mean Square Error of Approximation (RMSEA). In addition, the Weighted Root Mean Square Residual (WRMR) has been calculated, a weighted version of the RMSEA proposed when WLSMV is used. Generally, it is assumed that a CFI/TLI higher or equal to .90 together with a measure of amount of error such as the RMSEA lower than .08 are considered satisfactory, however with CFI/TLI > .95 and RMSEA < .05 indicating excellent model fit [69,70]. The percentage of missing data is < 3%, but in any case the procedure for handling the missing data is done through Full Information Maximum Likelihood (FIML). A variety of statistical methods are available to test for mediation, but experts recommend structural equation modeling due to the many advantages, such as building on confirmatory factor analysis by using several observed indicators for an unobserved construct (latent variables), explicit testing of measurement invariance, focus on evaluating model fit and accounting for measurement error [71,72]. Furthermore, total effects, direct effects and indirect effects were estimated, constructing bootstrap confidence intervals (CI) around the estimates to assess the effects of mediators [73–75]. This method offers a more reliable estimation than the traditional Sobel test [75] or the causal step method by Baron and Kenny [74] for testing indirect effects [76].

These analyses were conducted with SPSS V.24 and MPLUS 7.0 [77] and the results were reported following the recommendations of the APA Working Group on Quantitative Research Reporting Standards [78].

## Results

### Bivariate correlations between variables

The descriptive statistics, such as correlational analyses (Table 1), means and standard deviations (Table 2) have been provided for all measures. Results from bivariate correlations revealed that the higher students scored on the peer trust scale, the higher they perceived levels of cognitive and emotional empathy as well as prosocial behavior, but the less they informed about conduct problems and emotional difficulties. Similarly, the results indicated that students with higher scores on the peer communication scale also reported higher levels of cognitive and emotional empathy and prosocial behavior, but less conduct problems and emotional difficulties. Furthermore, the higher students scored on peer alienation, the more emotional empathy, conduct problems and emotional difficulties they reported. Moreover, there was no significant correlation between peer alienation and cognitive empathy or peer alienation and prosocial behavior. In summary, students who establish peer relationships based on mutual trust and high communication quality, tend to be more empathic (cognitive and emotionally) and show more prosocial behavior. In contrast, adolescents who feel detached and isolated from their peer group, although they have a stronger emotional empathic capacity, seem to struggle more with behavioral and emotional problems.

**Table 1:** Bivariate correlations between variables of interest.

	1	2	3	4	5	6	7
1. Peer Trust	−						
2. Peer Communication	.70^a^	−					
3. Peer Alienation	-.44^a^	-.23^a^	−				
4. Cognitive Empathy	.33^a^	.38^a^	-.03	−			
5. Emotional Empathy	.23^a^	.35^a^	.05^b^	.52^a^	−		
6. Emotional difficulties	-.24^a^	-.06^b^	.48^a^	.04	.18^a^	−	
7. Conduct problems	-.16^a^	-.08^b^	.19^a^	-.10 ^b^	-.10^b^	.21^a^	−
8. Prosocial behavior	.26^a^	.29^a^	-.01	.28^a^	.27^a^	.03	-.13^a^

^a^*p* < .05.

^b^*p* < .01.

**Table 2:** Descriptive statistics for all measures.

Measures	*M*^*a*^ *(SD*^*b*^*)*	Range	*Skewness*	*Kurtosis*	*20%*^*c*^	*40%*^*c*^	*60%*^*c*^	*80%*^*c*^
1. Peer Trust	41.84 (6.29)	11–50	-1.28	2.36	37.00	41.00	44.00	47.00
2. Peer Communication	30.16 (5.81)	11–40	-0.57	-0.02	25.00	29.00	32.00	35.00
3. Peer Alienation	16.36 (4.06)	6–30	0.40	0.26	13.00	15.00	17.00	20.00
4. Cognitive Empathy	34.52 (5.88)	4–45	-0.67	1.74	30.00	34.00	36.00	39.00
5. Emotional Empathy	38.66 (6.22)	5–55	-0.58	1.50	33.00	37.00	41.00	45.00
6. Emotional difficulties	8.16 (2.86)	5–15	0.68	-0.01	6.00	7.00	8.00	10.00
7. Conduct problems	6.04 (2.46)	3–14	1.13	1.70	5.00	5.00	6.00	8.00
8. Prosocial behavior	13.24 (3.64)	6–15	´0.94	0.88	12.00	13.00	14.00	15.00

*M*^*a*^ = Mean.

*SD*^*b*^ = Standard Deviation.

%^c^ = Percentiles.

### Confirmatory Factor Analysis (CFA)

Confirmatory Factor Analyses (CFA) were conducted to test the adequacy of the measurements in the sample of this study (measurement phase). In particular, Exploratory Factor Analysis (EFA) confirmed the factorial structure of the IPPA comprising three factors defended by the authors. A first Confirmatory Factor Analysis (CFA) of the three-dimensional structure with this sample offers unsatisfactory fit indices: χ^2^ (272) = 1298.244, p < .001, CFI = .92, TLI = .91, RMSEA = .07. We proceeded to perform a second CFA eliminating five items with low saturation on the latent factor (< .40), which provides the following fit indices: χ^2^ (167) = 702.333, p < .001, CFI = .95, TLI = .95, RMSEA = .06, which are more satisfactory than the previous one.

When the SDQ scale was submitted to a CFA in this sample, the results show a good fit to the data: χ^2^ (87) = 187.264, p < .001, CFI = .94, TLI = .93, RMSEA = .04.

In order to replicate the original factors structure of the BES with this sample, EFA and CFA were conducted, which confirmed the two-dimensional structure proposed by the authors. However, the fit indices of the original 20-item scale are inadequate: χ^2^ (169) = 983.616, p < .001, CFI = .87, TLI = .85, RMSEA = .08. After eliminating six items with low saturation on the latent factor (< .40) a second CFA provides more satisfactory fit indices: χ^2^ (53) = 194.940, p < .001, CFI = .95, TLI = .94, RMSEA = .06.

### Structural Equation Modeling (SEM)

**Fig 1:**
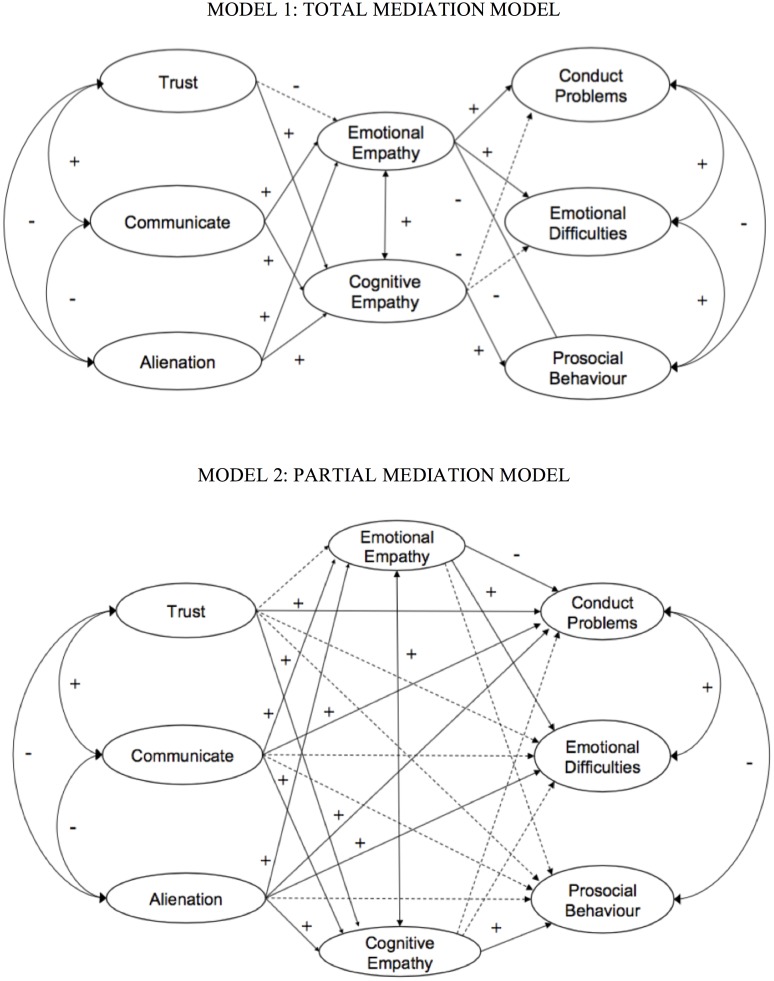
Initial models of full mediation (1) and partial mediation (2). Note: Continuous lines represent a significant relationship with *p* < .01, dotted lines represent non-significant relationships. The relationships of the factors with their indicators have not been drawn for simplicity.

Structural equation modeling analyses were performed to test the hypothesized relationship among latent variables (structural phase). We estimated two subsequent models in order to test the mediation hypotheses efficiently [79]. The hypothesized models were theoretically grounded [31,38,43,50], followed by the correlation analysis and CFA. The first model proposes total mediation since all the effects of peer attachment on adolescents’ strengths and difficulties are indirect through cognitive and emotional empathy (Fig 1). In the second model, mediation is assumed to be partial (Fig 1). That is, additional direct effects are specified between the exogenous factors (peer trust, peer communication and peer alienation) and the final outcome factors (prosocial behaviors, emotional difficulties and conduct problems).

The first model testing full mediation showed an adequate model fit (Fig 1): χ^2^ (1015) = 2245.365, *p* < .001, CFI = .93, TLI = .92, RMSEA = .04 [.03-.04], WRMR = 1.45). The second model (Fig 1), which includes direct effects of peer attachment on socio-emotional strengths and difficulties, fits the data better by showing excellent fit indices: *χ*^*2*^ (1004) = 1931.189, *p* < .001, CFI = .95, TLI = .94, RMSEA = .03 [.03-.04], WRMR = 1.31). Mplus code for Chi-Square Difference Test was used to compare nested models with WLSMV estimation and non-significant *p*-value indicated that the nested model is not significantly worse than the first less restrictive one. Thus, the partial mediation model is the most parsimonious model and should be retained.

#### Direct and indirect effects

**Fig 2:**
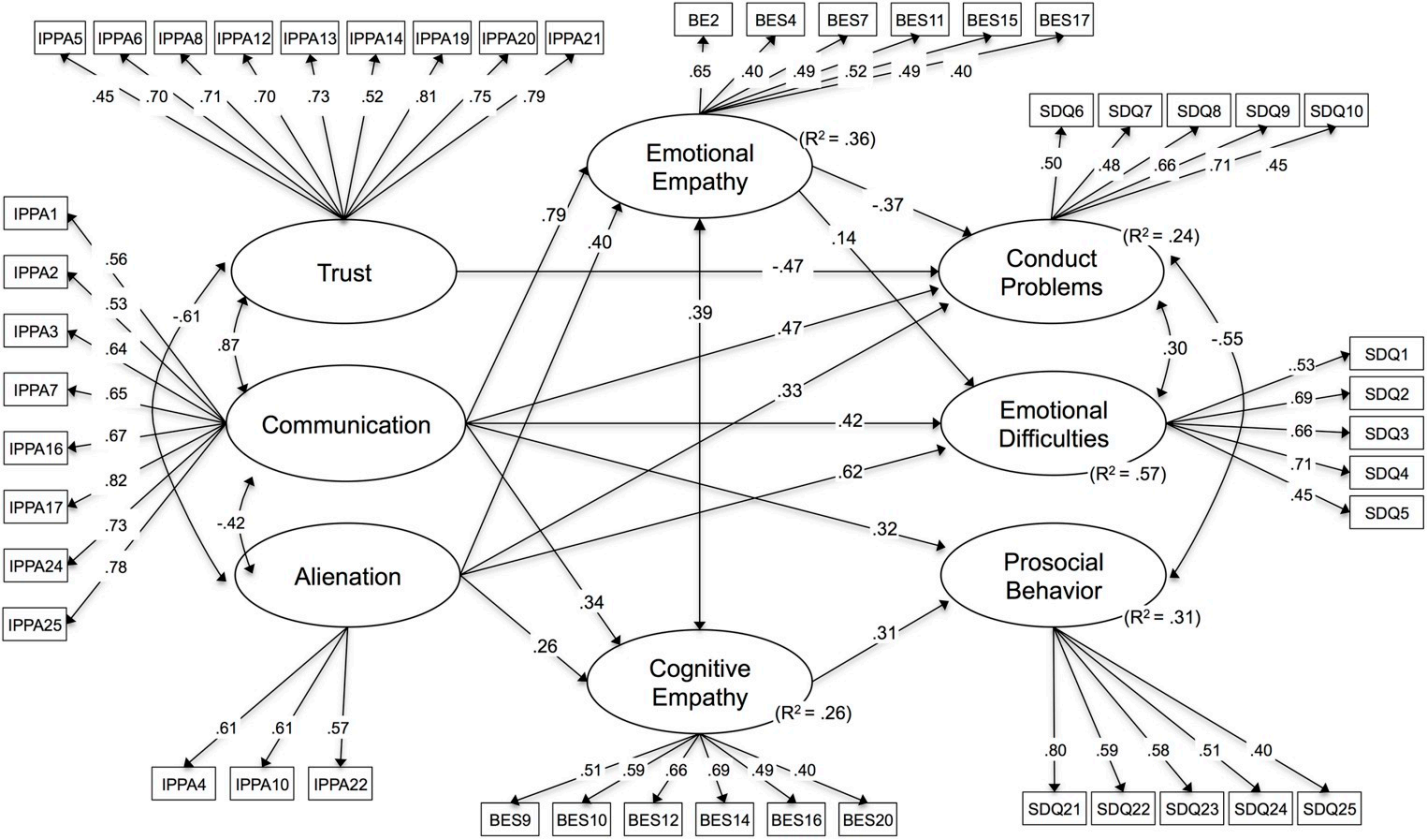
Standardized solution for the tested structural model. Note: All relationships shown are significant with *p* < .05. Factor loading are standardized.

On the partial mediation model of best fit, non significant paths have been eliminated, in order to generate a more parsimonious model. As shown in Fig 2, the saturations of the remaining indicators (items that were maintained after CFA) for all latent factors are adequate (>.40). The results of this model (Table 3) indicate that the direct effect of peer trust, communication and alienation and the indirect effect through emotional empathy explain 24% of the variance of conduct problems. Similarly, emotional difficulties were predicted by the direct effects of adolescents’ peer trust, communication and alienation, and the indirect effect through emotional empathy, explaining 57% of the variance. In contrast, adolescents’ cognitive empathy showed no significant effect on behavioral and emotional problems. With regard to the prediction of prosocial behaviors, 31% of its variance can be explained by the direct effect of peer communication and the indirect effect through cognitive empathy. However, neither peer trust nor alienation nor emotional empathy has significant effects on prosocial behavior.

**Table 3:** Estimated coefficients of total, direct, and indirect effects.

	Total effect			Direct effect			Indirect effect		
	c^a^	SE^b^	95% CI^c^	c’^a^	SE^b^	95% CI^c^	ab^a^	SE^b^	95% CI^c^
**Peer Trust** → Emotional Empathy → Conduct problems	-.38^f^	.20	[-.77, -.01]	-.47^f^	.21	[-.88, -.07]	.10	.07	[-.05, .23]
**Peer Communication** → Emotio**nal Empathy** → Conduct problems	.18	.16	[-.14, .50]	.47^f^	.20	[.08, .86]	-.29^e^	.10	[-.48, -.10]
**Peer Alienation** → Emotional Empathy → Conduct problems	.18	.10	[-.01, .37]	.33^e^	.11	[.11, .55]	-.15^e^	.05	[-.27, -.05]
**Peer Trust** → Emotional Em**pathy** → Emotional Difficulties	-.41^f^	.15	[-.70, -.11]	-.37^f^	.15	[-.67, -.08]	-.03	.03	[-.09, .02]
**Peer Communication** → Emotional Empathy → Emotional Difficulties	.52^d^	.13	[.28, .77]	.42^e^	.15	[.12, .70]	.11	.06	[-.10, .22]
**Peer Alienation** → Emotional **Empathy** → Emotional Difficulties	.67^d^	.07	[.52, .81]	.62 ^d^	.08	[.46, .78]	.05 ^f^	.03	[.01, .11]
**Peer Trust** → Cognitive Empathy → Prosocial Behaviour	.13	.20	[-.26, 51]	.04	.19	[-.34, .42]	.08	.05	[-.01, .18]
**Peer Communication** → Cognitive **Empathy** → Prosocial Behaviour	.43 ^e^	.15	[.12, .73]	.32 ^e^	.15	[.02, .62]	.11 ^e^	.04	[.03, .18]
**Peer Alienation** → Cognitive Empathy → Prosocial Behaviour	.15	.10	[-.05, .34]	.06	.10	[-.13, .26]	.08 ^e^	.03	[.03, .14]

^*a*^ *c*, *c’*, *ab* = Estimators of total, direct and indirect effects.

^*b*^ *SE* = Standard Error.

^*c*^ *95% CI* = 95% bootstraps Confidence Intervals.

^d^ *p* < .001.

^e^ *p* < .01.

^f^ *p* < .05.

Further results of this mediation model are the predictions of the mediator variables (emotional and cognitive empathy) by the exogenous factors (peer trust, communication and alienation). For instance, the variance of emotional empathy is explained in a 36% through direct effects of adolescents’ peer communication and alienation. Similarly, 26% of the variance of cognitive empathy is explained by the direct effects of adolescents’ peer communication and peer alienation. Peer trust is not a significant predictor of adolescents’ empathic capacities, neither emotional nor cognitive.

### Multi-group analyses

We conducted a stepwise multigroup analysis to identify whether the structural equation model differ significantly between girls and boys. In the first step, we applied the semi-restricted model, assuming equal factor loadings, free thresholds, and free regression coefficients. In a second step, we applied the fully-restricted model, assuming equal factor loadings, equal thresholds, and equal regression coefficients across gender. The semi-restricted and the fully-restricted model were compared by means of the χ^2^ difference test [80]. At first, the chi-square differences test between the two models was significant, Satorra-Bentler χ^2^ (29) = 216.46, *p* < .001. A second fully-restricted model, assuming different regression coefficients for the direct paths from peer alienation to conduct problems and emotional difficulties, was compared to the semi-restricted model. The test did not reach significance (χ^2^ (25) = 28.686, *p* = .28) [81], which means that the second fully restricted multi-group model fits the data better than the semi-restricted model. In other words, the interrelations among the study variables did not exhibit different patterns for male and female students, except for the association between alienation and behavioral and emotional difficulties.

## Discussion

The effect of peer attachment on adolescent’s strengths and difficulties—including prosocial behavior, emotional and conduct problems—has been previously studied [16,19,29,31,32]. However, only a limited number of studies have taken into account the mediating role of empathy [33,50], but none of them was carried out in the Spanish adolescent population [51]. The purpose of the present work was to study the pathways through which peer attachment (trust, communication and alienation) affect adolescents’ difficulties (emotional and behavioral problems) and strengths (prosocial behavior) and weather cognitive and emotional empathy mediates this relationship. In addition, this study was interested in exploring gender differences.

The first hypothesis suggested that adolescents with secure peer relationships (high levels of trust and communication and low levels of alienation) would report higher levels of empathy (cognitive and affective). This hypothesis was partially confirmed in our sample, showing that high levels of trust and communication among peers are associated with higher levels of cognitive and affective empathy. These expected results are in line with previous research [25]. However, alienation was positively associated −although weakly− with emotional empathy, and it was unrelated to cognitive empathy. These unexpected results may suggest that adolescents from this study do not lose their ability to experience emotions empathetically as a reaction to what they observe in their environment or in others, even if they feel somehow isolated and detached from their peers. A possible explanation is that one’s own suffering may foster the ability to recognize the others’ suffering [39]. Nevertheless, these relationships should be analyzed in future studies, as the reliability of the alienation scale in our study is not sufficient to make strong claims.

This first hypothesis also suggested that adolescents with secure peer relationships (high levels of trust and communication and low levels of alienation) would report lower levels of emotional difficulties and conduct problems and higher levels of prosocial behavior. This premise is almost fully confirmed by the results from this study. Our results showed that peer trust and communication are negatively associated with emotional difficulties and behavioral problems, and positively with prosocial behavior. That is, those adolescents who share interests and concerns with their peer and feel that they accept their opinions and feelings also report fewer anxious and depressive emotional symptoms, less maladaptive behavior, and greater tendency to help and care for their peers [27,28]. Furthermore, in line with previous studies [37], we observed a positive association between peer alienation and conduct problems, and particularly strong between peer alienation and emotional problems. In other words, adolescents who feel distanced or in conflict with their peer group are more likely to present emotions of anxiety, sadness, worry, aggression and/or apathy (among others), which in turn is related to the development of behavioral problems.

Our second hypothesis, which expected a positive relation between emotional and cognitive empathy with prosocial behavior, and a negative relation with emotional and conduct problems, was partially confirmed. Regarding adolescents’ empathic ability, our results indicated that both emotional and cognitive empathy are positively associated with positive behavior, specifically high prosocial behavior and the absence of behavioral problems, replicating results from previous research [11]. Contrary to the expected, emotional empathy was positively related to emotional symptoms. These results partly support literature that focuses on the emotional sacrifices of empathy, especially in girls [53]. Thus, adolescents who feel more sympathy and concern for others in need might experience more emotional distress due to their heightened sensitivity and investment in peer relationships [42].

With regard to the third hypothesis, both cognitive and emotional empathy were expected to mediate the associations between peer attachment and social-emotional difficulties (both behavioral and emotional problems) and strengths (prosocial behavior). Our results support this hypothesis partially. On the one hand, the pathway to emotional and behavioral problems was mediated by emotional empathy only. On the other hand the prediction for prosocial behavior was mediated by cognitive empathy only. Thus, the results from SEM analysis confirmed that emotional empathy mediates the relationship between peer attachment with emotional and conduct problems, but not cognitive empathy. Moreover, peer relationships based on trust do not seem to be relevant in this interplay. Based on these findings, emotional empathy would be a potential protective factor to work on in adolescents with behavioral problems. Nevertheless, enhancing emotional capacity in young people with negative peer emotions could complicate their emotional problems. Cognitive empathy mediates the association between peer attachment and adolescents’ prosocial behavior, but not emotional empathy. Similarly, trustful peer relationships do not appear to be relevant in this association. These findings suggest that the ability to understand cognitively other people’s needs encourages them to develop caring behaviors of help and companionship. Seemingly, adolescents are capable of behaving prosocially without experiencing the emotions of others in one's own skin.

Finally, our results show that gender, did not affect the interplay of peer attachment and social and emotional strengths and difficulties mediated by cognitive and affective empathy. In other words, the current findings suggest that girls and boys are equally in the way that peer attachment influences their social and emotional functioning through empathy. There are multiple and very diverse results that can be extracted from this research. We consider that the primary message that might be drawn from our findings is the mediating role of empathy, which underlies the impact of peer attachment on adolescents’ prosocial behavior, emotional difficulties and conduct problems. While the emotional component of empathy mediates the pathways to emotional and behavioral problems, it is the cognitive aspect, which mediates the prediction of prosocial behavior.

Though our findings are promising, the present study is not without several limitations. One of the limitations is related to methodology, since self-report measurements have been used to assess all psychological variables. Therefore, the reported perception of the adolescent’s conduct problems or prosocial behavior, might not necessarily coincide entirely with the actual behavior in real life because participants may not be aware of the distortion in their perception or unwillingness to report it [62]. Hence, our findings should be interpreted in light of this self-perception bias when estimating one’s own strength and difficulties. In addition, the sample of this study was obtained by convenience sampling and all variables were collected at a single time point, which should be taken into account when interpreting the results. Another limitation of this research is the use of the IPPA’s subscales (trust, communication and alienation), instead of the general score of peer attachment. Although our findings make a valuable contribution by considering the different factors separately, the alienation subscale does not show enough reliability to make substantial statements, which could be altering the rest of the model, leading to confusing results and some conclusions apparently contradictory to what was expected. Further research is necessary to understand the complexity of the alienation scale, and to study in depth how it is related to the other scales of the questionnaire. It would also be relevant to expand and diversify our sample, for instance, by including adolescents from different geographical areas of the Spanish territory, with varied socioeconomic characteristics, in order to represent the diversity of the Spanish adolescent population. Furthermore, future research could include the use of other measures that complement self-reporting, such as measures of observation of adolescent behaviour by family and teachers. Also, longitudinal research is needed in order to confirm causal relationships between the variables studied and establish solid predictive models. Finally, the statistical analyses performed in this study are one possibility among many, and other equivalent models can be analyzed to contribute to the understanding of adolescent’s strengths and difficulties and their predictors.

There is enough evidence that psycho-educational programs that work on the emotional abilities of adolescents are an effective way to improve their social functioning and improve interpersonal relationships both inside and outside of school settings [82]. From these findings we conclude that empathy training might have great potential for families and educators to encourage adolescents’ prosocial behavior [83]. Also, it would reduce the high number of emotional and behavioral problems in Spanish adolescents, and to prevent the associated problems, promoting their health and well-being [5,8,54]. This suggestion should be taken into account when designing and implementing both intervention and prevention programs, especially in adolescents who lack secure attachment relationships, since they are at higher risk of suffering affective and behavioral disorders [19].

## Supporting information

S1 File Datafile with raw data. (PDF)
