# Supplementary material for: The impact of peer attachment on prosocial behavior, emotional difficulties and conduct problems in adolescence: The mediating role of empathy
Source: PLoS One. 2020 Jan 10;15(1):e0227627. doi: 10.1371/journal.pone.0227627 (PMC6953841; doi:10.1371/journal.pone.0227627)
Supplement: S1 File — (PDF) [file pone.0227627.s001.pdf]

Gender	Age	School	Year	IPPA_A1	IPPA_A2	IPPA_A3	IPPA_A4	IPPA_A5
1,00	12,00	6,00	1	5	5	5	1	1
1,00	12,00	6,00	1	5	3	4	1	1
1,00	12,00	11,00	1	5	4	4	2	1
1,00	12,00	8,00	1	5	5	5	1	1
1,00	12,00	2,00	1	4	5	3	5	1
1,00	12,00	11,00	1	4	4	3	2	1
1,00	12,00	11,00	1	3	4	4	2	5
1,00	12,00	7,00	1	5	4	5	3	1
1,00	12,00	6,00	1	5	4	3	2	1
1,00	12,00	5,00	1	5	5	5	1	1
1,00	13,00	5,00	1	5	4	3	1	2
1,00	12,00	4,00	1	4	5	5	1	1
1,00	12,00	4,00	1	4	3	3	3	3
1,00	12,00	3,00	1	4	3	4	1	1
1,00	12,00	2,00	1	5	4	3	2	2
1,00	12,00	11,00	1	3	5	3	2	1
1,00	12,00	11,00	1	5	5	2	3	3
1,00	12,00	11,00	1	5	4	3	2	1
1,00	12,00	11,00	1	4	2	1	3	1
1,00	12,00	11,00	1	4	3	3	2	3
1,00	12,00	8,00	1	5	5	3	2	1
1,00	13,00	8,00	1	4	5	4	4	2
1,00	13,00	8,00	1	4	4	4	1	1
1,00	14,00	8,00	1	4	5	3	4	3
1,00	14,00	8,00	1	5	5	5	3	1
1,00	12,00	7,00	1	4	3	3	2	1
1,00	12,00	7,00	1	5	4	4	3	1
1,00	12,00	7,00	1	5	3	999	5	1
1,00	12,00	7,00	1	2	3	4	999	1
1,00	12,00	7,00	1	5	5	5	1	1
1,00	12,00	5,00	1	5	5	5	1	1
1,00	12,00	4,00	1	999	3	3	999	3
1,00	12,00	4,00	1	5	5	3	1	1
1,00	12,00	4,00	1	5	4	5	1	1
1,00	12,00	3,00	1	3	4	4	2	1
1,00	12,00	3,00	1	3	3	3	5	4
1,00	12,00	3,00	1	4	4	3	2	5
1,00	12,00	3,00	1	4	5	4	1	1
1,00	13,00	3,00	1	4	4	4	3	2
1,00	13,00	3,00	1	4	3	4	2	1
1,00	12,00	11,00	1	5	5	4	1	1
1,00	12,00	11,00	1	5	5	4	1	1
1,00	12,00	11,00	1	5	3	2	3	4
1,00	12,00	11,00	1	4	1	4	1	5
1,00	12,00	11,00	1	3	4	2	2	3
1,00	13,00	11,00	1	4	4	3	3	4
1,00	12,00	8,00	1	5	5	3	1	1
1,00	12,00	8,00	1	5	4	3	2	1
1,00	13,00	8,00	1	2	4	4	1	1

1,00	12,00	7,00	1	5	3	3	4	1
1,00	12,00	7,00	1	5	5	4	4	2
1,00	12,00	7,00	1	4	5	4	1	999
1,00	12,00	7,00	1	3	2	4	3	1
1,00	12,00	7,00	1	5	1	3	1	1
1,00	12,00	7,00	1	5	4	3	4	2
1,00	13,00	7,00	1	5	4	1	2	5
1,00	13,00	6,00	1	5	5	3	3	3
1,00	13,00	6,00	1	3	3	3	2	1
1,00	12,00	5,00	1	2	5	3	2	3
1,00	12,00	5,00	1	5	5	3	1	1
1,00	12,00	5,00	1	4	5	3	1	1
1,00	12,00	4,00	1	1	5	2	5	4
1,00	12,00	3,00	1	5	4	4	2	2
1,00	12,00	3,00	1	5	4	4	1	2
1,00	13,00	3,00	1	4	2	2	2	1
1,00	12,00	2,00	1	5	3	4	2	1
1,00	13,00	2,00	1	5	4	4	2	3
1,00	12,00	11,00	1	4	3	2	2	1
1,00	12,00	11,00	1	2	4	3	2	5
1,00	12,00	11,00	1	4	5	4	2	3
1,00	12,00	11,00	1	4	5	5	1	5
1,00	12,00	11,00	1	5	4	4	2	3
1,00	12,00	11,00	1	4	3	3	4	1
1,00	12,00	11,00	1	3	5	4	1	1
1,00	12,00	11,00	1	5	4	4	2	2
1,00	12,00	11,00	1	5	5	4	1	1
1,00	12,00	11,00	1	5	5	3	2	2
1,00	12,00	11,00	1	5	5	4	3	4
1,00	14,00	11,00	1	5	4	3	3	5
1,00	12,00	8,00	1	5	4	4	3	1
1,00	12,00	8,00	1	5	4	3	3	1
1,00	12,00	8,00	1	5	5	5	1	1
1,00	12,00	8,00	1	5	1	1	5	5
1,00	12,00	8,00	1	5	2	3	5	3
1,00	14,00	8,00	1	4	5	5	1	1
1,00	12,00	8,00	1	4	4	5	1	1
1,00	12,00	8,00	1	3	3	4	1	1
1,00	12,00	8,00	1	5	5	4	1	1
1,00	13,00	8,00	1	5	5	4	3	1
1,00	13,00	8,00	1	3	5	4	5	3
1,00	12,00	7,00	1	4	4	2	3	3
1,00	12,00	7,00	1	5	4	5	4	1
1,00	12,00	7,00	1	5	3	4	1	999
1,00	12,00	7,00	1	5	3	4	2	2
1,00	12,00	7,00	1	4	3	3	2	1
1,00	13,00	7,00	1	5	4	4	1	1
1,00	12,00	6,00	1	5	5	5	2	1
1,00	12,00	6,00	1	5	3	2	1	1
1,00	12,00	6,00	1	4	3	4	2	2

1,00	12,00	6,00	1	4	5	4	1	3
1,00	12,00	6,00	1	5	4	3	3	1
1,00	13,00	6,00	1	5	5	5	1	4
1,00	12,00	5,00	1	5	3	3	5	2
1,00	12,00	5,00	1	5	4	5	1	3
1,00	12,00	5,00	1	5	5	5	1	999
1,00	12,00	5,00	1	4	5	3	2	2
1,00	12,00	5,00	1	5	5	5	1	1
1,00	12,00	5,00	1	3	5	3	5	4
1,00	12,00	5,00	1	5	5	5	1	1
1,00	12,00	5,00	1	3	4	2	2	3
1,00	13,00	5,00	1	4	4	4	2	2
1,00	12,00	4,00	1	5	5	3	1	1
1,00	12,00	3,00	1	1	5	3	1	2
1,00	12,00	3,00	1	4	5	3	1	1
1,00	12,00	3,00	1	4	3	3	2	2
1,00	12,00	3,00	1	3	5	2	2	2
1,00	12,00	3,00	1	3	5	4	1	1
1,00	12,00	3,00	1	5	3	5	1	2
1,00	13,00	3,00	1	3	5	3	1	5
1,00	14,00	3,00	1	5	5	4	2	4
1,00	12,00	2,00	1	3	4	3	2	1
1,00	12,00	2,00	1	5	5	4	1	1
1,00	13,00	2,00	1	3	3	3	2	2
1,00	14,00	11,00	2	3	4	2	1	5
1,00	14,00	8,00	2	5	4	3	2	1
1,00	13,00	3,00	2	3	4	4	2	1
1,00	13,00	11,00	2	5	5	4	1	1
1,00	13,00	11,00	2	5	4	2	2	2
1,00	13,00	11,00	2	3	5	3	1	4
1,00	13,00	8,00	2	4	5	3	2	1
1,00	15,00	8,00	2	5	4	5	1	1
1,00	13,00	7,00	2	4	4	4	5	2
1,00	12,00	5,00	2	5	4	4	3	1
1,00	13,00	5,00	2	5	4	3	4	2
1,00	13,00	4,00	2	3	2	4	2	2
1,00	13,00	4,00	2	4	4	4	2	1
1,00	13,00	11,00	2	3	5	4	4	4
1,00	13,00	11,00	2	4	3	3	2	2
1,00	14,00	11,00	2	5	3	4	1	5
1,00	13,00	8,00	2	4	4	4	3	1
1,00	13,00	8,00	2	3	2	4	1	2
1,00	13,00	8,00	2	5	4	4	3	1
1,00	13,00	8,00	2	4	3	3	2	1
1,00	14,00	8,00	2	4	3	4	3	1
1,00	14,00	8,00	2	5	5	5	1	1
1,00	14,00	8,00	2	5	999	2	3	4
1,00	15,00	8,00	2	5	5	4	1	1
1,00	15,00	8,00	2	5	5	4	4	2
1,00	13,00	7,00	2	4	4	3	1	1

1,00	13,00	7,00	2	4	5	3	1	3
1,00	13,00	7,00	2	999	4	3	1	1
1,00	13,00	7,00	2	4	3	4	1	1
1,00	13,00	7,00	2	5	4	3	2	2
1,00	13,00	7,00	2	5	5	4	1	2
1,00	13,00	6,00	2	3	4	4	1	2
1,00	13,00	6,00	2	4	4	4	2	1
1,00	13,00	6,00	2	5	4	5	2	999
1,00	13,00	5,00	2	5	3	5	2	1
1,00	14,00	5,00	2	5	4	3	1	1
1,00	13,00	4,00	2	4	5	4	1	1
1,00	13,00	4,00	2	4	2	5	1	1
1,00	14,00	4,00	2	3	4	3	5	1
1,00	14,00	3,00	2	4	3	3	3	1
1,00	13,00	2,00	2	3	3	3	2	1
1,00	13,00	11,00	2	5	4	2	2	2
1,00	13,00	11,00	2	2	2	4	3	1
1,00	13,00	11,00	2	5	4	5	1	3
1,00	13,00	11,00	2	4	5	4	3	2
1,00	13,00	11,00	2	5	4	5	2	5
1,00	13,00	11,00	2	4	5	3	2	1
1,00	13,00	8,00	2	5	5	5	1	3
1,00	13,00	8,00	2	5	5	5	1	1
1,00	14,00	8,00	2	5	4	4	1	2
1,00	15,00	8,00	2	5	5	5	1	1
1,00	13,00	7,00	2	4	3	4	2	1
1,00	13,00	7,00	2	5	5	4	3	1
1,00	13,00	7,00	2	5	5	4	2	1
1,00	13,00	7,00	2	4	5	3	2	1
1,00	13,00	7,00	2	5	5	5	4	1
1,00	15,00	7,00	2	5	4	4	3	2
1,00	14,00	7,00	2	4	4	4	1	1
1,00	13,00	6,00	2	4	4	4	1	1
1,00	12,00	6,00	2	3	2	2	3	3
1,00	13,00	6,00	2	5	5	3	2	3
1,00	13,00	6,00	2	5	3	3	2	1
1,00	13,00	6,00	2	5	4	4	1	1
1,00	13,00	5,00	2	5	3	4	3	1
1,00	13,00	5,00	2	5	4	5	2	1
1,00	13,00	5,00	2	5	5	5	1	1
1,00	13,00	5,00	2	4	3	4	3	2
1,00	13,00	5,00	2	5	4	5	1	1
1,00	13,00	5,00	2	4	4	5	1	2
1,00	14,00	5,00	2	4	5	5	999	1
1,00	14,00	4,00	2	4	4	3	1	1
1,00	13,00	4,00	2	4	5	5	2	1
1,00	13,00	4,00	2	4	3	4	2	3
1,00	13,00	4,00	2	4	3	3	3	1
1,00	13,00	4,00	2	5	4	3	3	1
1,00	13,00	3,00	2	5	4	4	1	3

1,00	13,00	3,00	2	5	3	3	3	1
1,00	13,00	3,00	2	5	5	4	1	1
1,00	13,00	3,00	2	4	3	3	3	2
1,00	14,00	3,00	2	5	4	5	2	1
1,00	13,00	2,00	2	5	4	5	1	1
1,00	13,00	2,00	2	5	5	5	3	1
1,00	13,00	2,00	2	5	5	4	3	1
1,00	13,00	2,00	2	5	5	5	3	1
1,00	13,00	11,00	2	5	5	5	1	3
1,00	13,00	11,00	2	5	5	5	2	1
1,00	13,00	11,00	2	5	4	4	1	3
1,00	13,00	11,00	2	5	5	4	3	1
1,00	14,00	11,00	2	3	4	4	1	2
1,00	13,00	8,00	2	4	5	4	5	1
1,00	14,00	8,00	2	5	5	3	1	1
1,00	15,00	8,00	2	3	3	4	2	1
1,00	13,00	7,00	2	5	4	5	1	1
1,00	13,00	7,00	2	4	4	3	2	2
1,00	13,00	7,00	2	5	5	4	2	2
1,00	13,00	7,00	2	3	2	2	1	4
1,00	14,00	7,00	2	1	3	2	5	3
1,00	14,00	7,00	2	5	5	5	3	1
1,00	999,00	7,00	2	5	5	5	1	1
1,00	13,00	6,00	2	2	3	3	4	4
1,00	13,00	6,00	2	5	2	5	2	1
1,00	13,00	6,00	2	5	5	5	1	1
1,00	13,00	6,00	2	3	5	3	2	1
1,00	13,00	6,00	2	5	4	3	1	1
1,00	14,00	6,00	2	4	5	4	1	3
1,00	14,00	6,00	2	5	3	3	4	2
1,00	13,00	5,00	2	4	3	2	2	2
1,00	13,00	5,00	2	5	3	4	2	2
1,00	13,00	5,00	2	4	3	4	2	2
1,00	13,00	5,00	2	4	5	3	3	2
1,00	13,00	5,00	2	5	5	5	2	4
1,00	13,00	4,00	2	4	4	4	2	1
1,00	13,00	4,00	2	4	5	5	2	1
1,00	13,00	4,00	2	4	3	3	2	2
1,00	13,00	4,00	2	5	4	4	2	3
1,00	14,00	4,00	2	5	4	3	1	5
1,00	13,00	3,00	2	4	5	3	2	1
1,00	13,00	3,00	2	4	4	4	1	3
1,00	13,00	3,00	2	5	4	5	1	2
1,00	14,00	3,00	2	4	5	5	2	1
1,00	14,00	3,00	2	4	5	3	2	4
1,00	14,00	3,00	2	4	5	3	1	3
1,00	13,00	2,00	2	4	4	3	2	1
1,00	13,00	2,00	2	5	5	4	2	1
1,00	13,00	2,00	2	3	4	4	2	2
1,00	14,00	2,00	2	999	999	999	999	999

1,00	14,00	11,00	3	5	3	3	1	4
1,00	14,00	3,00	3	4	3	3	2	3
1,00	14,00	3,00	3	5	4	4	5	1
1,00	14,00	11,00	3	4	5	4	2	3
1,00	14,00	11,00	3	4	4	3	2	2
1,00	14,00	8,00	3	5	5	4	2	1
1,00	14,00	8,00	3	5	5	4	1	1
1,00	14,00	8,00	3	5	4	4	3	3
1,00	14,00	7,00	3	4	4	3	3	2
1,00	14,00	6,00	3	4	4	4	3	3
1,00	14,00	6,00	3	4	4	4	1	3
1,00	14,00	4,00	3	3	3	3	2	2
1,00	14,00	3,00	3	5	5	4	2	1
1,00	15,00	2,00	3	5	4	5	4	2
1,00	14,00	11,00	3	5	4	5	1	2
1,00	14,00	11,00	3	5	4	4	1	1
1,00	14,00	11,00	3	5	5	4	1	1
1,00	15,00	11,00	3	5	4	5	2	1
1,00	14,00	11,00	3	2	1	2	2	5
1,00	14,00	8,00	3	5	4	3	3	4
1,00	14,00	8,00	3	3	2	3	3	4
1,00	14,00	7,00	3	5	4	5	2	1
1,00	14,00	6,00	3	5	2	2	4	1
1,00	14,00	6,00	3	3	5	3	1	1
1,00	14,00	6,00	3	5	4	3	5	1
1,00	15,00	6,00	3	3	3	3	2	5
1,00	15,00	6,00	3	5	4	3	1	1
1,00	14,00	5,00	3	5	4	3	2	3
1,00	14,00	5,00	3	5	4	4	2	1
1,00	14,00	5,00	3	4	2	4	5	3
1,00	15,00	4,00	3	5	4	5	1	1
1,00	15,00	4,00	3	4	5	3	3	1
1,00	14,00	3,00	3	5	5	4	1	2
1,00	14,00	3,00	3	4	5	4	1	2
1,00	15,00	3,00	3	3	3	4	1	3
1,00	15,00	3,00	3	3	5	4	2	1
1,00	15,00	3,00	3	4	5	4	1	2
1,00	14,00	2,00	3	1	5	5	1	1
1,00	14,00	11,00	3	5	4	5	2	1
1,00	14,00	11,00	3	5	5	4	1	1
1,00	14,00	11,00	3	5	5	4	1	1
1,00	14,00	11,00	3	4	5	4	1	1
1,00	14,00	11,00	3	4	4	3	1	2
1,00	14,00	11,00	3	3	4	3	1	1
1,00	14,00	11,00	3	4	4	3	1	2
1,00	14,00	11,00	3	5	4	4	3	2
1,00	14,00	11,00	3	5	4	5	1	2
1,00	15,00	11,00	3	4	3	3	2	2
1,00	15,00	11,00	3	4	3	4	3	1
1,00	15,00	11,00	3	5	4	4	2	2

1,00	14,00	8,00	3	5	5	4	2	2
1,00	14,00	8,00	3	5	5	5	5	1
1,00	14,00	8,00	3	4	2	3	2	3
1,00	14,00	8,00	3	3	4	4	3	4
1,00	14,00	8,00	3	5	5	5	3	3
1,00	14,00	8,00	3	3	4	5	2	1
1,00	14,00	8,00	3	5	4	5	1	1
1,00	14,00	8,00	3	5	5	3	1	2
1,00	15,00	8,00	3	5	4	4	1	2
1,00	15,00	8,00	3	5	3	4	3	1
1,00	15,00	7,00	3	5	4	4	1	3
1,00	15,00	7,00	3	5	5	5	1	5
1,00	15,00	7,00	3	4	4	4	4	2
1,00	14,00	6,00	3	5	5	5	3	1
1,00	14,00	6,00	3	5	5	4	1	1
1,00	14,00	6,00	3	5	4	5	2	1
1,00	14,00	6,00	3	5	5	5	1	1
1,00	14,00	5,00	3	5	4	5	1	1
1,00	14,00	5,00	3	3	5	3	2	2
1,00	15,00	5,00	3	4	3	3	1	2
1,00	15,00	5,00	3	4	3	3	1	2
1,00	14,00	4,00	3	5	4	3	1	1
1,00	14,00	4,00	3	5	4	5	2	1
1,00	14,00	4,00	3	4	5	3	3	1
1,00	14,00	3,00	3	5	5	4	2	1
1,00	14,00	3,00	3	5	4	3	1	2
1,00	14,00	3,00	3	4	5	2	1	3
1,00	14,00	3,00	3	2	4	4	3	2
1,00	15,00	3,00	3	4	5	3	2	2
1,00	15,00	3,00	3	4	5	3	1	1
1,00	999,00	3,00	3	3	3	3	2	1
1,00	14,00	2,00	3	4	999	5	1	1
1,00	14,00	2,00	3	5	5	4	1	2
1,00	14,00	2,00	3	4	4	4	1	1
1,00	14,00	11,00	3	5	5	5	1	1
1,00	14,00	11,00	3	4	4	3	3	3
1,00	14,00	11,00	3	5	5	4	2	3
1,00	14,00	11,00	3	5	5	4	3	1
1,00	14,00	11,00	3	3	3	3	1	1
1,00	15,00	11,00	3	5	4	5	2	1
1,00	15,00	11,00	3	5	4	3	1	2
1,00	14,00	8,00	3	4	3	5	1	2
1,00	14,00	8,00	3	4	3	3	2	1
1,00	14,00	8,00	3	5	4	4	2	1
1,00	14,00	8,00	3	5	4	5	1	1
1,00	14,00	8,00	3	3	3	5	1	1
1,00	14,00	8,00	3	5	5	3	1	1
1,00	15,00	8,00	3	4	5	3	2	2
1,00	16,00	8,00	3	5	5	3	3	3
1,00	16,00	8,00	3	5	5	5	3	2

1,00	15,00	7,00	3	5	3	4	1	2
1,00	14,00	6,00	3	4	4	2	5	4
1,00	14,00	6,00	3	3	5	4	2	1
1,00	14,00	6,00	3	4	4	3	1	3
1,00	14,00	6,00	3	4	3	3	1	3
1,00	15,00	6,00	3	4	4	3	1	1
1,00	14,00	6,00	3	5	4	5	1	1
1,00	15,00	6,00	3	5	5	5	1	1
1,00	14,00	5,00	3	4	5	3	3	4
1,00	14,00	5,00	3	4	3	3	2	3
1,00	15,00	5,00	3	3	5	2	1	1
1,00	14,00	4,00	3	4	4	3	999	1
1,00	14,00	4,00	3	5	5	5	2	1
1,00	14,00	3,00	3	5	4	2	2	3
1,00	14,00	3,00	3	4	4	4	2	2
1,00	14,00	3,00	3	5	4	4	2	3
1,00	14,00	3,00	3	5	5	5	2	1
1,00	14,00	3,00	3	4	3	5	2	1
1,00	14,00	3,00	3	5	4	5	1	1
1,00	14,00	3,00	3	4	5	4	3	2
1,00	14,00	3,00	3	5	3	3	2	1
1,00	14,00	3,00	3	4	5	3	3	1
1,00	14,00	3,00	3	4	4	4	2	3
1,00	14,00	3,00	3	5	3	5	2	1
1,00	14,00	3,00	3	5	3	3	1	1
1,00	15,00	3,00	3	5	4	4	2	1
1,00	14,00	2,00	3	4	5	4	2	1
1,00	14,00	2,00	3	4	3	4	4	1
1,00	14,00	2,00	3	5	3	3	4	4
1,00	15,00	6,00	4	5	3	4	2	1
1,00	15,00	2,00	4	5	4	3	4	2
1,00	15,00	11,00	4	3	2	3	4	3
1,00	15,00	11,00	4	5	3	4	2	1
1,00	16,00	11,00	4	5	5	4	1	3
1,00	15,00	6,00	4	5	4	3	2	1
1,00	16,00	4,00	4	4	4	4	5	3
1,00	15,00	2,00	4	4	4	3	5	3
1,00	15,00	2,00	4	5	4	3	3	3
1,00	15,00	2,00	4	5	4	3	1	3
1,00	15,00	11,00	4	4	5	4	2	3
1,00	15,00	8,00	4	5	5	5	2	1
1,00	16,00	8,00	4	4	5	5	4	5
1,00	15,00	7,00	4	4	4	4	2	1
1,00	15,00	7,00	4	5	5	4	1	1
1,00	15,00	7,00	4	4	3	4	4	3
1,00	15,00	7,00	4	4	2	2	5	4
1,00	16,00	7,00	4	4	4	3	3	3
1,00	15,00	6,00	4	4	4	3	2	3
1,00	15,00	6,00	4	5	5	4	2	1
1,00	16,00	6,00	4	4	2	2	3	3

1,00	16,00	4,00	4	3	5	3	3	4
1,00	15,00	3,00	4	5	4	4	2	2
1,00	15,00	3,00	4	3	4	5	2	1
1,00	15,00	3,00	4	5	3	3	2	3
1,00	15,00	3,00	4	5	4	4	1	2
1,00	15,00	2,00	4	4	3	3	2	3
1,00	15,00	11,00	4	5	5	4	2	1
1,00	15,00	11,00	4	5	4	5	2	1
1,00	15,00	11,00	4	5	5	4	4	4
1,00	15,00	11,00	4	5	3	3	2	4
1,00	15,00	11,00	4	5	4	3	2	1
1,00	16,00	11,00	4	5	3	3	3	3
1,00	16,00	11,00	4	5	5	5	1	1
1,00	15,00	8,00	4	5	4	4	3	2
1,00	16,00	8,00	4	5	4	4	2	1
1,00	15,00	7,00	4	5	4	4	2	2
1,00	15,00	7,00	4	5	4	3	1	3
1,00	15,00	7,00	4	5	3	4	2	3
1,00	15,00	6,00	4	5	5	5	3	1
1,00	15,00	6,00	4	5	1	3	5	3
1,00	15,00	4,00	4	5	5	4	1	3
1,00	15,00	4,00	4	5	4	4	2	3
1,00	16,00	4,00	4	4	5	3	3	2
1,00	15,00	3,00	4	4	4	3	2	1
1,00	15,00	3,00	4	5	4	4	2	2
1,00	15,00	3,00	4	4	4	3	2	3
1,00	15,00	3,00	4	5	4	4	1	1
1,00	15,00	3,00	4	5	4	4	2	3
1,00	15,00	3,00	4	5	3	4	2	1
1,00	15,00	2,00	4	5	5	3	1	2
1,00	15,00	2,00	4	4	4	4	1	2
1,00	15,00	2,00	4	5	5	3	2	1
1,00	15,00	11,00	4	4	4	5	1	1
1,00	15,00	11,00	4	5	5	4	3	2
1,00	15,00	11,00	4	5	5	3	1	1
1,00	15,00	11,00	4	4	3	3	2	2
1,00	16,00	11,00	4	5	5	5	3	4
1,00	16,00	11,00	4	4	4	3	3	2
1,00	16,00	11,00	4	4	4	3	2	4
1,00	15,00	8,00	4	5	4	4	2	1
1,00	15,00	8,00	4	5	4	5	2	3
1,00	15,00	8,00	4	4	4	4	1	1
1,00	15,00	7,00	4	4	3	3	2	2
1,00	15,00	7,00	4	4	4	5	2	2
1,00	15,00	7,00	4	3	4	4	2	3
1,00	15,00	7,00	4	3	4	3	2	3
1,00	16,00	7,00	4	4	5	5	1	3
1,00	16,00	6,00	4	5	5	4	2	2
1,00	15,00	4,00	4	5	5	4	2	3
1,00	16,00	4,00	4	5	5	5	1	1

1,00	16,00	4,00	4	3	5	5	2	1
1,00	15,00	3,00	4	5	4	4	3	2
1,00	15,00	3,00	4	4	4	3	3	3
1,00	15,00	3,00	4	5	4	4	2	1
1,00	15,00	3,00	4	4	3	4	4	3
1,00	15,00	3,00	4	4	5	4	1	2
1,00	15,00	2,00	4	5	5	5	2	1
1,00	16,00	2,00	4	4	3	4	2	2
1,00	16,00	2,00	4	4	3	5	3	1
2,00	12,00	3,00	1	5	3	2	4	1
2,00	12,00	11,00	1	4	4	4	2	5
2,00	12,00	8,00	1	3	3	999	4	1
2,00	14,00	8,00	1	4	4	3	3	2
2,00	12,00	6,00	1	3	3	2	2	1
2,00	12,00	6,00	1	3	4	5	2	3
2,00	12,00	6,00	1	4	3	5	4	1
2,00	12,00	11,00	1	3	3	4	1	1
2,00	12,00	11,00	1	3	4	4	2	2
2,00	12,00	11,00	1	4	5	3	1	2
2,00	12,00	11,00	1	4	3	3	3	3
2,00	12,00	11,00	1	5	4	4	2	3
2,00	13,00	11,00	1	3	4	3	2	1
2,00	12,00	8,00	1	2	2	4	1	1
2,00	12,00	7,00	1	4	3	4	2	1
2,00	12,00	6,00	1	1	2	1	1	1
2,00	12,00	6,00	1	5	3	4	2	2
2,00	12,00	6,00	1	3	3	3	4	2
2,00	12,00	6,00	1	3	2	2	4	4
2,00	12,00	6,00	1	2	1	2	1	1
2,00	13,00	6,00	1	5	4	3	1	1
2,00	12,00	5,00	1	3	4	3	1	1
2,00	13,00	4,00	1	4	2	2	4	5
2,00	13,00	4,00	1	3	5	4	4	1
2,00	12,00	3,00	1	4	5	3	5	2
2,00	12,00	3,00	1	3	4	3	1	3
2,00	12,00	3,00	1	5	5	4	3	2
2,00	12,00	3,00	1	4	3	3	2	2
2,00	12,00	2,00	1	4	4	3	2	1
2,00	12,00	11,00	1	3	4	2	1	3
2,00	12,00	11,00	1	3	5	3	1	2
2,00	12,00	11,00	1	4	3	2	5	3
2,00	12,00	11,00	1	5	4	3	3	3
2,00	12,00	11,00	1	4	3	4	2	1
2,00	12,00	11,00	1	3	4	4	1	5
2,00	13,00	8,00	1	5	5	4	2	1
2,00	12,00	8,00	1	5	1	4	3	5
2,00	12,00	8,00	1	2	4	1	1	5
2,00	13,00	8,00	1	2	3	2	1	1
2,00	14,00	8,00	1	4	5	4	2	3
2,00	12,00	6,00	1	5	3	1	1	1

2,00	12,00	6,00	1	4	3	3	2	1
2,00	12,00	6,00	1	4	3	3	2	1
2,00	12,00	6,00	1	4	2	4	2	3
2,00	12,00	6,00	1	4	3	3	4	4
2,00	12,00	5,00	1	2	3	3	2	1
2,00	12,00	4,00	1	1	2	2	4	5
2,00	13,00	4,00	1	3	1	2	5	1
2,00	12,00	3,00	1	4	5	5	2	1
2,00	12,00	3,00	1	5	3	4	2	1
2,00	13,00	3,00	1	4	2	3	1	2
2,00	12,00	2,00	1	4	3	2	3	2
2,00	12,00	2,00	1	5	4	4	3	1
2,00	12,00	11,00	1	5	5	2	4	5
2,00	12,00	11,00	1	3	5	3	1	1
2,00	12,00	11,00	1	3	4	4	3	5
2,00	12,00	11,00	1	5	4	5	2	1
2,00	12,00	11,00	1	5	5	4	1	1
2,00	12,00	11,00	1	5	3	4	3	5
2,00	12,00	11,00	1	5	4	4	1	2
2,00	12,00	11,00	1	4	5	3	2	4
2,00	13,00	11,00	1	4	5	4	1	2
2,00	13,00	11,00	1	4	3	5	1	2
2,00	12,00	8,00	1	4	5	3	1	1
2,00	12,00	8,00	1	3	4	4	3	1
2,00	12,00	8,00	1	5	5	5	5	1
2,00	12,00	8,00	1	5	5	5	1	1
2,00	14,00	8,00	1	5	3	3	1	1
2,00	14,00	8,00	1	3	5	4	4	1
2,00	12,00	7,00	1	4	4	4	1	1
2,00	12,00	7,00	1	4	5	5	2	1
2,00	12,00	7,00	1	3	4	5	1	1
2,00	12,00	7,00	1	3	5	4	2	1
2,00	13,00	7,00	1	3	4	4	1	1
2,00	12,00	6,00	1	3	4	3	3	1
2,00	12,00	6,00	1	5	5	4	5	1
2,00	14,00	6,00	1	3	4	2	2	1
2,00	999,00	5,00	1	4	3	1	2	3
2,00	12,00	4,00	1	3	4	5	1	4
2,00	12,00	4,00	1	5	5	3	2	2
2,00	12,00	4,00	1	1	1	5	1	4
2,00	12,00	4,00	1	5	999	4	1	1
2,00	13,00	4,00	1	3	4	2	3	5
2,00	12,00	3,00	1	4	5	4	1	5
2,00	12,00	3,00	1	3	3	4	2	3
2,00	12,00	3,00	1	4	3	3	2	2
2,00	12,00	3,00	1	4	5	3	2	1
2,00	12,00	3,00	1	3	3	2	3	1
2,00	12,00	3,00	1	4	3	4	2	1
2,00	12,00	3,00	1	5	3	3	4	3
2,00	12,00	2,00	1	5	3	5	1	1

2,00	12,00	2,00	1	5	5	1	4	5
2,00	12,00	2,00	1	5	4	4	3	1
2,00	12,00	2,00	1	4	3	3	2	2
2,00	15,00	8,00	2	5	4	5	1	1
2,00	14,00	11,00	2	3	3	2	2	1
2,00	13,00	8,00	2	3	4	3	2	1
2,00	14,00	8,00	2	3	3	3	5	5
2,00	13,00	3,00	2	4	3	3	1	1
2,00	13,00	11,00	2	4	3	3	2	2
2,00	13,00	11,00	2	5	4	3	1	1
2,00	13,00	8,00	2	3	4	3	1	3
2,00	13,00	8,00	2	5	4	4	4	1
2,00	999,00	8,00	2	3	3	3	3	3
2,00	14,00	6,00	2	4	3	2	4	1
2,00	13,00	11,00	2	4	4	5	1	1
2,00	13,00	11,00	2	5	4	5	1	1
2,00	13,00	11,00	2	4	5	4	3	2
2,00	13,00	8,00	2	4	4	3	2	5
2,00	13,00	8,00	2	4	3	3	2	1
2,00	14,00	8,00	2	4	5	4	2	4
2,00	14,00	8,00	2	2	3	2	1	1
2,00	15,00	8,00	2	3	4	4	3	5
2,00	13,00	7,00	2	3	2	4	1	2
2,00	13,00	7,00	2	2	4	999	3	1
2,00	13,00	7,00	2	5	4	3	3	2
2,00	13,00	6,00	2	4	4	4	3	2
2,00	14,00	6,00	2	4	3	4	2	4
2,00	14,00	5,00	2	3	4	3	1	2
2,00	14,00	4,00	2	4	3	3	2	2
2,00	14,00	4,00	2	4	4	3	2	2
2,00	14,00	4,00	2	5	4	3	1	1
2,00	13,00	3,00	2	3	5	4	2	1
2,00	13,00	3,00	2	4	3	4	2	1
2,00	13,00	2,00	2	5	5	3	2	2
2,00	13,00	2,00	2	4	3	3	1	2
2,00	13,00	2,00	2	5	4	5	2	1
2,00	13,00	11,00	2	3	1	4	2	1
2,00	13,00	11,00	2	4	3	5	1	1
2,00	13,00	11,00	2	3	4	2	1	4
2,00	13,00	11,00	2	4	3	3	3	5
2,00	13,00	11,00	2	3	3	2	1	2
2,00	13,00	11,00	2	4	4	3	1	2
2,00	13,00	11,00	2	4	3	5	1	1
2,00	13,00	11,00	2	4	3	3	1	2
2,00	14,00	11,00	2	4	5	3	1	1
2,00	14,00	11,00	2	5	2	1	3	3
2,00	13,00	8,00	2	4	4	3	2	4
2,00	13,00	8,00	2	3	4	3	1	1
2,00	13,00	8,00	2	4	4	3	3	2
2,00	13,00	8,00	2	4	3	4	1	1

2,00	13,00	8,00	2	5	4	4	2	1
2,00	13,00	8,00	2	4	3	3	1	1
2,00	14,00	8,00	2	4	3	5	3	1
2,00	14,00	8,00	2	4	3	3	2	3
2,00	14,00	8,00	2	3	3	3	3	3
2,00	13,00	7,00	2	5	5	5	1	5
2,00	13,00	7,00	2	1	2	1	2	2
2,00	13,00	7,00	2	5	5	3	2	1
2,00	13,00	7,00	2	4	4	4	2	1
2,00	14,00	7,00	2	4	1	2	5	1
2,00	14,00	7,00	2	3	4	4	1	1
2,00	14,00	7,00	2	3	4	5	1	5
2,00	14,00	7,00	2	2	3	3	2	3
2,00	15,00	7,00	2	4	3	4	2	1
2,00	13,00	6,00	2	3	3	3	3	3
2,00	13,00	6,00	2	4	3	999	999	1
2,00	13,00	6,00	2	3	3	4	3	2
2,00	14,00	6,00	2	4	4	4	2	1
2,00	14,00	6,00	2	3	3	2	3	1
2,00	14,00	6,00	2	5	5	5	1	5
2,00	14,00	6,00	2	4	5	5	3	1
2,00	13,00	5,00	2	3	3	4	2	1
2,00	14,00	5,00	2	1	2	1	1	3
2,00	14,00	5,00	2	2	3	4	1	3
2,00	15,00	5,00	2	5	5	5	3	2
2,00	13,00	4,00	2	4	4	3	2	4
2,00	14,00	4,00	2	4	4	3	2	1
2,00	14,00	4,00	2	4	3	4	2	1
2,00	13,00	3,00	2	4	5	4	2	3
2,00	13,00	3,00	2	4	4	3	2	1
2,00	13,00	3,00	2	3	5	4	2	1
2,00	13,00	3,00	2	3	5	4	2	2
2,00	14,00	3,00	2	4	4	4	2	2
2,00	14,00	3,00	2	2	3	4	1	4
2,00	14,00	3,00	2	4	4	5	2	1
2,00	13,00	2,00	2	4	3	3	2	1
2,00	14,00	2,00	2	5	4	3	3	1
2,00	14,00	2,00	2	5	4	5	2	1
2,00	14,00	2,00	2	3	3	2	1	1
2,00	15,00	2,00	2	5	3	2	1	3
2,00	14,00	8,00	3	2	3	3	4	2
2,00	14,00	2,00	3	4	3	3	2	2
2,00	14,00	8,00	3	5	4	4	3	1
2,00	14,00	11,00	3	5	5	5	1	3
2,00	15,00	11,00	3	5	4	4	2	1
2,00	15,00	11,00	3	5	3	4	2	2
2,00	14,00	8,00	3	3	5	4	2	1
2,00	15,00	8,00	3	5	4	3	2	1
2,00	14,00	7,00	3	3	4	3	3	2
2,00	14,00	6,00	3	3	4	3	4	3

2,00	15,00	6,00	3	3	3	2	4	3
2,00	15,00	6,00	3	3	4	3	2	1
2,00	15,00	6,00	3	3	3	2	1	1
2,00	15,00	6,00	3	4	3	3	2	3
2,00	15,00	6,00	3	2	3	2	2	1
2,00	14,00	4,00	3	3	3	2	999	2
2,00	15,00	2,00	3	4	4	3	1	1
2,00	14,00	11,00	3	3	3	3	3	1
2,00	14,00	11,00	3	5	4	3	1	3
2,00	14,00	11,00	3	4	3	3	2	2
2,00	14,00	11,00	3	4	3	4	1	3
2,00	14,00	11,00	3	4	2	5	1	1
2,00	14,00	11,00	3	3	2	4	2	1
2,00	14,00	11,00	3	5	4	3	2	4
2,00	14,00	11,00	3	4	3	5	2	1
2,00	15,00	11,00	3	5	3	3	2	3
2,00	14,00	8,00	3	5	3	4	4	4
2,00	14,00	8,00	3	2	3	3	2	1
2,00	15,00	8,00	3	4	3	4	3	3
2,00	15,00	8,00	3	5	5	4	1	4
2,00	15,00	8,00	3	5	5	5	1	1
2,00	15,00	8,00	3	5	5	5	1	1
2,00	14,00	7,00	3	5	4	3	2	1
2,00	14,00	7,00	3	5	4	3	4	2
2,00	14,00	6,00	3	2	2	2	3	2
2,00	15,00	6,00	3	3	5	4	3	1
2,00	14,00	5,00	3	5	3	4	1	1
2,00	14,00	5,00	3	3	3	2	2	4
2,00	14,00	5,00	3	4	2	3	2	1
2,00	14,00	3,00	3	5	3	3	3	3
2,00	14,00	3,00	3	3	2	3	2	3
2,00	15,00	3,00	3	4	5	4	3	3
2,00	15,00	3,00	3	4	2	4	3	1
2,00	15,00	3,00	3	3	4	4	2	2
2,00	14,00	2,00	3	4	5	3	2	1
2,00	14,00	11,00	3	4	3	4	2	2
2,00	14,00	11,00	3	4	5	3	2	5
2,00	14,00	11,00	3	3	3	4	3	2
2,00	14,00	11,00	3	4	4	5	2	3
2,00	14,00	11,00	3	3	5	3	2	1
2,00	14,00	11,00	3	5	3	4	2	2
2,00	14,00	11,00	3	4	5	4	4	3
2,00	15,00	11,00	3	2	5	3	1	3
2,00	15,00	11,00	3	4	4	4	2	2
2,00	14,00	8,00	3	4	4	4	3	3
2,00	14,00	8,00	3	4	5	4	2	1
2,00	14,00	8,00	3	4	5	4	3	3
2,00	14,00	8,00	3	3	4	3	2	1
2,00	15,00	8,00	3	4	2	3	2	1
2,00	15,00	8,00	3	4	4	5	3	2

2,00	15,00	8,00	3	5	5	4	3	1
2,00	15,00	8,00	3	5	3	4	2	4
2,00	15,00	8,00	3	2	3	1	2	1
2,00	16,00	8,00	3	2	4	3	1	1
2,00	17,00	8,00	3	5	3	5	1	2
2,00	17,00	8,00	3	5	4	4	2	1
2,00	14,00	7,00	3	4	3	4	2	1
2,00	15,00	7,00	3	3	5	4	1	1
2,00	14,00	6,00	3	5	5	4	3	5
2,00	14,00	6,00	3	4	3	4	2	2
2,00	14,00	5,00	3	3	4	4	1	3
2,00	14,00	5,00	3	2	3	4	2	1
2,00	14,00	5,00	3	3	4	4	1	2
2,00	14,00	5,00	3	3	4	3	1	3
2,00	14,00	5,00	3	5	4	5	2	2
2,00	14,00	5,00	3	4	3	4	2	2
2,00	14,00	5,00	3	4	3	5	2	1
2,00	15,00	5,00	3	2	4	2	2	3
2,00	14,00	4,00	3	5	4	4	2	2
2,00	14,00	4,00	3	3	4	3	2	1
2,00	15,00	4,00	3	3	4	2	2	2
2,00	14,00	3,00	3	4	4	4	2	2
2,00	14,00	3,00	3	4	4	4	2	3
2,00	14,00	3,00	3	5	2	3	2	1
2,00	14,00	3,00	3	5	3	4	1	4
2,00	14,00	3,00	3	5	4	5	1	1
2,00	14,00	3,00	3	4	4	3	2	2
2,00	14,00	3,00	3	4	4	3	2	2
2,00	14,00	3,00	3	3	4	5	3	3
2,00	14,00	3,00	3	4	4	5	2	1
2,00	14,00	3,00	3	5	4	2	3	2
2,00	14,00	3,00	3	4	3	1	1	1
2,00	15,00	3,00	3	4	3	4	1	1
2,00	17,00	3,00	3	2	2	3	1	4
2,00	14,00	2,00	3	4	4	4	3	3
2,00	14,00	2,00	3	4	4	5	3	3
2,00	15,00	6,00	4	2	3	2	2	3
2,00	16,00	6,00	4	4	4	3	1	4
2,00	17,00	6,00	4	5	5	4	2	5
2,00	15,00	3,00	4	5	2	3	3	4
2,00	15,00	3,00	4	5	5	3	1	5
2,00	16,00	3,00	4	4	3	3	2	1
2,00	15,00	7,00	4	3	3	2	1	1
2,00	16,00	6,00	4	4	5	5	1	1
2,00	15,00	6,00	4	4	4	4	2	1
2,00	15,00	4,00	4	5	5	5	1	1
2,00	16,00	3,00	4	5	4	3	3	2
2,00	15,00	2,00	4	3	4	3	4	2
2,00	15,00	11,00	4	5	4	4	2	3
2,00	16,00	11,00	4	3	4	4	1	1

2,00	16,00	11,00	4	4	3	2	1	3
2,00	16,00	11,00	4	4	3	4	2	1
2,00	15,00	8,00	4	3	3	3	4	2
2,00	16,00	8,00	4	5	1	5	1	1
2,00	15,00	7,00	4	3	4	3	5	1
2,00	16,00	6,00	4	4	3	4	2	4
2,00	15,00	3,00	4	5	4	4	2	1
2,00	15,00	3,00	4	4	5	4	1	1
2,00	15,00	3,00	4	1	2	2	2	2
2,00	15,00	3,00	4	2	4	3	1	4
2,00	15,00	3,00	4	3	4	4	1	3
2,00	15,00	3,00	4	5	4	3	2	2
2,00	15,00	3,00	4	3	3	3	2	1
2,00	15,00	3,00	4	5	3	4	2	1
2,00	16,00	3,00	4	3	4	4	2	4
2,00	16,00	2,00	4	5	5	4	1	1
2,00	15,00	11,00	4	4	3	4	2	1
2,00	15,00	11,00	4	5	4	3	3	3
2,00	15,00	11,00	4	5	3	4	1	1
2,00	15,00	11,00	4	3	4	3	4	3
2,00	15,00	11,00	4	4	2	3	3	2
2,00	15,00	11,00	4	4	3	4	3	4
2,00	15,00	8,00	4	2	3	3	2	1
2,00	16,00	8,00	4	4	5	3	1	1
2,00	15,00	7,00	4	4	3	3	2	2
2,00	15,00	7,00	4	3	3	3	2	4
2,00	15,00	7,00	4	999	999	999	999	999
2,00	15,00	7,00	4	5	4	4	2	3
2,00	16,00	7,00	4	3	3	3	4	2
2,00	16,00	7,00	4	4	4	4	2	2
2,00	15,00	6,00	4	4	2	5	3	2
2,00	15,00	5,00	4	5	4	4	3	3
2,00	15,00	5,00	4	4	5	4	2	2
2,00	15,00	4,00	4	1	2	2	2	4
2,00	15,00	4,00	4	4	3	3	2	1
2,00	999,00	4,00	4	3	2	2	2	4
2,00	15,00	3,00	4	5	5	5	2	2
2,00	15,00	3,00	4	3	3	4	3	2
2,00	15,00	3,00	4	3	3	4	2	2
2,00	15,00	3,00	4	4	3	4	2	1
2,00	15,00	3,00	4	4	3	4	2	4
2,00	15,00	3,00	4	4	4	5	2	1
2,00	15,00	3,00	4	4	3	2	3	2
2,00	15,00	3,00	4	5	5	4	2	2
2,00	15,00	3,00	4	4	5	3	1	2
2,00	15,00	3,00	4	4	4	4	2	2
2,00	15,00	3,00	4	5	4	5	1	1
2,00	15,00	3,00	4	4	4	4	2	4
2,00	15,00	3,00	4	4	2	4	1	5
2,00	15,00	3,00	4	4	4	3	2	2

2,00	15,00	3,00	4	5	4	5	1	1
2,00	15,00	3,00	4	4	2	4	1	1
2,00	15,00	3,00	4	4	4	4	2	5
2,00	15,00	3,00	4	5	4	4	2	1
2,00	16,00	3,00	4	4	4	5	1	1
2,00	16,00	3,00	4	5	5	999	4	1
2,00	16,00	3,00	4	3	4	4	2	1
2,00	16,00	3,00	4	4	5	3	2	3
2,00	15,00	2,00	4	2	2	1	2	1
2,00	15,00	2,00	4	2	2	4	1	1
2,00	16,00	2,00	4	3	3	4	1	5
999,00	999,00	7,00	1	4	4	3	2	2
999,00	13,00	5,00	1	5	5	4	2	2
999,00	14,00	8,00	3	5	5	5	2	1
999,00	14,00	2,00	3	3	4	4	2	1
999,00	15,00	11,00	4	4	5	5	1	2

IPPA_A6	IPPA_A7	IPPA_A8	IPPA_A9	IPPA_A10	IPPA_A11	IPPA_A12	IPPA_A13	IPPA_A14
5	2	5	5	1	1	5	5	1
5	3	5	3	2	1	4	5	1
4	3	4	3	4	2	3	5	3
5	5	5	5	1	1	5	5	5
4	2	3	4	5	2	3	5	3
4	4	5	3	2	1	5	5	1
5	1	5	4	3	1	5	4	5
4	4	5	5	1	3	3	5	5
2	3	4	5	4	3	2	5	2
5	5	5	5	2	1	5	5	5
2	3	4	2	2	2	2	2	3
5	5	5	4	1	1	999	5	5
3	2	5	4	999	2	2	4	4
4	5	5	5	3	2	4	4	999
5	3	5	3	1	1	5	5	5
4	3	5	4	1	1	4	5	4
5	1	5	2	2	1	5	5	5
4	4	5	5	2	1	5	5	5
4	2	3	999	4	4	2	999	2
3	2	4	3	4	2	3	4	3
5	2	5	4	1	4	5	5	5
2	4	4	5	4	2	3	4	4
4	2	4	5	2	2	4	5	4
4	4	3	3	2	4	4	5	3
4	3	5	4	1	1	5	5	4
4	4	5	3	2	1	4	5	5
4	4	3	3	3	2	4	4	4
5	5	5	4	3	2	4	5	4
2	3	4	5	4	4	3	5	3
4	3	5	5	4	1	5	5	5
5	5	5	999	1	1	5	5	5
999	999	999	4	999	999	999	4	999
3	4	5	1	1	1	5	5	3
5	4	5	5	4	1	4	5	5
3	5	5	3	3	2	5	4	4
3	4	1	2	2	4	3	3	2
3	3	4	3	2	2	3	4	3
4	4	5	5	5	1	4	5	4
4	4	5	4	4	2	4	4	4
3	4	5	5	3	1	4	5	5
5	4	5	1	1	1	4	5	5
5	5	5	1	5	1	5	5	5
3	1	4	2	1	3	3	4	4
5	4	5	3	1	1	5	5	5
4	2	4	3	2	2	4	4	4
2	3	2	5	5	5	2	3	2
5	3	5	4	3	1	5	5	5
4	3	5	4	2	2	4	4	4
5	3	5	5	3	5	5	5	4

3	3	4	4	3	2	4	3	5
5	4	4	5	4	2	1	5	4
3	5	1	5	1	4	5	5	4
5	1	5	3	5	2	4	5	4
5	5	5	3	5	1	3	5	5
5	3	5	4	1	1	3	5	4
1	3	4	4	5	5	4	4	3
3	4	5	5	3	2	5	5	3
4	4	5	4	3	1	5	5	5
2	3	5	4	2	1	1	4	5
5	5	5	5	1	1	5	5	5
5	5	5	4	1	1	4	5	5
3	3	3	1	1	3	3	3	3
4	5	4	4	2	2	4	4	4
5	5	5	4	3	2	4	5	4
3	2	5	3	3	1	4	5	3
4	3	4	5	3	2	4	4	4
4	3	4	3	2	3	4	4	4
4	3	5	4	1	1	3	4	3
5	2	5	3	3	1	5	5	5
4	5	5	3	5	1	4	4	4
4	2	5	4	3	1	4	5	5
4	3	5	5	2	2	4	5	4
2	3	4	5	3	1	3	3	3
5	4	5	3	4	1	4	5	4
3	3	5	3	3	1	4	5	5
5	4	5	2	1	1	4	5	5
4	5	4	2	2	3	4	4	5
4	4	4	5	4	1	4	5	4
3	5	5	4	3	5	5	5	5
4	5	5	1	1	3	4	5	5
4	2	5	4	4	4	3	5	4
5	5	5	5	1	1	5	5	5
1	1	2	4	5	5	1	1	1
2	2	3	4	4	4	3	3	4
5	5	5	5	1	1	3	5	5
5	5	5	4	1	1	5	4	5
5	5	5	3	2	1	5	5	5
4	4	5	3	1	1	4	5	5
4	4	5	5	1	2	5	5	5
5	4	5	4	5	4	3	3	5
3	3	3	2	1	1	3	3	3
4	3	4	5	2	1	4	5	4
5	5	5	4	1	1	5	5	5
5	3	5	5	4	3	4	5	5
4	4	5	4	2	1	4	5	3
4	4	5	5	4	1	5	5	5
5	5	5	5	1	1	1	5	1
3	5	5	4	1	1	5	5	2
4	3	5	1	1	1	5	5	5

5	3	5	4	1	3	4	5	1
5	5	5	5	1	1	5	5	5
5	4	5	5	1	1	5	5	1
5	4	5	5	5	1	4	5	5
5	5	5	4	3	1	5	5	5
5	5	5	5	1	1	5	5	5
4	4	4	3	2	2	3	4	5
5	5	5	5	2	1	5	5	5
2	2	5	4	5	2	2	3	2
5	5	5	1	1	1	5	5	5
4	2	5	3	2	1	5	3	4
4	5	5	4	2	5	5	5	4
5	5	5	5	1	1	5	5	5
3	2	5	5	1	2	4	4	4
4	2	5	3	1	1	5	5	4
4	4	4	5	4	1	5	5	5
4	4	5	4	3	2	4	4	5
4	4	5	3	2	1	4	5	5
5	5	5	3	5	5	4	5	3
2	1	3	2	3	3	5	3	3
5	5	4	5	1	1	5	5	5
3	999	5	2	3	1	4	5	4
5	3	5	1	1	1	5	5	5
4	3	5	3	3	1	4	5	5
4	5	4	3	2	2	4	5	4
5	5	5	5	1	1	3	5	5
4	3	5	2	3	2	4	5	4
5	5	5	5	1	1	5	5	5
2	2	4	3	1	1	5	4	5
5	3	4	5	4	3	2	5	5
3	3	3	999	3	2	5	5	4
5	5	5	4	1	1	5	5	5
3	4	4	5	5	1	4	3	4
4	4	4	4	5	2	4	4	3
4	3	5	4	3	2	3	4	5
4	4	5	4	2	3	2	5	4
4	4	4	4	4	1	4	5	5
5	5	4	5	5	4	5	3	5
4	5	5	3	1	1	5	5	4
4	5	5	5	1	2	4	5	2
5	4	5	4	1	1	5	5	5
4	2	5	3	2	2	5	5	5
3	5	5	4	3	2	4	5	5
2	2	5	3	5	2	4	4	4
4	4	5	3	2	2	4	5	3
4	4	5	4	3	1	4	5	5
2	3	5	4	5	1	4	3	3
5	5	5	5	4	1	5	5	5
3	2	4	5	3	3	3	5	3
5	4	4	5	4	1	3	5	5

4	2	5	4	2	1	3	5	5
4	5	5	4	3	1	4	5	4
5	3	5	4	2	2	5	5	4
5	5	5	5	2	2	4	5	5
4	3	5	5	3	1	5	4	4
5	3	5	2	1	1	5	5	5
4	3	4	2	4	2	4	3	4
5	4	5	4	3	1	5	5	5
4	2	5	3	1	1	5	5	4
4	5	5	4	2	1	5	5	4
4	3	4	5	3	2	5	5	4
5	4	5	5	2	1	5	5	4
3	3	4	4	5	5	3	4	3
3	2	3	4	2	3	3	4	3
4	3	4	4	3	3	4	5	4
2	5	4	3	4	2	2	4	3
3	3	4	2	3	2	5	5	3
5	4	5	3	1	1	5	5	4
4	5	5	3	1	1	4	5	5
3	4	5	3	4	3	4	4	5
4	4	5	5	2	2	4	5	3
5	5	5	5	1	1	5	5	5
5	5	5	5	1	1	5	5	5
4	5	5	2	1	1	5	5	4
5	5	5	5	1	1	5	5	1
4	3	5	3	1	1	5	5	4
4	2	5	4	2	2	4	5	4
5	5	5	5	2	1	5	5	5
3	4	5	3	4	2	4	5	4
5	4	5	4	1	1	4	5	5
4	4	5	4	2	1	4	5	3
3	4	5	4	4	1	4	3	3
4	3	5	2	2	2	5	5	5
2	2	3	3	4	3	2	3	3
3	3	3	4	2	2	3	3	3
4	4	4	5	2	1	4	5	5
5	5	5	5	1	1	4	5	5
5	4	5	3	2	1	4	5	5
4	4	5	5	1	1	5	5	4
5	5	5	3	1	1	5	5	5
4	3	5	4	3	3	3	5	2
4	3	5	5	1	1	5	5	5
4	3	5	3	2	1	4	5	5
5	5	5	4	2	1	5	5	4
5	4	4	4	2	2	4	4	4
5	3	5	5	4	1	5	5	4
5	4	5	3	2	2	4	4	4
3	2	5	5	3	1	5	5	5
4	3	5	5	2	1	5	5	5
5	5	5	5	4	2	5	5	5

4	3	5	4	2	1	4	5	4
5	5	5	5	1	1	5	5	5
3	4	4	4	2	2	4	4	3
5	5	5	5	1	1	5	5	5
5	4	5	4	1	1	4	5	5
4	5	5	5	3	1	5	5	5
4	999	5	4	4	1	4	5	5
4	5	5	5	4	1	4	5	5
5	5	5	5	3	1	5	5	5
5	5	5	5	1	5	5	5	5
4	3	5	5	3	1	4	5	3
5	2	5	5	2	1	5	5	5
4	3	4	3	1	2	4	4	5
4	5	5	2	4	2	3	5	5
5	5	5	5	1	1	5	5	5
3	4	5	3	2	1	5	5	4
5	3	5	5	1	1	5	5	5
4	3	4	4	5	1	4	4	4
5	5	5	5	2	1	4	5	4
3	1	5	2	1	1	3	4	5
2	4	3	1	4	4	2	3	4
4	5	5	4	3	1	5	5	5
5	5	5	4	4	1	5	5	5
3	2	3	2	2	3	3	3	4
4	4	5	5	4	1	5	4	5
5	5	5	5	2	1	5	5	4
4	4	5	3	1	2	5	5	4
5	5	5	5	3	3	3	5	5
5	5	5	3	1	1	5	5	5
5	5	5	5	2	2	5	5	5
3	3	4	3	3	3	3	3	4
3	4	4	2	3	2	5	4	4
4	4	4	4	2	1	5	5	5
3	4	5	4	3	1	4	5	4
4	4	5	5	3	2	4	4	4
4	5	5	3	1	1	4	5	4
4	3	5	4	3	1	4	5	5
4	3	5	4	3	1	3	3	3
5	5	5	4	2	1	4	5	4
4	4	5	4	2	2	5	4	5
4	3	4	4	2	2	4	4	4
5	4	5	5	1	1	4	5	4
4	5	5	3	2	1	5	5	4
5	4	5	3	2	1	5	5	5
3	4	4	4	3	4	2	4	4
5	5	5	4	3	1	5	5	5
4	3	5	5	4	3	4	5	4
4	4	4	2	4	2	5	5	5
3	2	5	4	2	3	5	4	3
999	999	999	999	999	999	999	999	999

1	2	4	2	5	5	3	3	3
3	3	2	2	3	2	3	4	3
3	4	5	5	3	1	5	5	4
4	3	5	3	2	1	5	5	4
4	4	4	2	4	3	4	4	3
4	5	5	5	3	4	5	5	4
4	4	5	4	3	3	5	5	5
4	5	3	5	2	2	4	5	5
3	2	3	3	3	2	3	4	4
3	2	3	4	3	3	3	3	4
5	4	5	4	2	2	5	4	4
3	4	3	3	1	2	3	5	2
4	5	5	2	1	1	5	5	4
4	4	5	5	3	4	4	5	4
4	5	5	5	4	1	5	5	5
5	5	4	3	2	2	5	5	4
5	5	5	1	5	1	5	5	5
5	5	5	4	2	1	5	5	5
1	1	2	3	3	3	3	1	4
3	2	4	5	3	5	4	3	5
2	2	4	3	5	4	2	2	3
5	4	5	2	1	1	4	5	4
2	1	5	2	4	2	2	3	2
4	3	5	1	999	1	3	5	4
5	5	5	5	3	3	5	5	4
3	4	5	5	4	1	3	3	4
4	5	5	5	2	2	4	5	4
3	3	4	4	3	2	3	4	3
5	3	5	4	1	3	4	5	4
3	5	5	2	4	3	4	5	4
4	5	5	4	2	2	5	5	4
5	3	5	5	5	1	4	5	1
4	5	5	3	2	1	5	5	999
4	4	5	4	3	1	5	4	5
4	3	5	5	4	3	4	5	5
4	5	5	2	3	999	5	4	4
4	3	5	3	2	2	4	4	4
5	1	5	1	999	1	5	5	5
4	5	5	4	3	1	5	5	5
5	5	5	5	1	1	5	5	5
4	3	5	1	3	1	4	5	5
4	4	5	4	1	1	5	5	4
4	3	5	3	2	2	4	4	4
4	3	5	3	5	2	4	5	4
4	3	5	3	2	2	4	4	4
4	3	5	4	2	1	4	3	4
4	5	5	3	2	1	5	5	4
4	3	5	3	3	2	4	5	4
3	2	5	4	3	1	5	5	5
4	3	5	3	2	2	5	4	5

5	4	5	5	1	1	5	5	5
5	1	5	5	1	1	3	5	5
3	2	4	5	4	4	3	3	3
2	1	3	4	5	3	3	4	4
4	4	5	5	3	2	2	4	5
999	5	5	4	1	1	5	5	5
4	3	5	5	2	1	4	5	3
4	4	5	4	3	1	3	5	4
4	5	5	3	1	1	4	5	3
5	4	5	2	3	2	4	4	4
4	5	4	3	2	1	4	4	4
4	5	5	2	1	1	4	4	5
4	5	5	4	1	2	4	5	4
5	5	5	5	1	1	5	5	5
5	5	5	5	3	1	5	5	5
4	5	5	4	3	2	4	5	5
5	5	5	5	3	1	5	5	5
4	5	5	3	1	2	5	5	4
3	4	5	4	3	1	5	5	5
2	5	5	3	1	1	4	3	4
5	1	4	5	2	1	4	5	1
4	4	5	5	3	1	4	5	5
5	4	5	5	3	2	5	5	5
5	3	5	5	3	1	4	5	1
5	4	5	3	1	1	5	5	5
4	5	5	3	1	2	5	5	5
3	2	4	2	5	2	3	3	3
3	3	4	3	2	2	4	4	2
4	4	5	5	2	2	4	5	5
4	4	5	5	2	1	5	5	5
3	2	4	4	3	1	4	5	5
4	3	4	5	2	2	4	4	4
5	4	5	5	4	2	5	5	5
4	3	4	4	5	3	4	4	4
4	4	5	5	2	1	4	5	4
4	2	5	4	3	1	4	4	4
5	4	5	4	2	1	5	5	2
4	5	4	3	2	2	4	5	5
4	3	5	3	2	1	5	5	5
5	5	5	5	1	1	4	5	5
5	4	5	4	1	1	5	5	5
5	4	5	5	1	1	5	5	5
4	3	4	5	3	2	4	4	4
5	4	5	3	1	1	5	5	4
4	4	5	5	1	1	5	5	5
4	4	5	5	1	1	5	5	5
5	4	5	5	1	1	5	5	5
4	3	4	4	4	2	5	5	5
3	3	4	4	3	1	5	5	5
5	5	5	4	3	1	5	5	5

4	3	4	3	3	1	5	4	5
5	1	5	5	1	3	3	5	2
3	4	5	3	2	2	5	4	5
3	3	5	3	2	3	4	4	5
3	4	5	4	2	4	3	4	5
4	4	5	3	1	1	4	4	3
4	4	5	5	1	1	4	5	4
5	5	5	5	2	1	5	5	5
3	2	4	4	4	3	5	4	4
4	5	5	3	1	1	4	4	5
5	5	5	5	1	1	5	5	5
4	3	5	5	3	1	5	5	5
4	5	5	5	2	2	5	5	4
3	2	4	3	2	2	4	5	5
4	2	4	5	3	2	4	4	4
4	4	5	2	3	1	5	5	5
4	3	5	4	2	1	5	5	5
5	3	5	5	1	1	5	5	5
5	1	5	2	1	1	4	5	4
4	3	4	4	999	2	5	4	5
4	4	5	4	1	2	4	4	3
4	5	4	4	3	2	4	5	3
4	3	5	3	3	2	5	4	4
5	5	5	4	1	1	5	5	5
4	3	5	3	2	1	4	5	5
4	3	5	2	2	2	4	4	4
4	5	5	5	2	2	5	5	5
5	4	5	5	4	1	5	5	5
3	4	5	5	4	2	5	5	4
4	4	5	4	2	1	4	5	5
4	3	4	5	4	1	5	5	5
3	1	5	4	3	3	2	5	4
4	3	5	5	2	2	4	5	4
4	4	5	5	2	2	5	4	4
4	4	5	5	3	2	4	999	4
4	5	4	5	3	3	4	4	3
3	4	3	4	4	2	3	3	4
3	2	3	4	2	3	3	5	4
3	5	5	5	3	2	4	5	5
3	3	5	3	3	3	5	4	3
5	4	5	5	2	1	5	5	5
3	5	5	4	1	4	5	3	5
3	4	5	3	3	1	4	4	3
5	5	5	3	1	2	1	5	5
4	2	4	2	3	2	3	5	5
1	2	2	2	4	3	2	2	1
4	4	3	5	4	2	3	4	5
3	2	5	3	2	3	4	3	3
4	3	5	4	2	2	5	5	4
2	2	4	5	4	3	2	4	4

3	4	5	5	1	3	5	5	5
4	4	5	3	2	2	4	5	5
4	4	5	3	3	2	5	4	1
2	2	4	3	3	3	3	3	4
4	4	4	4	2	2	4	4	4
4	4	4	4	2	3	5	4	5
4	4	5	4	1	1	5	4	5
5	5	4	2	3	1	5	4	4
4	4	5	5	4	2	4	5	4
3	2	5	4	4	4	3	4	4
4	4	5	4	3	1	4	5	5
4	4	5	4	3	2	4	4	4
5	5	5	5	1	1	5	5	5
4	4	5	4	3	2	4	5	5
4	4	5	4	3	1	5	5	5
5	5	5	5	3	1	4	5	5
4	2	2	2	1	3	3	4	5
3	3	4	5	5	4	4	5	3
5	4	5	5	3	1	5	5	5
1	1	3	3	5	3	3	2	3
4	5	5	5	2	1	4	5	5
5	5	5	4	1	1	4	4	3
4	5	4	5	3	2	4	5	4
4	3	5	2	2	1	5	5	4
4	4	5	3	2	2	4	5	5
4	4	4	5	2	1	4	4	5
4	4	5	4	4	2	5	5	5
4	3	4	5	2	2	5	4	4
4	5	5	3	2	3	4	4	5
3	5	4	3	2	3	5	4	4
4	3	5	3	3	1	4	5	5
5	5	5	5	3	1	5	5	5
4	4	5	3	2	1	5	5	5
4	5	4	5	2	2	4	5	4
4	3	5	3	2	1	4	5	5
3	3	4	2	2	2	5	4	4
4	3	5	5	3	2	5	5	5
3	2	4	4	3	1	4	5	4
4	4	4	4	3	2	4	4	5
4	3	5	3	1	1	4	4	5
4	5	5	1	1	1	5	5	5
4	4	5	3	1	1	4	4	5
3	2	5	2	2	2	3	5	4
4	3	5	3	2	1	4	4	3
3	3	3	2	3	3	3	3	3
3	4	5	4	3	2	3	5	4
4	5	5	5	4	2	5	5	5
3	3	5	4	2	2	4	4	4
3	3	5	3	2	2	5	5	5
5	5	5	3	2	1	5	5	1

5	5	5	4	1	1	4	4	4
4	4	4	4	3	3	4	5	5
4	5	4	5	2	1	4	4	4
4	5	5	5	2	2	5	5	5
4	4	5	5	2	1	5	4	4
4	4	5	4	1	3	5	5	4
5	4	5	2	5	1	5	5	1
4	4	4	4	3	2	4	5	5
5	3	3	4	4	1	5	5	5
4	3	5	4	4	4	3	4	3
4	2	3	5	3	2	4	5	4
5	2	5	5	3	1	5	5	5
3	4	3	5	3	3	4	4	3
4	3	5	4	3	1	3	5	3
4	3	4	5	3	2	3	4	1
2	4	5	3	2	1	4	2	5
4	2	4	3	1	1	4	5	5
5	3	4	2	3	2	5	5	1
4	5	5	4	4	2	4	5	5
5	4	4	5	2	2	3	5	4
4	3	5	5	1	1	4	5	5
4	999	5	2	1	1	2	5	5
4	2	5	2	1	1	4	5	5
4	3	5	3	2	1	3	5	4
3	1	3	999	1	1	2	2	2
4	5	5	3	2	1	4	5	4
4	2	5	5	1	1	5	5	1
3	2	3	3	2	4	999	3	4
2	1	5	1	1	1	999	1	1
5	3	5	5	1	1	5	5	1
4	2	5	4	1	1	3	5	5
3	1	3	2	3	2	3	3	3
3	4	5	5	4	1	3	5	5
4	3	5	1	5	1	4	5	3
5	1	5	3	3	1	5	5	3
4	5	5	5	5	1	5	5	4
3	2	4	3	2	2	4	4	3
3	1	3	3	3	2	3	4	3
4	3	4	3	2	2	3	3	5
2	5	4	5	5	1	5	5	4
4	2	4	5	3	1	4	4	3
5	4	5	4	2	1	4	5	5
5	3	5	4	1	1	4	4	4
4	3	5	2	1	1	4	5	5
4	4	5	5	3	2	4	5	5
5	2	4	1	5	5	3	5	5
5	1	5	5	1	1	5	5	1
3	3	3	3	2	2	3	3	4
5	5	3	4	3	2	3	2	4
3	1	5	5	1	1	4	5	5

4	3	4	4	2	3	4	4	1
4	1	5	4	4	1	4	5	5
2	1	2	2	2	3	3	4	2
2	2	3	5	3	4	3	2	4
3	2	5	4	3	3	3	3	4
3	3	3	4	2	1	3	3	5
1	5	5	5	3	1	5	5	5
5	5	5	5	1	1	5	5	5
5	3	5	3	2	1	5	5	5
4	3	5	3	3	2	4	4	5
4	1	5	2	1	3	2	3	5
5	3	5	4	2	2	4	5	4
4	4	5	5	2	2	4	4	5
5	2	5	1	2	1	4	5	5
4	3	3	5	2	4	5	5	4
5	4	5	4	2	1	5	4	5
4	2	5	2	1	1	3	5	5
4	3	5	4	3	1	4	5	5
5	4	5	2	1	1	5	5	5
5	5	4	4	2	1	4	4	5
4	3	5	5	1	1	4	5	5
5	1	5	5	3	1	4	5	4
2	4	5	5	3	1	5	5	4
4	4	5	2	1	2	4	5	5
3	3	5	5	5	1	2	4	3
5	5	5	1	1	1	5	5	5
5	5	5	5	3	1	4	5	5
5	4	5	5	1	1	5	5	4
4	4	4	4	4	1	3	4	4
4	4	5	2	1	1	5	5	4
5	4	5	3	2	1	5	5	5
5	4	5	3	1	1	5	5	5
5	3	5	4	5	1	4	5	5
4	4	5	4	3	2	4	4	1
999	4	4	3	5	1	4	4	5
4	3	5	4	1	1	5	5	5
2	3	999	999	999	3	4	2	5
4	2	5	3	1	1	5	4	5
2	3	4	3	1	1	4	5	4
5	1	5	1	1	1	4	5	5
5	5	5	5	3	1	5	5	5
3	2	5	4	2	1	5	5	5
4	5	4	3	4	1	1	5	5
2	3	5	4	3	1	4	3	2
4	2	4	3	4	1	4	4	4
4	3	5	3	1	1	5	5	5
3	2	4	5	3	2	3	5	4
4	3	5	4	5	1	4	5	5
4	3	4	5	2	3	3	4	3
5	4	5	5	1	1	3	1	5

1	1	1	1	1	5	1	5	5
4	5	5	3	2	1	4	5	5
3	2	4	1	5	2	4	4	2
5	5	5	4	1	1	5	5	5
4	2	5	3	2	1	4	4	1
3	3	4	4	2	2	3	5	4
3	1	1	3	3	3	1	5	1
5	3	5	3	2	1	4	5	5
4	2	5	4	3	2	5	3	5
4	5	5	4	3	1	3	5	5
4	3	4	3	3	1	5	5	5
5	4	5	5	2	1	5	5	5
3	3	3	3	2	3	4	2	2
5	5	5	4	1	1	5	5	5
5	4	5	4	3	1	4	5	5
4	4	5	1	1	1	5	5	5
4	5	5	4	3	1	5	5	4
3	2	3	2	2	2	4	5	4
4	4	5	4	1	2	3	4	4
4	5	4	4	3	2	4	5	4
3	3	5	4	4	1	3	5	4
2	3	2	4	3	3	4	4	5
4	2	5	4	3	2	4	5	3
5	4	5	5	4	2	3	5	5
3	2	4	5	2	1	3	4	3
4	4	4	4	1	1	4	4	4
4	4	5	4	2	1	4	4	4
4	3	4	5	2	3	4	5	3
3	4	5	3	2	1	4	5	4
3	4	5	3	2	1	3	5	5
999	5	3	4	2	3	4	3	3
5	2	5	4	1	1	5	5	5
4	4	5	3	1	1	5	5	4
4	3	5	5	999	1	5	5	5
4	4	5	4	3	1	4	4	4
4	4	5	3	2	2	5	5	5
4	1	5	4	1	1	5	5	5
4	5	4	3	2	2	4	4	5
3	2	4	5	2	1	3	4	4
3	3	3	4	3	2	3	3	3
4	3	4	2	1	1	4	5	4
5	3	5	4	3	1	4	5	5
3	2	5	1	1	1	4	5	5
5	4	5	3	2	1	5	5	5
5	5	5	5	1	1	5	5	5
2	3	3	5	1	1	3	4	5
2	2	3	2	4	3	2	3	4
5	3	4	3	5	4	2	3	4
4	3	5	3	2	1	4	5	4
4	3	5	5	2	1	5	5	4

4	4	5	4	3	2	4	5	4
3	3	4	4	2	3	4	4	4
5	4	4	5	3	2	4	5	5
3	2	4	3	1	1	4	5	5
3	3	5	3	3	3	4	3	3
5	5	5	5	1	1	5	5	5
3	2	2	3	2	999	3	1	2
4	4	5	2	3	1	3	5	5
4	2	5	3	2	2	4	5	5
2	1	4	2	5	3	4	3	3
5	5	5	5	1	1	5	5	5
5	4	5	5	1	1	5	5	5
4	4	3	2	3	2	3	4	4
4	3	5	4	2	1	4	4	3
3	3	2	2	3	2	3	3	3
3	999	5	3	3	1	999	5	5
3	2	5	3	2	1	2	5	4
4	5	4	3	1	1	5	5	5
4	3	5	999	3	999	3	5	5
3	2	4	4	1	1	5	5	5
5	5	5	5	1	1	5	5	5
4	1	5	3	3	1	3	4	4
3	3	4	2	1	2	4	5	3
5	3	5	1	2	1	3	2	5
4	5	5	3	5	1	5	5	5
3	1	5	3	2	1	4	4	3
4	3	4	2	2	1	4	4	5
4	4	5	5	2	1	4	5	4
4	3	5	4	2	1	5	5	4
4	3	4	5	1	1	4	5	4
4	5	5	3	1	1	5	5	5
5	5	5	2	5	1	5	5	4
4	4	5	4	1	1	5	5	5
3	2	4	3	3	4	2	5	2
4	3	5	4	1	1	4	5	4
4	4	5	4	999	1	5	5	5
999	4	4	5	2	2	4	5	5
5	3	5	4	2	5	4	5	5
3	2	5	2	3	2	2	4	3
4	5	3	2	4	1	3	2	4
3	3	4	3	4	4	3	3	4
4	2	4	5	4	1	4	5	5
4	2	5	3	1	2	4	5	5
5	5	5	1	2	1	5	5	5
5	4	5	4	3	1	4	4	5
4	3	5	4	1	1	3	5	4
5	4	5	4	1	1	4	5	5
4	3	3	3	2	2	3	3	3
4	3	4	3	2	2	3	4	5
2	1	4	5	1	2	4	2	5

4	5	999	4	2	1	5	5	4
4	4	5	4	1	1	4	5	5
5	4	5	5	3	1	5	5	5
3	4	4	3	2	1	4	4	4
3	1	5	3	3	1	4	4	3
4	3	4	5	3	2	5	4	4
4	4	4	4	3	1	5	5	5
3	2	4	5	2	1	3	4	3
2	3	5	4	1	1	4	5	1
4	4	4	5	2	2	5	5	5
4	3	5	3	2	1	5	3	5
5	4	5	3	1	2	5	5	5
5	2	5	3	5	1	5	5	4
2	1	5	4	3	3	3	3	4
5	3	5	4	2	1	4	4	5
4	2	5	4	2	1	3	4	3
3	4	4	4	3	2	4	5	5
3	3	5	3	2	1	4	4	4
3	2	3	3	4	3	3	3	3
3	3	5	5	3	1	4	4	5
5	5	5	5	1	1	5	5	5
5	5	5	5	1	1	5	5	5
5	4	5	3	1	1	5	4	5
3	2	4	3	2	2	3	3	4
4	2	5	4	1	2	3	4	5
4	2	5	4	3	1	4	5	5
4	2	5	3	5	1	5	5	5
3	5	3	2	2	3	3	3	2
4	1	4	3	3	2	4	4	4
4	3	4	4	3	1	4	5	5
4	2	3	2	1	2	3	5	4
3	4	3	3	3	3	4	4	3
4	2	5	4	2	2	4	5	4
4	2	5	4	3	2	4	4	5
3	3	4	4	3	3	4	5	3
5	3	5	4	2	1	5	5	5
3	1	5	2	4	1	4	3	4
4	3	5	4	2	2	4	999	3
5	2	5	3	2	1	5	5	5
4	5	5	4	3	1	4	5	4
4	5	5	3	3	1	5	5	4
4	4	5	3	2	1	4	4	4
4	1	4	5	1	2	3	4	4
5	4	5	3	2	1	4	4	5
3	3	4	4	3	2	4	5	5
5	2	5	3	1	1	5	5	5
5	4	5	5	1	1	4	5	5
3	4	4	4	2	999	4	4	4
3	2	4	2	2	2	5	5	5
3	4	3	5	5	4	4	4	5

4	3	5	4	2	1	4	4	3
5	5	4	4	2	1	4	4	5
2	2	2	4	2	2	4	4	4
4	2	5	2	3	1	4	5	5
5	4	5	3	2	1	5	3	5
4	3	5	3	3	1	5	5	5
4	3	5	3	1	2	4	5	5
5	3	5	4	4	1	5	5	4
5	3	5	2	5	1	5	5	5
4	2	4	5	5	2	5	3	4
5	999	5	4	5	1	3	5	5
4	999	4	1	2	1	4	5	5
4	3	5	2	3	999	4	4	5
4	2	5	4	999	1	4	5	4
4	3	5	4	3	3	5	4	4
4	4	5	4	5	1	4	5	5
5	4	5	3	5	1	4	5	5
4	2	5	4	2	1	2	4	5
3	3	4	4	3	1	4	4	2
4	3	5	4	2	1	5	4	3
2	3	2	5	3	4	2	3	2
4	2	5	4	3	2	4	4	4
4	4	4	2	2	2	4	4	4
4	1	5	4	3	3	4	3	5
4	3	5	4	3	3	3	3	4
4	1	5	2	1	1	5	5	4
4	3	4	4	4	1	5	5	5
4	4	4	3	3	1	4	5	4
5	3	5	4	2	1	5	5	4
5	4	5	3	1	1	5	5	5
4	3	5	3	2	2	3	4	3
5	5	4	4	999	2	5	5	5
4	4	4	2	3	1	4	5	5
3	1	5	3	2	1	4	4	3
4	4	5	4	3	2	3	5	4
4	2	4	3	4	3	4	4	4
3	1	5	4	2	1	3	5	4
4	2	5	3	2	1	4	5	5
2	3	4	3	5	5	3	4	2
3	1	5	5	3	4	5	3	4
5	5	5	5	3	2	4	5	4
5	2	5	5	2	1	4	5	5
4	2	4	2	3	3	2	5	5
5	4	5	4	1	1	5	5	5
5	3	5	3	2	2	4	4	4
5	5	5	4	1	1	5	5	1
4	2	4	4	2	2	4	5	3
5	3	5	4	1	2	4	5	5
4	4	5	4	3	2	5	4	5
4	1	3	3	1	1	3	4	3

4	2	5	4	2	2	3	4	5
4	2	5	4	2	1	4	5	5
4	3	5	4	2	1	4	4	5
5	3	5	5	2	1	5	5	5
4	2	4	4	3	2	3	5	5
4	4	5	5	2	1	5	5	5
4	3	5	4	3	2	4	5	2
4	4	5	4	4	2	5	5	5
1	4	5	4	3	1	5	5	5
3	2	5	3	4	2	3	2	5
3	3	5	2	2	3	4	3	4
4	3	4	3	3	3	4	4	4
4	4	5	4	2	2	4	4	4
5	3	5	2	1	1	5	5	5
3	4	5	4	3	3	4	3	5
5	1	5	5	1	1	5	5	5
4	2	5	3	3	2	4	5	3
4	2	4	3	4	2	3	3	3
4	3	5	5	2	2	5	5	4
2	1	3	3	4	2	4	4	3
4	2	5	2	1	2	3	4	5
4	4	5	5	2	1	4	4	5
3	2	5	5	3	1	3	5	5
4	5	5	5	2	1	3	5	4
4	3	4	3	4	1	4	5	4
3	4	5	3	4	3	999	4	5
999	999	999	999	999	999	999	999	999
3	3	4	4	3	2	3	3	3
4	2	3	3	3	2	3	3	4
4	2	4	2	4	2	4	4	3
5	1	5	5	4	2	5	5	5
4	2	5	1	3	1	5	5	4
3	5	5	4	1	1	3	4	2
3	3	5	3	1	1	4	5	4
4	3	4	2	2	1	5	4	4
3	3	4	4	2	1	4	5	5
4	5	5	3	3	1	5	5	5
4	3	5	3	3	1	4	5	4
5	2	5	4	2	1	4	5	5
5	4	5	999	2	1	4	4	4
4	4	5	4	2	1	4	5	4
3	4	5	5	1	1	4	5	5
4	1	5	2	2	1	4	4	5
4	2	5	3	3	1	4	5	5
4	5	3	4	3	1	3	3	4
4	4	5	3	2	1	5	4	5
4	3	5	2	2	1	5	5	5
2	2	4	4	4	2	4	2	2
5	1	5	4	3	2	4	5	5
4	4	4	3	3	2	4	4	5

4	4	5	4	1	1	4	5	5
4	4	5	4	2	1	5	5	4
4	4	5	3	2	2	4	4	4
4	3	5	2	1	1	4	5	5
4	3	5	4	2	1	5	5	4
5	3	5	4	2	1	5	5	4
4	4	5	3	3	2	4	5	5
4	4	4	5	3	2	4	5	4
4	2	4	4	2	1	2	4	4
5	5	5	1	1	1	4	5	5
5	5	5	4	1	1	5	5	5
4	3	5	4	3	3	3	4	3
5	5	5	3	1	1	4	5	5
4	3	5	5	3	2	4	5	4
4	3	4	3	3	2	4	4	4
4	5	5	2	2	1	5	5	5

IPPA_A15	IPPA_A16	IPPA_A17	IPPA_A18	IPPA_A19	IPPA_A20	IPPA_A21	IPPA_A22	IPPA_A23
5	5	5	1	5	5	5	2	1
4	3	5	1	5	5	5	2	1
4	3	4	1	5	4	4	2	2
5	5	5	1	5	5	5	1	1
4	2	3	2	3	4	3	5	1
5	4	4	1	5	4	5	5	1
4	4	5	1	4	5	5	1	1
5	4	5	3	5	4	4	4	2
3	4	5	1	3	5	5	2	3
5	5	5	1	5	5	5	5	1
999	2	3	5	2	1	3	4	2
5	5	5	1	5	5	5	4	1
4	999	4	2	999	999	999	2	999
5	5	4	2	5	5	4	3	2
5	5	5	1	5	5	5	3	1
3	3	3	1	4	5	2	2	2
5	4	5	1	5	5	5	2	1
4	5	5	2	5	5	5	1	1
2	3	2	2	3	5	3	3	5
4	4	3	4	3	3	3	2	4
5	5	5	1	5	5	5	1	1
5	2	2	5	4	4	4	4	5
5	5	3	1	5	5	4	5	2
3	4	4	2	5	999	4	2	4
4	3	5	2	4	5	5	4	1
4	3	4	1	4	5	5	2	3
5	4	4	2	5	5	4	3	2
5	5	5	1	5	5	4	5	1
2	2	3	1	4	5	4	5	1
4	4	5	3	4	4	5	5	2
5	999	5	2	5	5	5	1	1
999	4	999	2	4	3	999	999	999
5	5	5	3	5	5	5	3	1
5	4	5	1	5	5	5	1	1
5	4	5	3	4	5	4	3	2
2	2	2	2	4	2	2	3	1
2	2	2	2	4	5	4	4	4
4	4	5	2	5	5	5	3	2
4	4	4	3	4	4	4	4	2
5	5	4	2	5	4	4	3	3
4	4	5	1	5	5	4	1	2
5	5	5	2	5	5	5	1	1
2	2	3	2	2	3	2	2	4
3	4	4	1	5	5	5	2	1
5	3	4	2	4	4	4	3	2
2	3	2	1	3	3	2	4	5
5	5	5	1	5	5	4	1	1
4	3	4	2	5	5	5	2	1
3	3	4	1	5	5	4	3	1

4	3	4	2	4	5	4	4	3
5	4	4	2	4	5	4	3	2
5	5	1	999	4	5	4	1	1
4	3	4	1	3	3	3	4	1
5	5	5	1	5	5	5	1	1
5	4	3	2	5	5	5	4	1
3	4	2	4	3	2	5	5	5
4	4	4	1	3	3	4	3	3
5	5	5	4	5	4	5	3	1
4	2	4	4	2	4	4	4	3
5	5	5	3	5	5	5	2	2
5	5	5	2	5	4	5	2	2
3	3	2	3	3	2	2	2	2
4	4	5	3	5	5	4	3	2
5	4	5	3	5	5	5	2	2
4	4	5	1	3	4	5	3	1
4	3	4	2	5	5	4	2	1
4	3	4	2	5	4	5	3	1
4	4	5	1	3	4	4	1	1
5	4	5	1	5	5	5	2	2
5	4	3	3	4	4	5	5	2
4	4	4	1	5	5	5	2	2
4	4	4	2	4	4	4	3	2
1	1	3	3	3	3	4	5	3
4	4	5	2	5	5	4	2	2
4	3	4	2	5	5	5	2	2
5	4	4	1	5	5	5	2	1
4	3	4	4	5	5	4	2	3
4	4	5	2	5	4	5	3	1
4	3	3	4	2	5	4	3	3
4	4	4	2	5	5	4	3	2
3	3	4	1	3	5	4	2	3
5	5	5	1	5	5	5	1	1
1	1	1	1	1	1	1	3	5
3	2	3	2	3	3	3	4	2
5	4	5	1	5	5	999	2	2
5	4	5	1	5	5	5	3	1
5	5	5	1	5	5	5	1	1
5	4	4	1	5	4	4	2	1
5	4	5	1	5	5	5	3	1
4	4	3	1	3	5	5	4	1
3	2	2	999	3	3	3	2	2
5	4	5	2	4	5	4	5	1
4	4	5	2	4	5	5	4	1
999	3	3	1	3	4	4	3	1
3	4	3	2	5	5	5	2	2
5	999	999	1	5	5	4	4	3
5	5	5	1	5	5	5	1	1
4	3	4	1	4	4	5	2	1
4	4	5	2	5	5	5	2	1

5	2	4	1	5	5	5	2	1
5	5	5	1	5	5	5	2	1
5	4	5	2	5	5	5	4	1
4	3	3	1	4	3	3	5	3
5	5	5	2	5	5	5	2	1
5	3	3	1	5	5	5	2	1
5	5	5	2	4	3	5	4	2
5	5	5	1	5	5	5	5	1
1	3	4	3	3	2	2	5	1
5	5	5	1	5	1	5	1	1
4	3	4	2	3	4	4	1	2
4	4	5	3	5	5	5	3	2
5	4	5	1	5	5	4	1	1
5	2	5	2	4	3	5	1	2
5	3	5	1	5	5	5	2	1
4	3	5	2	5	5	5	4	1
4	5	5	3	5	5	4	5	2
4	4	5	1	5	5	5	2	1
3	5	4	2	3	5	4	5	3
4	5	4	3	5	4	4	5	3
4	5	5	2	5	5	5	2	1
3	3	3	2	4	5	4	2	1
5	5	5	2	5	5	5	2	2
4	3	4	1	4	4	4	2	2
5	5	4	1	5	5	5	3	3
5	5	5	1	5	5	5	1	1
4	3	4	1	4	4	4	3	1
5	5	5	2	5	5	5	5	1
4	2	3	2	5	5	4	3	2
5	5	4	2	3	3	4	2	2
3	4	3	3	5	5	4	4	3
5	5	5	2	5	5	5	1	1
4	4	5	1	5	4	5	5	1
4	3	5	2	5	4	3	4	2
5	4	3	2	1	5	3	5	1
3	1	3	2	3	4	4	4	2
3	3	5	2	5	4	4	3	3
5	5	5	3	5	5	5	5	3
4	999	999	3	5	4	5	3	3
5	3	4	4	5	3	3	3	1
5	5	4	1	5	5	5	2	1
4	3	5	2	5	4	4	5	2
4	3	4	2	5	5	5	3	2
3	1	4	1	3	5	4	5	3
4	3	5	1	2	4	4	2	2
5	4	5	1	5	5	5	2	1
5	1	5	3	4	3	3	5	3
5	4	5	2	5	5	5	3	1
4	2	3	2	4	4	4	2	2
3	2	4	2	3	3	3	2	2

4	3	4	999	4	5	5	1	1
4	4	4	1	5	5	5	4	2
4	3	5	1	4	5	5	4	1
5	5	5	1	5	5	5	2	1
2	4	5	1	5	5	5	4	1
4	5	4	2	4	5	5	2	1
3	2	4	3	3	4	3	3	4
5	2	5	2	4	3	5	5	2
5	3	5	2	5	5	4	3	1
5	4	5	1	5	5	4	2	1
3	3	5	2	4	3	4	3	1
5	3	5	2	5	5	5	999	1
3	4	3	2	2	3	3	3	3
3	4	3	2	3	5	3	5	2
4	3	4	2	4	4	3	1	2
4	5	4	2	3	5	4	5	4
3	4	4	3	5	5	4	3	1
4	3	5	3	4	5	5	3	1
4	4	5	3	5	4	5	3	3
4	4	5	1	4	5	4	4	4
3	2	4	2	5	4	5	5	3
5	5	5	3	5	5	5	2	1
5	5	5	1	5	5	5	2	1
4	5	5	1	5	5	5	3	1
5	5	5	1	5	5	5	3	1
5	4	4	2	5	5	5	2	1
5	4	4	2	4	4	5	3	2
5	4	5	1	5	5	5	2	1
4	4	5	3	5	4	5	4	3
5	3	5	1	5	5	5	2	1
5	5	5	2	4	5	5	3	1
4	4	4	1	4	4	5	4	1
5	5	3	1	5	5	3	2	2
3	2	2	2	2	3	3	4	2
3	4	999	3	3	3	4	4	3
3	3	4	2	4	4	4	4	1
5	4	5	2	5	5	4	2	2
4	4	4	2	5	5	5	3	2
5	3	5	1	5	5	5	2	1
4	1	5	2	5	5	5	2	2
4	2	3	3	4	5	5	4	3
4	3	5	1	5	5	5	2	1
4	4	4	2	4	4	5	1	1
5	5	4	3	5	5	5	2	3
4	5	4	1	4	3	5	4	2
3	4	4	1	5	5	5	2	2
5	3	4	2	4	4	4	3	2
3	3	4	2	5	5	5	3	1
4	3	4	2	5	5	4	3	1
4	5	5	2	5	5	5	3	1

4	3	4	1	5	5	4	3	1
5	5	4	1	5	5	5	1	1
4	3	4	3	4	5	4	2	3
5	4	5	1	5	5	5	4	3
5	4	5	1	5	5	5	999	2
3	5	5	2	5	5	4	3	1
4	4	4	2	5	5	5	4	2
5	5	5	1	5	5	4	3	1
5	5	5	1	5	5	5	1	1
5	5	5	2	5	4	5	1	1
3	5	4	3	1	3	3	4	2
5	4	5	3	5	5	5	4	2
4	3	4	2	5	5	4	2	1
5	2	4	2	4	4	3	5	1
5	5	5	1	5	5	5	3	1
5	3	5	999	5	5	5	4	1
5	4	5	1	5	5	5	1	1
4	3	5	2	5	4	5	1	1
5	5	4	2	5	4	5	3	2
2	3	4	1	3	4	4	1	1
5	2	5	5	2	2	4	1	3
4	5	5	1	5	4	5	3	1
4	5	5	1	5	5	5	2	1
3	3	2	3	3	3	3	2	4
3	2	5	2	5	3	5	5	1
5	5	5	1	5	5	5	1	1
4	4	5	2	4	4	5	2	1
4	5	5	3	5	5	4	3	3
5	4	5	1	5	5	5	2	1
5	3	4	2	4	5	4	5	3
3	2	3	2	4	4	5	2	2
5	4	4	2	4	5	4	2	2
5	4	5	2	5	5	5	3	2
3	3	5	2	5	5	4	4	2
4	3	4	2	5	4	4	3	2
5	4	5	1	5	5	5	1	1
2	3	4	2	4	4	5	2	2
3	3	3	3	4	3	3	2	2
4	4	5	2	5	4	4	2	2
4	4	5	3	5	4	5	2	2
3	3	3	2	4	4	4	2	2
4	3	5	2	5	5	5	2	1
5	4	5	1	5	4	5	2	1
4	5	5	2	5	5	5	1	1
4	5	4	1	5	4	4	999	2
3	4	5	2	5	5	999	2	3
5	4	4	2	4	5	5	5	2
5	5	5	2	5	5	3	3	3
4	3	5	2	4	4	5	3	2
999	999	999	999	999	999	999	999	999

3	2	2	4	3	3	3	5	4
2	2	4	3	4	4	2	3	4
4	3	4	2	5	5	5	3	2
4	4	5	2	5	5	5	2	1
2	3	3	4	3	3	3	4	2
4	5	5	2	5	5	4	5	1
4	4	4	1	3	5	4	4	2
5	5	5	1	5	5	5	2	1
4	4	3	2	3	3	3	3	3
3	2	3	4	2	2	4	5	5
4	4	4	2	4	5	5	3	1
3	2	4	1	3	4	4	4	2
3	3	5	3	5	5	5	3	1
4	5	5	3	5	5	5	4	2
5	4	5	1	5	5	5	4	4
5	4	5	2	5	5	5	2	2
4	4	5	1	5	4	5	1	1
4	3	5	2	5	5	5	3	1
1	1	2	3	2	1	1	3	2
3	3	4	3	4	4	5	5	5
4	1	2	4	2	2	2	3	4
4	4	5	2	5	5	5	2	1
4	3	3	2	3	3	4	5	3
5	5	5	1	4	5	5	2	1
5	5	5	2	5	5	5	5	1
3	3	3	2	3	4	4	4	3
3	4	5	1	4	4	5	3	2
3	2	3	3	3	3	3	3	3
5	3	5	1	5	5	4	999	1
4	2	4	1	5	4	5	5	1
4	5	5	1	5	4	5	2	999
3	2	3	3	3	3	3	3	3
2	3	5	3	5	4	5	3	2
3	3	5	3	5	4	4	3	2
3	2	4	1	5	5	5	2	3
3	3	4	3	3	3	4	5	2
4	4	4	2	5	4	4	3	1
5	1	5	1	5	5	5	1	1
4	4	5	2	5	5	5	4	1
5	5	5	2	5	5	5	2	1
4	3	5	1	5	5	5	3	1
4	4	5	1	5	5	5	4	1
2	3	4	3	5	4	5	2	2
3	4	5	2	5	4	4	3	2
3	3	3	2	4	4	4	2	1
3	2	4	4	3	4	4	3	2
5	4	5	2	5	5	5	3	2
4	4	5	2	5	5	5	3	1
5	2	5	2	5	5	5	5	1
4	4	4	2	4	4	4	4	2

5	3	5	2	5	5	5	2	2
5	5	5	2	5	5	5	3	2
2	1	2	3	3	3	4	5	3
3	3	3	3	4	4	5	5	2
4	999	5	4	3	3	2	2	4
4	5	5	1	5	5	5	3	1
3	4	5	2	4	5	5	4	2
4	5	4	2	4	5	5	2	1
5	5	4	1	5	4	5	2	1
3	2	5	2	3	4	4	4	2
4	4	4	1	5	5	4	3	2
4	5	5	1	5	5	5	4	1
3	3	5	2	4	4	5	3	2
5	5	5	1	5	5	5	4	1
5	5	2	5	5	5	5	4	1
5	5	5	3	4	5	5	4	1
4	5	5	1	5	5	5	4	1
4	3	5	1	5	5	5	4	1
3	3	4	2	5	5	5	2	2
4	3	4	2	4	4	4	2	1
3	2	5	2	5	5	5	2	2
5	5	5	1	4	5	5	2	1
4	5	5	1	5	5	5	2	2
3	2	3	2	3	3	999	3	3
3	4	5	1	5	5	5	3	2
4	4	5	1	4	5	5	2	2
3	3	2	3	4	3	2	5	4
4	2	4	2	5	4	4	4	2
4	4	5	2	5	5	4	4	2
5	4	5	1	5	4	5	2	1
3	3	3	3	3	4	3	3	2
4	4	5	3	4	4	4	3	2
5	5	5	3	5	5	4	4	2
4	4	4	1	4	4	4	3	1
3	4	5	2	5	5	5	4	1
4	3	4	2	4	4	4	3	2
4	5	5	1	4	5	5	2	1
4	4	4	1	4	4	4	4	2
4	4	4	2	4	4	4	2	1
5	5	5	2	5	5	5	2	1
4	4	5	2	5	5	5	3	1
4	5	5	2	5	4	5	2	1
4	4	4	2	4	3	4	3	2
4	3	5	1	4	3	4	2	1
4	4	5	2	5	5	5	1	1
4	4	5	2	5	5	5	1	1
5	5	5	1	5	5	5	2	1
5	4	5	2	4	5	5	3	2
5	4	3	1	4	3	4	5	3
5	4	5	2	5	5	5	2	2

4	4	4	1	5	5	5	3	3
2	2	2	3	2	4	3	4	2
3	1	3	2	4	4	5	2	2
4	3	4	2	4	4	3	2	3
3	3	3	2	5	5	4	2	3
4	3	5	2	4	4	4	3	2
4	4	5	2	4	5	5	3	2
5	5	5	1	5	5	5	2	1
4	5	4	2	5	4	4	2	2
5	2	4	2	5	4	5	3	1
5	5	5	1	5	5	5	2	1
4	5	5	1	4	5	4	2	2
4	5	5	1	5	4	4	2	2
4	4	4	2	2	5	4	1	2
3	3	4	2	4	4	3	2	2
4	3	5	2	5	5	5	2	1
4	3	5	1	5	5	5	4	1
5	4	5	1	5	5	5	2	1
5	1	5	2	5	5	5	3	1
4	4	999	3	4	5	4	2	2
3	4	4	2	4	4	5	2	3
4	3	5	2	5	5	4	4	2
3	4	4	3	4	3	4	2	2
5	5	5	1	5	5	5	2	1
5	4	5	2	5	5	4	4	1
4	3	4	2	4	4	3	2	2
4	4	5	2	5	5	5	3	1
4	3	4	2	5	5	4	4	2
3	2	3	2	4	4	3	4	2
4	5	5	2	4	5	4	1	1
5	4	5	3	4	5	4	4	3
2	2	3	4	3	3	3	5	2
3	4	3	2	5	5	4	2	2
5	4	4	2	5	5	4	4	1
3	3	5	2	5	5	5	3	1
3	3	4	2	4	4	4	4	2
4	3	3	2	4	4	4	4	999
5	3	4	1	3	4	4	4	3
5	4	5	2	5	4	4	3	2
3	3	4	2	3	4	5	5	3
5	5	5	1	5	5	5	3	2
4	3	3	2	2	2	3	4	1
3	4	4	2	5	4	4	3	2
5	4	5	1	4	5	4	3	1
3	2	4	2	3	4	4	5	3
1	1	2	2	1	2	3	5	4
4	2	5	3	4	3	2	5	1
4	3	4	2	3	4	5	2	1
5	4	5	2	4	5	5	2	2
3	2	3	1	3	4	4	5	2

3	5	5	2	5	5	5	3	1
2	4	5	5	2	4	5	5	2
5	4	5	1	5	5	5	2	1
3	4	3	3	3	4	3	3	3
5	5	5	2	4	4	5	3	2
4	5	4	3	5	4	5	4	2
5	5	5	1	5	5	5	3	1
4	4	5	2	5	5	4	3	2
4	5	5	2	5	4	5	5	2
4	2	3	2	4	5	5	5	1
5	4	5	1	5	5	4	2	1
4	3	4	2	4	4	4	3	2
5	5	5	2	5	5	5	3	1
5	4	4	2	5	5	5	4	2
5	3	5	3	4	5	5	5	2
5	5	5	2	5	5	5	2	2
4	4	4	1	4	5	5	999	2
2	2	3	3	3	5	5	4	5
5	5	5	1	5	5	5	4	2
2	1	2	4	2	2	2	5	3
5	4	5	2	5	5	5	3	2
4	5	5	2	4	5	5	3	1
4	3	3	2	4	5	5	5	2
4	4	5	1	4	5	5	2	2
4	4	5	2	4	5	5	3	2
4	5	5	2	4	5	5	2	2
4	4	5	2	5	5	5	4	2
4	4	4	2	4	4	4	2	1
4	3	5	1	3	4	3	3	2
5	4	3	2	4	5	5	3	1
5	3	4	1	5	999	5	3	1
5	4	5	1	4	5	5	4	3
5	5	5	1	5	5	5	2	1
4	5	5	2	5	5	4	3	1
3	4	5	2	5	5	4	4	1
4	4	5	2	5	4	5	2	1
5	4	5	2	5	4	5	4	2
4	4	4	3	5	4	4	4	2
5	4	4	2	4	4	5	3	2
3	3	4	3	5	4	5	1	1
5	5	5	3	5	5	5	3	1
4	1	4	2	5	4	5	1	2
3	3	3	2	4	5	3	4	2
4	4	4	2	4	3	4	2	2
3	3	3	3	3	3	3	4	3
4	3	4	2	4	4	4	4	2
5	3	3	1	5	5	5	4	3
4	4	4	2	4	4	4	4	1
3	3	5	2	5	5	5	2	2
5	5	5	1	5	5	5	2	1

5	4	5	2	5	4	4	2	1
5	5	4	2	4	5	5	2	2
4	5	5	3	5	4	4	4	3
5	4	5	2	5	5	5	2	2
4	3	5	2	4	5	5	5	2
5	4	5	2	5	5	5	1	3
5	5	5	2	5	5	5	2	2
4	3	4	2	4	4	4	3	2
4	4	5	1	5	4	5	4	1
4	3	4	1	3	4	3	5	3
5	4	5	2	5	4	5	2	2
4	1	5	1	5	5	5	2	1
3	4	4	2	4	3	4	4	3
3	4	4	1	3	4	4	3	1
4	5	3	2	4	3	2	4	3
5	4	4	1	3	5	999	4	3
4	4	4	2	4	5	4	1	1
4	4	4	1	4	3	4	2	2
5	4	4	2	5	4	5	2	1
5	4	3	1	5	4	3	4	2
5	5	5	2	5	4	4	5	2
5	4	4	1	5	5	999	4	1
4	4	4	1	3	5	5	5	1
4	4	4	2	4	5	5	4	2
2	2	1	2	1	2	2	2	999
4	4	4	2	5	4	4	2	1
2	3	3	2	2	5	4	3	2
3	2	4	1	2	3	2	3	3
2	1	2	2	2	2	2	2	2
5	4	5	1	5	5	5	3	1
4	2	4	2	4	5	4	1	1
2	1	2	3	2	2	2	4	3
4	3	5	2	5	5	4	3	1
4	5	3	2	4	2	1	5	2
5	3	5	3	1	4	3	3	2
5	4	5	4	5	5	5	3	2
3	4	3	2	3	4	4	4	2
2	1	3	3	4	4	4	3	3
4	1	4	999	4	4	5	2	2
4	1	4	4	3	2	3	5	1
5	2	4	3	5	4	5	2	2
3	3	3	1	4	5	5	3	2
4	4	5	1	5	5	5	2	1
3	2	3	4	2	5	5	3	1
5	4	5	1	4	5	5	2	2
2	3	5	1	1	5	3	1	2
5	1	5	1	4	3	5	1	5
4	3	3	2	3	4	3	3	3
2	3	4	5	5	4	2	3	4
4	3	5	1	4	5	5	1	1

3	4	3	2	3	4	3	2	2
4	2	3	2	3	4	4	4	1
3	1	2	3	3	4	4	4	3
3	2	2	3	2	3	3	4	3
3	2	3	2	4	4	3	4	2
1	3	3	3	3	4	3	3	3
5	1	5	3	5	5	1	2	1
5	1	5	2	4	5	5	1	2
4	4	5	2	5	5	4	2	1
2	3	4	3	4	5	5	2	1
3	2	1	3	4	2	5	5	3
5	3	5	2	4	5	5	3	1
3	4	5	2	5	5	4	4	1
5	2	4	3	5	3	4	2	2
3	5	4	999	2	5	5	4	3
5	4	5	1	5	5	5	2	1
4	2	2	2	4	4	5	3	1
4	3	4	1	5	4	5	2	1
5	5	5	1	5	5	5	1	1
4	4	4	3	5	4	5	4	1
4	4	4	2	5	5	5	1	1
5	1	4	2	5	5	1	2	1
5	3	5	2	4	5	999	1	1
4	3	4	2	4	4	5	2	1
2	5	4	1	3	4	3	5	1
5	5	5	1	5	5	5	1	1
5	5	5	1	5	5	5	2	1
5	4	5	1	5	5	4	3	2
4	4	4	3	5	5	5	3	1
4	4	4	1	4	5	5	1	1
5	5	5	2	5	5	5	3	2
5	5	5	1	5	5	5	4	4
4	3	5	1	5	5	5	3	1
5	4	4	2	5	5	5	4	2
1	1	3	1	4	999	4	5	1
4	4	4	1	3	5	5	2	1
3	3	999	3	4	4	3	5	4
2	2	3	3	2	3	5	2	1
4	3	4	3	3	3	3	1	2
1	1	3	1	1	5	2	1	1
5	5	1	5	5	5	5	1	1
5	4	5	2	5	5	3	2	1
4	3	4	2	5	5	4	2	2
1	3	4	2	5	4	4	3	3
2	2	3	2	3	4	3	4	1
5	4	5	2	5	5	4	2	1
3	2	3	2	4	4	4	2	2
4	4	5	1	5	5	5	3	1
3	2	5	3	4	4	5	4	3
5	5	5	5	4	5	4	1	1

1	1	1	4	1	4	1	1	3
5	5	4	1	5	5	5	4	2
4	2	5	3	3	2	3	4	3
5	5	5	2	5	5	5	4	2
4	3	4	2	1	5	4	4	1
4	3	4	1	4	4	4	3	1
3	1	1	5	1	3	3	3	3
5	4	5	1	5	5	5	1	2
3	2	4	3	3	4	4	5	2
4	4	5	2	4	5	4	5	1
4	4	3	2	3	4	4	3	2
5	4	5	1	5	5	4	1	1
2	3	2	2	2	3	3	3	3
5	5	4	1	5	5	5	3	1
5	4	5	1	5	5	5	2	1
5	5	5	2	5	5	5	1	1
5	4	4	2	4	5	5	4	2
3	3	3	2	3	4	4	3	2
4	4	4	2	4	5	4	1	2
4	5	4	1	4	4	5	5	2
4	3	3	1	3	5	4	3	2
3	4	5	3	5	1	1	4	1
4	4	3	2	4	5	3	2	2
3	999	4	1	5	5	4	4	1
3	2	3	3	4	3	3	3	4
4	4	4	1	4	4	4	4	1
4	3	5	2	4	5	4	3	2
5	3	4	2	3	5	5	2	2
4	4	4	3	5	3	4	2	2
5	3	4	3	4	5	3	5	2
4	3	5	2	5	3	3	2	1
5	4	3	1	4	5	5	3	1
3	3	5	2	4	5	4	2	1
4	3	4	3	4	4	5	1	1
3	3	4	1	4	5	5	4	2
4	4	5	2	5	5	5	4	2
5	4	5	2	4	5	5	2	2
5	4	4	2	4	4	5	3	2
2	3	4	1	4	4	4	3	1
2	3	3	2	2	3	3	3	3
4	4	4	2	4	4	4	2	2
3	4	4	2	4	5	4	3	2
5	3	5	1	4	5	5	1	1
4	4	5	1	4	4	3	2	1
5	5	5	3	5	5	5	2	1
5	4	4	1	3	3	2	4	3
3	2	3	4	2	3	2	3	4
2	4	5	2	4	3	2	4	4
4	4	3	1	5	5	4	3	2
3	4	4	1	3	4	3	2	1

3	4	4	2	5	5	4	5	2
4	4	4	999	5	4	4	1	1
4	4	4	2	4	5	4	3	3
5	3	4	2	3	5	3	3	3
1	3	3	3	3	3	4	4	3
5	5	5	5	5	5	5	4	4
3	2	4	3	1	2	3	2	1
4	4	2	2	4	5	3	1	1
5	4	4	2	3	4	4	3	1
4	3	3	2	4	5	3	5	2
5	4	4	1	5	4	5	3	1
5	5	5	1	5	5	5	1	1
3	3	3	2	3	4	3	2	2
4	4	4	2	3	4	4	2	1
2	2	3	3	3	3	3	3	3
4	3	5	2	5	5	5	1	1
3	2	4	2	3	5	4	2	1
5	4	5	1	4	5	4	3	2
4	4	4	2	2	5	999	999	999
3	3	3	1	5	5	5	1	1
5	5	5	2	5	5	5	5	1
3	2	3	2	3	4	4	3	2
3	2	999	2	2	3	4	2	3
4	2	4	3	2	3	2	4	1
5	3	5	2	5	5	5	2	1
2	3	3	3	2	4	3	2	2
4	2	5	2	5	4	5	3	2
3	3	4	2	4	4	5	2	1
4	2	5	2	4	5	4	2	2
4	4	5	1	4	5	4	2	1
5	4	2	5	5	5	5	2	1
5	5	5	2	4	5	4	2	1
4	3	5	2	5	5	4	2	1
5	3	4	2	5	5	5	2	1
5	3	4	1	5	5	5	2	1
5	4	5	1	5	5	4	5	1
4	5	5	2	5	5	3	3	4
4	4	5	1	5	5	5	2	1
3	2	3	1	3	5	4	2	1
5	3	4	1	2	4	2	3	1
3	2	2	3	2	4	4	3	4
4	4	4	2	4	4	3	3	3
4	4	4	2	5	4	4	1	1
5	5	5	3	5	5	5	3	1
5	3	5	2	4	5	5	3	1
4	4	3	1	3	4	4	3	1
5	4	5	2	4	5	5	3	2
2	2	3	3	2	3	2	3	2
4	4	4	2	3	4	4	3	2
4	1	3	2	2	4	3	2	1

2	3	4	2	2	4	4	3	2
4	4	4	1	4	5	4	3	1
4	5	5	1	5	5	5	2	1
3	4	4	2	5	5	5	3	2
4	2	3	2	3	4	4	3	2
3	3	4	2	4	4	4	2	2
4	5	5	1	4	5	5	5	1
3	2	3	2	4	4	4	1	2
5	5	4	2	5	4	5	1	1
3	2	5	2	5	5	4	3	4
4	4	4	3	4	3	4	4	2
5	4	5	1	5	5	5	1	1
4	2	2	1	3	3	3	2	1
4	3	3	2	3	4	4	3	3
4	4	4	2	4	5	4	3	1
5	3	4	2	3	4	4	3	2
4	4	4	2	4	4	4	4	2
4	4	4	2	4	5	4	2	1
4	3	3	2	4	4	3	3	3
5	3	5	2	5	5	4	3	1
5	5	5	1	5	5	5	1	1
5	5	5	1	5	5	5	2	1
3	5	4	2	3	3	4	4	1
4	3	4	2	4	3	4	3	2
4	4	3	2	4	4	4	2	2
3	2	4	2	5	4	4	3	1
4	3	4	2	5	5	5	2	2
4	4	2	3	999	2	3	2	3
3	1	4	1	5	5	4	4	1
4	3	4	2	4	4	4	4	3
2	2	3	2	3	4	3	3	3
3	4	4	2	4	3	4	3	3
3	3	4	1	4	5	5	4	1
4	3	4	2	4	4	4	2	2
4	3	3	2	3	4	4	3	2
4	5	5	1	5	5	5	2	1
2	4	3	2	2	5	3	5	1
3	2	3	2	3	4	4	3	3
5	4	4	2	5	4	5	3	1
5	3	4	1	5	5	5	5	1
4	3	4	3	4	5	5	4	2
5	4	4	2	4	4	4	4	2
1	2	2	2	5	4	2	1	2
4	3	4	2	5	999	4	1	1
4	3	4	1	4	4	4	4	1
4	4	5	1	3	5	4	3	1
5	5	4	1	3	5	5	4	2
4	3	3	2	4	4	4	3	999
4	2	4	3	3	4	4	3	2
3	3	4	2	3	4	2	3	3

4	3	4	3	5	3	4	2	1
5	5	4	2	4	5	5	3	1
3	3	4	1	4	5	4	4	1
5	1	5	2	4	4	4	3	2
5	3	4	2	5	4	4	2	2
4	3	4	2	4	4	4	3	2
4	3	4	2	3	5	4	2	2
4	5	5	2	4	4	5	4	2
4	3	4	2	3	5	5	2	3
4	4	3	2	4	4	3	2	2
2	2	4	2	5	5	5	3	2
4	999	4	2	4	5	4	2	2
3	3	999	2	3	4	3	4	1
4	4	4	1	4	4	4	1	1
2	3	4	2	4	4	5	3	3
4	3	4	2	4	4	4	3	2
4	3	3	999	4	5	3	2	3
2	2	2	2	3	4	3	2	2
3	3	2	3	4	4	4	3	1
2	3	3	3	5	4	4	2	1
3	2	2	3	3	3	1	4	2
3	3	4	2	4	4	3	2	999
4	3	4	3	4	4	4	3	3
3	2	2	3	5	4	5	5	3
3	4	5	2	5	5	5	2	2
4	4	4	2	5	5	5	2	1
4	4	4	2	4	5	4	2	2
4	3	4	3	5	5	4	3	1
4	3	4	2	4	4	3	2	1
5	4	5	2	5	5	5	2	1
5	4	5	1	4	4	5	999	2
4	4	5	2	5	5	4	1	5
4	4	4	1	4	5	4	5	1
3	3	4	3	5	4	4	3	1
4	3	4	2	4	5	4	3	2
4	3	5	2	999	5	4	2	5
3	1	2	2	4	5	4	4	2
4	4	4	2	4	5	4	2	2
1	1	1	5	3	1	2	5	5
5	3	4	4	3	3	5	5	4
4	1	4	2	4	5	5	5	1
3	3	3	1	4	5	5	2	2
5	1	3	2	4	3	2	1	4
5	3	5	1	5	5	5	1	1
3	4	4	2	4	4	4	3	2
5	5	5	1	5	5	5	4	4
4	3	4	5	5	5	5	1	5
5	4	3	2	4	3	3	3	3
4	3	5	2	5	5	5	3	2
3	3	3	1	2	5	4	5	3

2	1	4	2	3	4	4	1	2
5	4	4	2	5	5	5	2	2
3	4	4	2	4	4	3	3	2
5	1	5	1	5	4	5	5	1
3	2	3	2	4	5	4	3	1
4	4	4	2	4	4	5	3	2
4	3	5	2	4	4	4	2	2
4	4	5	1	5	5	5	5	1
4	4	4	2	4	4	3	3	2
3	2	3	3	4	3	2	4	2
5	3	3	3	3	3	4	5	4
4	3	5	2	4	4	4	3	2
4	3	4	2	5	5	5	2	2
4	5	5	1	5	5	5	4	1
4	3	4	3	3	4	4	4	2
5	1	5	1	5	5	5	1	1
3	1	3	3	4	5	3	3	1
3	2	4	2	4	3	3	4	2
3	4	4	2	5	5	3	3	1
3	2	4	2	3	4	3	5	4
3	2	2	3	2	4	2	3	3
4	4	3	2	4	4	5	3	2
3	2	3	2	3	4	4	4	2
5	3	5	1	4	5	4	3	1
3	3	3	2	4	4	3	2	2
4	3	4	3	4	4	4	4	2
999	3	3	3	3	4	4	2	3
3	2	4	3	3	5	4	3	2
4	3	4	2	3	3	3	3	2
3	2	2	1	5	5	5	5	2
4	3	3	1	2	4	5	4	1
4	1	4	2	5	5	5	5	1
4	1	5	3	5	5	5	2	2
3	2	3	1	3	5	3	2	1
3	5	5	3	4	5	4	2	2
3	3	4	1	4	4	2	3	1
5	5	5	2	5	5	5	3	1
4	3	3	1	4	5	4	2	1
5	3	4	1	3	5	5	4	2
4	4	4	2	4	5	4	3	2
3	3	4	2	4	5	4	2	999
5	5	4	1	5	5	5	2	2
5	3	4	1	5	5	4	2	1
4	2	5	1	5	5	5	4	2
4	4	5	3	3	3	4	5	4
5	4	5	2	5	5	5	2	1
5	4	5	2	5	5	5	1	1
2	2	4	3	3	2	2	4	2
4	1	4	1	4	5	5	2	1
4	3	4	2	3	4	4	4	3

4	4	5	2	5	5	5	1	1
4	5	4	1	5	4	5	1	2
4	3	3	2	4	4	4	2	1
4	4	5	2	5	5	5	2	1
5	4	5	2	5	5	5	3	2
4	5	5	2	5	5	5	1	1
5	4	5	2	4	5	5	2	1
4	4	5	3	4	4	4	4	2
1	1	3	2	3	2	2	1	1
3	2	4	1	3	4	5	1	1
5	5	5	1	5	5	5	1	1
3	2	4	2	3	3	4	3	3
5	4	5	1	5	5	4	3	2
4	4	4	3	5	5	5	5	2
4	3	4	2	4	4	4	3	2
5	4	5	2	5	5	5	3	2

IPPA_A24	IPPA_A25	SDQ1	SDQ2	SDQ3	SDQ4	SDQ5	SDQ6	SDQ7
4	5	3	1	1	3	1	1	3
4	4	3	2	1	3	2	3	2
4	4	3	2	3	2	3	1	2
5	5	3	2	1	3	2	1	3
2	3	2	2	2	3	2	2	2
4	5	3	2	3	3	1	1	2
2	4	3	2	3	3	1	1	3
4	5	3	2	3	3	3	2	2
4	5	3	2	3	3	2	1	2
5	5	3	1	3	3	1	1	3
2	3	2	3	2	2	2	1	2
5	5	3	1	1	3	2	2	1
999	5	999	1	1	2	2	2	2
5	5	3	2	1	3	1	1	3
5	5	3	1	1	3	1	1	3
4	5	2	1	1	3	1	1	3
4	4	3	2	1	3	1	1	3
4	3	3	1	1	3	2	1	3
1	2	2	3	2	2	1	1	2
3	4	3	1	2	3	1	1	3
4	5	3	1	3	3	1	1	2
2	4	3	1	3	3	3	2	2
5	4	3	1	2	3	1	1	3
5	4	2	3	2	2	3	1	2
4	5	3	2	1	3	1	1	3
4	3	3	1	2	3	1	2	3
4	5	2	1	2	2	2	1	2
3	5	3	2	3	3	2	1	3
2	3	2	3	2	3	3	1	1
5	5	3	3	2	3	3	1	2
5	5	3	2	2	3	1	1	2
3	4	999	999	999	2	2	2	999
3	5	2	3	2	2	3	2	2
4	5	2	3	1	2	1	1	2
3	3	3	1	1	3	1	2	2
1	2	2	3	2	2	2	2	1
3	3	3	1	2	3	1	1	3
5	5	3	1	1	3	1	1	3
4	5	2	1	2	2	1	1	2
4	5	2	1	1	3	1	1	2
5	5	3	1	1	3	1	1	3
5	5	3	2	1	3	1	1	3
3	4	3	2	2	3	1	2	3
2	4	3	1	1	3	1	1	3
3	5	3	2	1	3	1	1	2
2	2	3	3	2	2	3	3	3
5	5	3	1	1	3	1	1	2
3	4	3	2	1	3	1	1	3
4	5	2	1	1	2	2	1	2

5	3	2	1	1	3	1	1	1
5	5	3	2	2	3	3	1	2
4	4	3	1	1	3	1	1	3
4	4	2	1	1	2	1	1	2
5	5	3	2	1	2	1	1	3
3	5	2	1	1	3	1	1	2
5	3	3	3	1	3	3	3	3
4	4	3	3	2	3	2	1	2
4	5	3	2	2	2	1	2	2
4	4	3	1	1	2	1	1	2
5	5	3	2	2	3	2	1	2
5	5	3	2	2	3	1	1	2
1	1	3	1	2	3	1	2	3
5	4	3	1	2	3	1	1	3
5	4	3	3	1	3	3	1	2
1	4	3	2	2	3	2	1	2
4	4	3	2	1	3	1	1	3
4	4	3	1	1	3	1	1	3
3	5	3	2	1	3	1	1	3
3	5	3	2	2	3	1	1	3
5	5	3	2	1	3	2	1	3
5	5	3	2	1	3	1	1	3
4	3	3	2	2	2	1	2	2
1	2	2	1	1	3	3	1	3
4	5	3	2	1	3	1	1	3
4	4	3	1	1	3	1	1	3
4	4	3	3	1	3	2	1	3
5	4	3	2	1	3	2	2	3
4	5	3	2	1	3	2	1	3
4	4	3	1	1	3	1	1	2
5	5	2	1	1	2	2	1	3
5	4	3	2	1	3	3	1	1
5	5	3	2	1	3	2	1	3
4	1	3	1	1	3	1	1	3
2	4	3	1	2	3	1	1	1
5	5	2	3	3	2	3	999	3
5	5	3	3	1	2	2	1	2
4	5	3	1	1	3	1	1	3
4	4	2	3	1	3	2	1	1
4	5	3	3	1	3	2	1	3
5	4	3	1	2	3	3	1	3
3	3	2	2	1	2	3	1	2
3	4	3	2	1	3	2	1	999
4	5	3	2	1	3	2	1	3
2	3	3	2	1	3	1	1	2
3	4	2	3	1	2	1	1	2
4	5	2	2	2	2	1	1	2
5	5	3	1	1	3	1	1	3
4	4	3	1	1	3	1	2	3
4	4	3	1	1	3	2	1	3

4	5	2	3	1	3	3	1	1
5	5	3	1	1	1	1	1	3
5	5	3	2	1	3	1	1	3
2	2	3	1	1	2	3	1	2
5	5	3	1	1	3	2	1	3
3	5	3	1	1	3	1	1	2
4	5	2	1	2	2	2	1	2
5	5	3	1	1	3	1	1	3
2	4	3	1	2	2	1	2	2
5	5	3	1	1	3	1	1	3
3	2	3	1	2	2	2	1	2
4	4	3	2	2	3	2	1	3
5	5	2	3	2	3	3	1	3
2	5	3	1	1	3	2	1	2
4	5	3	2	1	3	1	1	3
4	4	3	2	1	3	1	3	2
5	5	2	1	1	3	1	1	3
5	4	3	3	1	3	2	1	2
4	3	3	1	2	3	1	1	3
3	5	3	3	1	3	2	1	3
5	5	3	1	1	3	2	1	2
3	2	999	1	1	2	1	1	2
4	5	2	1	1	3	2	1	2
2	4	2	2	1	2	2	1	2
3	5	3	2	2	3	3	1	3
5	5	3	1	1	3	1	1	2
3	3	3	3	2	3	2	999	2
5	5	3	2	1	3	2	1	3
5	5	3	1	1	3	2	999	999
4	3	3	2	3	1	2	3	3
5	5	3	2	1	2	2	999	2
5	5	3	2	3	3	1	1	3
2	4	2	3	3	3	3	1	2
5	5	3	3	3	2	2	2	3
5	2	2	3	2	3	3	1	2
3	4	2	2	1	3	2	1	2
4	4	3	1	1	3	2	1	3
4	5	3	3	2	2	3	2	2
4	5	2	999	1	2	2	1	2
4	4	3	1	2	2	2	1	1
5	5	3	2	1	1	2	1	2
2	5	3	2	2	3	3	2	2
5	5	3	3	3	3	2	1	2
2	3	3	1	2	3	1	2	2
4	5	2	1	2	3	1	1	2
5	5	3	1	2	3	1	1	3
3	5	2	3	3	3	3	1	1
3	5	3	2	1	3	1	1	3
4	3	2	1	1	3	3	2	2
4	4	3	3	3	3	2	999	2

3	4	2	3	1	2	3	2	2
4	5	3	1	1	3	1	1	2
3	4	3	2	1	3	1	1	3
5	5	3	2	2	3	2	1	2
5	4	2	3	1	3	3	1	2
3	4	3	1	2	3	2	1	3
4	4	3	1	2	2	1	999	999
3	5	3	1	1	2	1	1	3
4	5	3	1	1	3	1	999	999
5	5	3	2	3	3	2	1	2
3	5	2	3	2	3	2	2	2
5	5	3	1	1	3	1	1	3
2	1	3	1	3	3	2	2	3
4	3	3	1	1	3	2	1	2
3	4	2	1	1	3	1	1	3
3	5	3	3	1	3	2	1	3
3	3	3	1	1	2	2	1	2
4	5	3	1	2	3	1	2	2
4	5	2	1	1	2	3	1	2
5	5	3	2	1	3	1	1	2
4	4	3	1	2	2	2	1	2
5	5	3	1	1	3	3	1	2
5	5	3	2	1	3	1	1	1
5	5	3	3	2	3	2	1	2
5	5	3	1	1	2	2	1	2
4	4	3	1	1	2	2	1	3
2	3	3	1	1	3	1	1	3
5	5	3	2	1	3	1	1	3
4	5	3	2	1	3	2	1	2
5	5	3	2	1	3	1	1	3
5	5	3	2	2	3	2	1	2
5	5	3	1	2	3	1	1	3
5	5	3	2	2	3	2	1	3
2	2	2	1	1	2	1	2	3
3	3	3	2	1	2	2	1	2
5	4	2	2	1	2	1	2	2
5	4	3	2	1	1	2	1	2
5	4	2	3	1	3	2	1	3
5	5	3	1	1	999	1	1	3
5	5	3	2	1	3	1	1	3
4	3	3	2	3	3	2	3	2
5	5	3	1	1	3	1	1	3
4	5	3	2	1	3	1	1	3
5	4	3	3	2	999	1	1	2
4	4	2	3	1	2	1	1	3
4	4	3	1	1	1	2	999	3
3	4	2	3	1	2	2	2	2
4	5	3	1	1	3	1	1	3
3	5	3	3	1	3	1	1	3
5	5	3	2	2	3	1	1	3

3	4	3	3	1	2	3	1	2
5	4	3	1	3	2	2	1	3
3	4	3	2	2	3	1	1	2
5	5	2	3	2	2	1	1	2
5	5	3	2	1	2	1	1	2
5	3	2	3	3	3	3	1	1
3	4	3	2	1	2	1	1	2
5	5	2	2	1	3	2	3	1
5	5	3	3	999	3	1	1	3
5	5	3	2	1	3	1	1	2
2	2	2	1	1	3	3	2	1
999	5	3	2	1	3	1	1	3
3	5	3	1	1	3	2	1	3
3	5	3	2	1	3	2	1	3
5	5	3	2	1	2	3	1	3
3	5	3	2	1	3	1	1	2
5	5	3	1	1	3	1	1	2
3	4	3	1	1	2	1	1	3
5	5	3	2	1	3	1	1	3
3	2	3	1	1	2	1	1	3
1	5	3	1	1	3	2	3	3
4	5	3	3	1	3	2	1	2
5	5	3	1	1	3	1	1	3
3	3	2	2	1	2	2	3	3
1	3	3	3	2	3	3	1	2
5	5	3	1	1	3	1	1	3
4	5	3	2	1	2	1	1	3
5	5	3	1	2	3	2	1	3
5	5	2	1	1	2	3	1	2
2	5	3	2	1	3	1	1	3
3	5	3	1	1	3	1	1	3
4	4	3	2	1	3	2	1	3
5	4	3	2	1	2	2	1	3
5	4	3	3	1	3	2	1	2
4	4	3	2	1	3	3	1	2
4	5	3	3	1	3	1	1	3
5	3	2	3	1	3	1	1	3
3	4	2	3	1	2	2	1	3
5	4	2	3	1	3	2	1	2
4	5	2	3	2	3	1	1	3
4	3	3	1	1	2	1	1	2
4	4	3	2	1	3	1	1	2
3	5	3	1	2	3	1	1	3
5	5	3	1	1	3	1	1	3
3	4	3	1	1	3	1	1	2
4	5	3	2	1	3	2	1	2
3	4	2	1	1	3	2	1	3
4	4	3	1	2	2	2	1	2
3	3	3	2	1	2	2	2	3
999	999	999	999	999	999	999	999	999

2	4	3	1	1	3	2	3	2
3	3	3	2	3	2	3	2	2
3	4	3	1	2	3	1	2	2
5	5	3	3	2	3	2	1	3
3	3	2	3	3	3	2	2	999
5	5	3	2	2	2	3	1	3
4	4	3	3	2	2	3	1	3
5	5	2	2	1	3	3	1	2
3	3	3	1	1	1	1	1	3
2	2	3	3	2	3	1	2	2
4	5	2	1	2	2	2	1	2
2	2	3	1	2	3	1	1	2
5	5	2	1	1	2	1	1	2
5	5	2	3	3	3	2	1	3
3	5	3	3	1	3	2	1	3
5	5	3	1	2	3	1	2	2
4	5	3	1	1	3	1	1	3
4	4	3	1	1	2	2	1	3
1	1	3	1	3	3	2	3	3
3	4	3	1	2	3	2	1	2
1	3	2	1	2	3	3	1	2
4	4	3	2	2	3	2	1	3
2	2	2	1	1	2	1	1	1
4	5	3	3	1	3	1	1	2
3	5	3	2	2	3	3	1	2
3	3	2	3	3	3	1	1	3
4	4	3	2	1	3	1	1	2
3	3	3	1	2	2	1	2	2
5	5	3	1	1	3	1	3	2
1	5	3	1	3	3	2	2	3
2	5	2	1	3	3	2	1	2
3	5	3	3	2	3	2	1	2
4	5	3	2	1	2	2	1	2
4	5	3	1	2	3	1	1	2
5	3	3	1	2	3	1	2	3
2	3	2	2	3	2	3	2	3
4	4	2	3	2	2	2	1	2
1	5	3	3	1	3	2	3	3
5	4	3	2	1	2	2	1	2
5	5	3	3	2	3	2	1	3
4	5	3	2	1	3	1	1	2
5	5	3	2	1	3	2	1	2
4	5	3	2	2	3	2	1	2
3	5	3	1	1	2	999	1	2
3	4	3	2	1	3	2	1	3
3	4	3	1	1	2	2	1	3
4	5	3	1	3	3	1	2	3
4	5	3	2	1	3	1	1	3
2	5	3	3	1	3	2	1	3
3	5	2	3	1	2	2	1	2

5	5	3	2	1	3	2	1	2
3	5	3	2	1	2	2	1	2
2	3	3	1	2	3	1	1	3
2	4	2	2	3	2	2	3	2
3	4	3	2	2	2	2	1	2
5	5	3	2	1	3	1	1	3
4	5	3	1	1	2	3	1	2
5	4	2	2	3	2	3	1	2
4	5	3	2	1	3	2	1	3
5	5	3	2	3	3	3	3	2
4	5	3	3	1	3	1	1	3
5	5	2	1	1	3	3	1	2
4	4	3	1	1	3	1	1	2
4	5	3	1	1	3	1	1	3
5	5	3	2	1	3	3	1	2
4	5	3	1	1	3	1	1	3
5	5	3	1	1	3	2	1	3
4	5	2	1	1	3	1	2	3
4	5	2	3	2	3	1	1	2
3	3	3	2	1	3	2	1	3
4	4	3	3	1	3	2	1	3
3	5	3	2	1	3	1	1	3
4	5	2	2	1	3	2	1	2
2	5	3	1	1	3	2	1	3
5	5	3	2	1	2	2	1	1
4	5	3	2	1	3	2	1	2
4	3	3	3	3	3	2	1	2
2	2	3	2	1	3	1	1	3
5	5	3	1	2	2	2	1	3
5	5	2	2	1	2	2	1	2
3	3	2	3	1	3	2	1	2
4	4	2	1	1	2	2	1	2
5	5	3	2	2	3	2	2	2
4	4	2	2	1	1	3	1	2
5	5	3	1	2	3	1	1	3
4	4	3	3	1	3	1	1	3
5	5	3	1	1	2	2	1	3
4	4	3	2	1	2	1	1	2
3	4	3	1	1	3	1	1	2
4	5	3	2	1	3	2	1	2
4	5	3	2	1	3	1	1	3
4	5	3	2	2	3	1	1	3
3	4	2	3	1	2	2	1	3
5	4	3	3	2	3	1	1	3
5	5	3	2	1	3	1	1	2
5	4	2	2	1	3	3	1	3
5	5	3	1	1	3	1	1	3
5	5	3	1	1	2	2	1	3
5	5	3	3	2	3	1	1	3
5	5	3	2	1	2	1	1	3

4	5	3	2	1	3	1	1	3
1	2	3	2	1	2	1	2	3
3	4	2	1	1	3	2	1	3
4	5	3	1	1	3	1	2	2
4	4	3	2	1	3	1	2	3
4	5	3	2	1	3	2	1	2
4	5	3	3	3	2	2	1	2
5	5	3	1	1	3	3	1	2
4	4	3	3	1	3	2	1	2
4	5	3	2	1	3	999	1	2
5	5	3	3	1	2	2	1	3
4	3	3	1	1	2	1	1	2
5	5	2	1	1	3	2	1	2
5	5	3	2	1	3	1	1	3
3	3	2	3	1	3	1	1	2
4	5	3	1	2	3	2	1	3
4	4	2	1	1	3	1	1	3
4	5	3	2	1	3	1	1	3
5	5	3	1	1	3	3	2	3
4	5	2	3	1	2	3	2	1
5	5	3	2	1	3	3	1	2
4	5	3	3	1	3	2	1	2
3	3	3	2	1	2	1	2	3
5	5	3	1	1	3	2	2	3
4	5	3	2	1	3	1	1	3
4	4	3	1	1	3	2	1	3
5	5	2	3	1	3	3	1	2
3	5	2	2	1	2	2	1	2
4	4	3	1	1	3	2	1	2
5	5	3	3	1	3	2	2	3
1	5	2	2	1	2	2	1	3
2	3	2	1	2	2	2	2	3
4	4	3	2	2	2	1	1	2
4	5	3	2	3	3	2	1	3
4	4	3	3	2	2	3	1	2
3	5	3	2	3	3	2	2	3
3	3	3	2	2	2	3	2	2
3	3	2	2	3	2	3	1	2
3	5	3	1	2	3	1	1	2
4	3	3	2	2	3	1	1	2
4	5	3	3	3	2	2	1	3
2	5	3	1	3	3	3	2	3
4	3	3	3	3	3	2	1	2
4	4	3	2	2	3	2	1	2
1	4	3	1	3	3	3	1	3
1	1	3	3	1	3	1	2	2
2	5	2	1	2	3	2	1	2
4	3	3	2	1	3	2	1	3
5	5	3	2	1	3	1	1	2
2	2	3	2	3	3	2	1	3

5	5	3	2	1	2	3	2	2
4	4	3	2	2	2	2	1	2
4	5	2	2	1	3	3	2	1
4	3	3	2	2	3	1	1	3
3	4	3	3	1	3	1	1	2
4	3	2	1	2	3	3	1	3
5	5	3	1	1	3	2	1	2
5	5	3	2	1	3	2	1	3
4	5	3	2	2	2	1	999	2
3	5	3	2	1	3	1	1	3
4	4	3	2	2	3	1	1	3
4	4	3	1	1	3	1	1	3
5	5	3	1	1	2	2	3	3
4	5	3	1	1	3	2	1	2
4	5	3	3	3	3	2	1	2
4	5	3	1	1	3	2	1	3
4	4	2	1	1	3	1	2	2
4	3	3	1	2	3	3	2	2
5	5	3	2	1	3	2	1	3
1	2	3	2	1	3	1	1	2
5	5	2	2	1	2	3	1	2
4	5	3	2	2	3	1	1	3
4	5	3	2	2	3	2	1	3
4	4	2	2	1	2	1	2	1
4	5	3	1	1	2	1	1	3
5	5	3	1	1	3	1	3	2
4	4	3	3	3	2	2	2	2
4	4	3	2	2	2	2	1	2
4	4	3	1	2	3	2	2	3
4	4	3	2	3	3	1	1	3
4	5	3	1	2	3	1	1	2
5	999	3	2	1	3	2	2	3
3	5	3	1	1	3	1	1	3
4	5	3	2	1	2	3	1	3
4	3	3	2	2	3	2	1	2
4	4	2	1	1	1	2	1	3
5	5	3	2	3	3	1	1	2
4	5	2	3	1	2	2	1	2
4	5	3	2	1	3	2	1	2
5	5	3	2	1	3	2	1	3
5	5	3	2	1	3	1	1	3
4	4	3	2	1	3	1	1	2
2	4	2	2	1	2	3	1	1
3	5	2	3	2	2	1	1	3
2	2	2	3	2	3	3	1	3
4	4	3	2	1	3	1	1	2
5	5	3	3	2	3	2	2	2
3	4	3	2	1	2	2	1	2
4	5	3	1	1	2	1	1	3
5	5	3	2	1	3	1	1	3

5	5	3	1	1	3	2	1	999
4	5	3	1	1	2	2	1	2
5	5	3	1	2	2	2	1	3
5	5	3	1	1	3	1	1	3
3	4	3	1	1	3	1	1	3
4	5	3	1	1	3	1	1	2
5	5	3	2	2	3	1	1	3
4	4	2	1	1	3	2	1	2
5	5	3	2	1	3	1	1	2
4	4	3	2	3	3	1	1	3
4	4	2	2	1	3	1	1	3
1	4	3	2	3	3	2	1	2
4	3	3	3	1	2	2	1	2
3	4	2	2	1	2	1	2	2
5	4	3	2	2	3	3	1	2
3	5	2	3	2	3	3	1	3
3	2	3	3	2	2	2	1	3
2	3	2	1	3	2	1	2	3
2	4	3	1	2	2	3	1	2
4	5	3	1	1	2	1	1	3
4	4	3	3	1	3	1	2	3
4	5	3	2	3	3	1	1	3
2	3	3	2	2	3	1	1	3
4	4	2	2	1	2	1	1	2
1	2	3	999	1	2	2	2	2
4	5	3	1	1	2	1	1	2
3	2	3	1	1	2	1	3	3
2	2	2	2	1	2	3	1	3
2	2	1	1	1	1	2	1	1
2	5	3	2	3	1	1	1	2
4	4	3	1	1	3	1	1	3
999	2	3	2	1	3	2	2	2
4	5	3	2	1	2	3	2	3
5	4	2	1	1	3	2	1	2
3	1	2	1	2	3	3	1	1
5	5	2	2	2	3	3	1	2
2	3	3	1	1	1	1	2	3
2	5	3	2	1	3	2	1	3
2	4	2	3	1	2	1	1	2
2	5	3	2	1	3	1	1	2
1	3	3	1	1	3	3	1	3
3	4	3	1	2	3	1	1	3
4	2	2	2	1	3	3	1	2
4	5	2	1	1	2	1	1	3
4	3	3	1	1	3	1	1	3
1	4	2	3	2	3	3	1	2
5	1	3	2	1	3	1	2	2
3	3	2	1	2	3	1	1	3
2	4	2	2	3	2	1	3	999
1	3	3	1	1	3	1	3	3

3	4	3	3	1	2	2	3	3
4	3	3	1	1	2	1	1	3
1	2	2	3	1	2	1	2	3
3	2	3	2	1	3	2	2	3
2	3	3	1	1	3	1	1	3
2	3	2	1	1	2	1	3	3
5	5	2	3	2	2	3	1	1
5	5	3	1	1	3	1	1	3
4	5	3	1	1	3	3	1	2
2	3	2	3	3	3	3	2	2
2	1	2	1	1	2	1	1	2
4	5	2	2	1	3	3	1	3
4	5	3	1	1	3	2	1	3
1	4	3	2	1	3	2	2	2
5	4	3	3	1	2	1	1	3
4	4	3	2	1	3	1	1	3
4	5	2	2	1	3	2	1	3
2	3	3	1	1	3	2	1	3
5	5	3	3	1	3	1	1	3
5	5	3	1	2	2	1	1	3
4	5	3	2	1	3	1	1	3
2	1	3	1	1	3	1	1	2
5	5	3	2	1	3	2	1	3
4	5	3	2	1	2	1	1	2
4	4	2	3	1	2	3	1	2
4	5	3	1	1	3	1	1	2
3	5	2	2	1	2	3	1	2
3	5	2	1	1	3	3	1	3
4	4	3	3	2	3	1	1	3
4	3	3	2	1	3	1	1	3
5	5	3	1	1	3	1	2	3
5	5	3	3	1	3	2	1	3
4	3	3	2	1	3	2	1	3
4	5	2	2	1	2	2	1	2
1	5	1	1	2	1	1	3	3
3	3	3	2	1	3	3	2	2
3	4	2	3	1	2	2	3	3
1	2	3	1	1	3	1	1	3
2	2	3	2	1	2	3	1	3
1	2	2	3	2	1	1	1	3
5	4	3	1	1	3	2	1	2
5	4	2	3	1	3	1	1	2
3	3	3	2	1	3	1	1	2
4	5	3	2	1	3	1	1	2
2	2	3	1	1	2	2	1	2
3	5	3	1	1	3	1	1	3
3	3	2	3	1	2	3	1	2
3	5	2	2	1	2	2	1	3
4	5	2	1	1	2	1	1	2
5	5	3	3	3	1	3	3	1

1	1	3	3	1	1	1	2	2
3	4	3	3	1	3	2	1	2
1	5	2	1	2	3	3	2	2
5	5	3	3	1	3	2	1	1
2	3	2	1	2	1	2	1	2
3	4	3	2	1	3	2	1	1
1	1	1	2	2	1	3	2	2
5	5	3	2	2	3	2	1	3
3	3	3	1	1	3	3	2	3
5	3	2	3	2	2	1	1	3
3	3	2	1	1	3	1	1	2
2	5	3	2	1	2	1	1	2
2	2	3	2	2	3	2	1	2
4	5	2	2	3	3	1	2	3
5	4	3	2	1	3	1	1	3
4	5	3	3	2	3	3	1	2
4	5	3	1	1	2	2	1	3
2	4	3	1	2	3	1	2	3
3	4	3	1	2	2	2	1	2
2	3	3	1	2	2	1	1	2
1	2	2	1	1	2	1	1	1
5	5	2	3	2	2	2	2	2
3	3	3	1	1	2	1	2	2
2	3	3	3	1	2	3	3	1
3	3	2	3	2	2	3	1	2
3	4	2	3	1	2	2	1	3
4	4	3	1	1	3	1	1	3
4	3	3	2	1	3	1	1	3
3	5	3	2	1	2	2	1	2
3	5	3	2	2	3	3	1	3
4	4	3	2	2	3	2	1	3
3	5	3	3	1	3	3	1	2
3	4	3	2	1	3	1	1	3
3	4	3	2	2	2	2	1	2
4	3	3	1	1	2	2	2	2
5	5	3	1	1	2	2	1	3
1	4	3	2	2	2	1	1	2
4	4	3	2	1	2	2	2	2
3	4	2	2	1	3	2	1	2
2	2	2	3	1	2	3	1	2
3	3	3	2	1	3	2	1	2
3	3	3	2	1	2	1	1	2
4	3	3	1	1	3	1	1	2
3	2	3	3	1	3	2	1	3
4	5	2	1	1	2	3	2	3
5	5	2	3	1	2	2	3	2
1	2	2	1	1	3	3	1	2
5	3	2	1	2	2	3	2	2
2	3	2	1	1	2	1	1	3
2	4	3	2	1	3	1	1	3

4	5	3	2	2	2	2	1	1
3	3	3	1	1	3	1	2	3
4	4	2	1	1	3	1	1	3
2	3	2	1	1	3	1	1	2
3	3	2	3	1	3	2	3	1
4	4	3	2	2	2	2	1	2
2	1	1	2	1	1	2	3	2
4	5	3	1	1	2	1	2	2
3	3	3	3	1	3	1	1	2
2	4	3	3	1	2	3	2	2
5	5	2	1	1	2	2	1	2
1	5	3	1	2	2	2	1	3
4	5	2	1	1	3	2	1	2
3	3	2	2	1	3	1	1	2
1	1	3	1	1	3	1	1	2
1	5	3	3	1	3	1	1	3
2	5	3	1	1	2	1	1	3
4	5	3	2	1	3	1	1	3
999	999	3	2	1	3	2	1	3
5	5	3	2	1	3	3	1	2
3	5	2	3	1	3	3	1	1
1	3	3	1	1	3	1	1	3
1	2	2	3	1	2	1	2	2
1	2	2	1	1	2	2	1	3
5	5	3	2	1	3	3	1	3
3	3	3	1	1	2	1	1	2
5	5	3	1	2	2	3	1	3
4	4	3	1	1	2	2	1	3
3	5	3	2	1	2	2	1	2
4	3	3	2	1	3	2	1	3
4	5	3	3	1	3	1	1	999
4	5	3	1	1	2	1	1	3
3	4	3	2	1	2	2	1	2
3	2	3	3	1	3	3	3	2
1	4	3	2	1	3	1	1	3
5	5	3	3	1	2	1	1	2
3	5	3	1	2	2	2	1	2
5	4	3	1	1	1	2	1	3
2	2	3	1	1	2	3	3	1
4	5	2	1	1	3	3	2	2
2	3	2	2	1	2	2	1	2
3	3	3	2	1	2	1	1	2
4	4	3	1	1	3	2	1	3
5	5	3	3	2	3	2	1	3
3	5	3	2	2	2	1	1	2
3	3	3	1	2	2	2	1	2
4	5	3	1	2	2	3	1	3
3	3	2	3	2	1	2	3	2
2	3	3	1	1	2	2	2	3
2	2	3	2	1	3	1	1	3

2	4	2	3	1	2	2	1	3
2	3	3	1	1	1	1	1	2
4	4	3	1	1	3	2	1	2
5	4	3	3	1	1	2	1	2
3	3	3	2	1	2	1	1	2
3	3	3	2	1	2	2	1	2
2	4	3	2	2	2	2	2	2
3	2	2	2	1	2	2	1	2
4	5	3	2	1	3	2	2	2
4	4	3	1	1	3	1	1	2
4	5	3	1	1	2	2	1	2
4	3	3	1	1	3	1	1	3
1	3	3	1	1	2	2	1	2
4	4	3	2	1	3	1	1	3
4	4	3	2	1	3	2	1	3
3	4	2	3	1	3	2	2	3
3	4	3	2	1	3	2	2	3
3	3	3	1	1	3	1	1	3
2	3	2	3	2	3	1	1	2
5	4	3	1	2	2	3	1	2
5	5	3	3	1	3	2	1	2
5	5	3	2	1	3	1	1	3
5	4	3	2	2	3	2	1	2
2	2	2	2	1	2	2	1	2
999	3	3	2	1	3	2	1	2
2	4	3	2	1	3	1	1	2
3	3	3	1	2	2	1	1	2
3	3	2	3	1	1	1	1	3
4	5	3	1	1	3	1	1	3
4	4	3	2	1	3	2	1	2
1	2	2	1	2	2	3	1	2
4	5	2	3	2	2	1	1	2
3	3	3	1	2	2	1	1	2
2	4	3	2	1	3	2	1	2
3	3	2	2	2	2	3	1	3
4	5	2	2	1	2	2	1	3
1	5	3	3	2	1	3	1	2
3	3	2	2	1	2	2	1	2
3	5	3	2	1	2	2	1	2
5	5	2	3	1	2	3	1	2
4	4	3	2	1	3	3	2	3
4	4	2	2	1	2	3	1	2
4	5	3	2	1	2	2	1	1
4	5	3	1	2	3	2	1	3
3	3	3	3	2	3	3	1	2
4	4	3	3	1	3	2	1	2
4	3	3	1	1	2	1	1	3
2	3	2	3	1	2	1	1	2
3	4	3	2	1	3	1	2	2
4	5	3	1	1	3	1	2	2

4	5	2	2	1	2	3	1	1
3	4	3	3	1	3	1	1	999
3	4	2	1	1	2	2	2	3
3	5	3	1	1	2	3	1	3
4	4	3	1	1	3	1	1	3
2	3	3	3	1	3	3	1	2
3	4	3	1	1	3	2	1	3
2	4	3	3	1	3	1	1	2
1	3	3	2	1	3	2	3	3
3	3	3	2	1	2	1	1	2
2	3	3	1	1	3	3	1	2
3	4	3	1	1	3	1	1	2
2	5	3	2	1	3	2	1	2
1	4	3	1	1	3	1	1	3
3	4	2	1	1	3	3	1	2
3	3	3	1	1	3	2	1	3
4	3	3	1	1	3	2	1	2
2	3	3	2	1	2	3	2	2
2	3	3	2	1	3	3	1	2
3	3	2	2	1	3	2	1	2
3	3	3	2	1	3	2	2	2
2	2	3	2	2	2	2	1	2
3	4	2	1	1	3	2	1	3
3	3	2	1	1	2	1	1	3
4	4	3	2	1	3	1	1	2
4	4	3	2	1	3	3	1	2
4	4	2	1	1	3	2	1	3
4	4	3	2	1	3	1	1	2
3	3	3	1	1	3	2	1	3
4	5	3	2	1	3	1	1	2
3	4	3	2	1	2	1	1	2
5	5	3	2	1	3	1	1	2
2	4	2	1	1	3	2	1	2
3	2	3	2	1	2	3	1	2
4	4	3	2	1	2	2	1	2
5	5	3	1	2	2	1	2	2
4	4	2	2	2	3	2	1	2
3	4	2	3	1	2	3	1	3
4	5	2	3	3	1	3	3	1
4	3	3	2	2	3	3	1	3
5	5	3	1	1	3	1	1	3
3	3	3	1	2	3	1	1	2
3	2	2	1	1	3	2	1	2
5	5	3	2	1	2	1	1	2
4	4	2	2	1	2	2	1	2
5	5	3	2	1	3	2	1	2
5	5	3	3	3	3	1	2	1
3	4	3	1	1	2	1	1	3
4	5	3	1	2	3	1	1	3
1	3	1	1	2	3	3	1	1

2	3	2	2	1	3	3	1	3
4	4	3	2	1	3	1	1	2
3	4	2	3	1	3	2	2	2
5	5	3	1	2	3	1	1	3
2	3	3	2	1	3	3	1	2
4	4	3	3	2	3	2	1	2
4	4	3	2	2	3	1	1	2
5	5	2	3	2	3	2	1	3
3	3	3	3	2	2	2	1	2
4	3	3	2	1	3	2	1	2
3	3	2	2	1	3	1	2	2
3	3	2	1	2	2	1	1	2
4	4	3	1	1	2	2	1	3
4	5	3	2	1	2	1	1	2
2	4	2	2	2	3	2	2	2
5	5	2	1	1	3	2	1	2
3	4	3	1	1	2	2	1	2
2	3	3	2	1	3	2	1	3
4	4	3	2	1	2	3	1	3
3	3	3	3	2	2	2	1	2
2	2	1	1	1	2	1	1	2
3	3	3	2	1	3	2	1	2
3	2	2	1	1	2	2	1	3
4	5	3	1	1	2	1	2	3
3	2	3	1	1	2	2	1	2
3	4	3	1	1	2	3	1	2
3	3	2	3	1	2	1	1	1
4	3	2	2	1	3	3	1	2
2	3	3	2	1	2	1	2	2
1	1	2	1	1	2	3	1	2
2	2	3	2	1	3	1	2	3
4	4	2	2	1	3	3	1	3
5	5	3	1	1	3	2	2	3
3	1	2	2	1	1	1	3	2
2	3	2	1	1	3	2	1	2
3	4	2	3	1	2	2	1	3
5	5	3	1	1	3	1	1	3
3	3	2	1	2	3	1	1	2
999	4	3	2	1	3	1	1	2
4	3	2	2	1	3	2	1	3
3	3	3	1	1	1	2	2	3
4	3	3	2	1	2	2	1	2
3	5	2	1	1	2	2	1	3
3	4	3	1	2	3	2	1	3
3	3	3	3	2	2	3	1	2
4	4	2	2	1	3	2	1	2
5	5	3	3	1	3	2	2	2
2	4	3	3	1	2	3	2	2
5	2	3	2	1	3	2	1	3
3	4	3	3	2	3	1	2	2

4	5	3	3	2	3	2	1	2
4	4	3	1	1	3	1	2	2
3	3	3	1	2	2	2	2	2
4	4	3	2	1	3	2	1	3
3	4	3	1	1	3	1	1	2
5	5	3	2	1	2	2	1	3
4	5	3	2	1	2	1	1	3
4	4	3	2	3	3	1	1	2
1	1	2	3	1	2	1	2	2
3	4	2	3	1	1	3	1	2
5	5	3	1	1	3	1	1	2
2	4	2	3	2	3	2	2	3
4	5	3	2	1	2	2	1	2
3	4	3	3	1	3	2	1	2
3	4	3	1	1	3	2	1	2
4	5	3	1	3	3	2	1	2

SDQ8	SDQ9	SDQ10	SDQ11	SDQ12	SDQ13	SDQ14	SDQ15	SDQ16
2	3	1	2	1	1	2	2	2
1	3	2	3	1	1	3	2	2
2	3	2	3	999	2	3	3	3
1	3	2	3	1	1	3	1	1
3	3	2	3	1	3	2	1	3
2	3	1	3	1	2	3	1	1
2	3	2	3	1	2	3	3	3
2	3	1	3	1	2	2	3	3
3	3	2	3	1	3	2	3	3
2	3	1	3	1	1	3	1	1
3	2	3	3	1	3	1	3	3
2	3	1	1	1	1	3	3	2
2	3	1	3	999	1	3	2	999
1	2	1	3	1	2	3	2	1
1	3	1	3	1	1	3	1	1
2	3	1	3	1	1	2	1	2
1	3	1	3	1	1	3	1	1
2	3	1	1	1	2	3	2	2
3	3	3	2	1	3	1	3	3
2	3	1	3	1	2	2	1	2
1	3	1	2	1	1	3	2	1
2	3	2	3	1	3	2	3	2
2	3	1	3	1	2	3	2	1
2	3	2	3	1	3	2	3	2
1	3	3	3	1	2	2	2	2
2	3	1	3	1	2	3	1	1
2	3	2	3	1	2	2	3	3
3	3	999	3	999	2	3	3	3
3	3	3	3	2	2	2	3	3
2	2	3	3	2	2	3	2	2
1	3	1	3	1	3	3	3	1
2	999	1	999	999	999	2	3	999
2	2	2	3	1	2	3	3	2
1	3	3	3	1	2	3	3	1
2	3	2	3	1	2	3	1	2
3	2	3	3	1	3	2	3	3
2	1	1	3	1	2	3	2	3
3	3	1	3	1	2	3	3	1
3	2	1	3	2	2	2	2	2
2	2	2	3	1	2	2	2	1
1	3	2	3	1	1	3	1	2
3	3	2	3	1	1	3	2	2
2	3	2	3	1	1	3	2	1
2	3	1	3	1	1	3	2	1
1	2	2	2	1	1	2	2	1
2	3	2	3	1	3	2	2	2
2	1	1	3	1	1	3	1	1
2	3	2	2	1	2	2	3	3
1	3	2	3	1	1	3	1	1

2	3	2	3	1	2	3	2	1
1	3	2	3	1	1	3	3	2
1	3	1	3	1	1	2	2	1
2	3	2	3	1	2	3	3	3
2	2	2	3	1	2	3	2	3
2	3	2	3	1	1	3	2	3
3	3	3	2	1	3	3	2	3
2	3	3	2	1	1	2	2	1
2	3	2	3	1	1	3	3	2
2	2	1	3	1	2	3	2	2
2	3	1	3	1	2	3	2	2
2	3	2	3	1	2	3	2	3
2	3	2	1	1	2	2	3	3
2	3	1	3	1	2	2	2	2
2	3	3	3	1	1	2	3	2
2	3	1	3	1	2	3	2	1
1	3	1	3	1	1	3	1	1
2	2	1	3	1	2	2	1	1
2	3	2	3	1	1	3	2	1
2	3	1	3	1	1	3	1	2
2	3	2	3	1	2	3	1	999
2	3	1	3	1	1	3	1	2
1	3	2	3	1	2	3	1	2
2	3	3	3	1	2	2	3	3
1	3	2	3	1	1	3	2	1
2	3	1	3	1	1	3	1	1
2	3	3	3	1	2	3	2	1
2	3	2	3	1	2	2	3	2
3	3	3	3	1	1	3	2	2
2	3	1	3	1	1	3	3	2
2	2	2	3	1	3	2	2	2
1	2	3	3	1	2	3	3	3
1	3	3	3	1	1	3	3	1
3	3	1	3	1	3	3	2	1
3	3	3	2	1	3	2	2	1
3	3	3	3	3	2	2	3	2
1	3	3	3	1	2	2	3	2
1	3	1	3	1	1	2	2	2
2	3	3	3	1	1	3	2	1
1	3	2	2	1	1	2	1	1
2	2	1	3	1	2	3	3	3
2	2	2	2	1	1	2	2	2
1	3	3	3	1	1	2	3	1
3	3	3	3	1	1	2	2	999
1	2	2	3	1	1	1	3	2
1	2	3	3	1	1	2	2	2
1	2	2	3	1	2	3	2	2
2	3	1	3	1	1	3	1	1
1	3	1	3	1	1	3	1	1
1	3	1	3	1	1	3	1	2

2	2	3	3	2	2	2	3	2
2	3	1	2	1	1	2	2	1
1	3	2	3	1	1	3	1	1
1	2	1	2	1	3	2	2	3
3	3	3	3	1	3	3	1	1
1	1	1	3	1	1	3	1	1
1	3	2	3	1	2	3	2	2
3	3	3	3	1	3	3	3	3
2	3	1	3	1	1	3	3	2
1	3	1	3	1	1	3	1	1
3	2	2	2	1	2	3	2	2
2	3	2	3	1	1	3	2	2
2	2	3	3	1	2	2	3	2
1	3	1	3	1	1	2	2	1
1	3	2	3	2	1	3	2	1
2	3	1	3	1	2	2	2	2
1	2	1	3	1	2	3	2	2
1	3	3	3	1	2	2	2	1
3	3	2	3	1	2	3	1	3
2	3	1	2	1	2	2	3	1
2	3	1	3	1	1	3	2	1
1	3	1	999	1	1	2	2	1
1	3	1	3	2	1	3	2	1
2	2	2	2	1	1	2	2	2
3	3	1	3	1	1	3	3	3
3	2	3	3	1	1	3	3	2
2	3	3	2	1	2	3	3	3
1	3	2	1	1	1	2	2	3
1	3	1	3	1	1	2	2	1
2	3	3	3	2	3	2	3	3
2	3	2	3	1	2	3	3	1
1	3	2	3	1	1	3	3	1
2	3	1	3	1	2	3	3	3
3	2	3	3	1	3	3	3	3
2	3	3	3	2	2	2	2	3
2	3	3	3	1	2	3	2	2
2	3	2	3	1	1	3	3	2
3	3	2	3	1	3	1	2	2
3	3	2	3	1	1	3	1	2
1	2	1	3	1	1	2	3	2
1	3	2	3	1	1	3	3	2
2	3	2	3	2	1	3	2	3
2	3	3	3	1	2	3	3	2
3	2	1	3	1	2	2	1	3
1	2	1	3	1	2	3	3	1
1	3	1	3	1	1	2	1	1
3	3	3	999	2	3	3	3	3
1	3	2	3	1	1	3	3	3
2	3	2	2	1	2	2	3	2
2	999	3	3	1	3	3	2	2

3	2	3	3	1	1	3	3	3
2	3	1	2	1	1	3	2	2
1	3	1	3	1	1	2	2	2
2	3	2	3	1	2	2	1	2
2	3	3	3	1	2	2	3	999
1	3	2	3	1	1	3	1	1
2	3	1	3	1	3	2	2	2
1	3	1	3	1	1	2	2	2
2	3	3	3	1	1	3	1	2
1	3	2	3	1	2	3	3	2
1	3	2	3	1	2	3	3	2
2	3	1	3	1	1	3	3	2
3	3	2	3	1	3	2	3	2
2	3	1	3	1	2	2	3	2
1	3	1	3	1	1	2	2	1
3	3	3	3	1	1	2	3	3
2	2	2	3	1	3	2	3	2
2	3	1	3	1	1	3	1	1
1	3	2	3	1	2	2	1	2
3	3	1	3	1	1	1	3	3
3	3	3	3	1	2	3	3	3
3	3	1	3	1	1	3	3	3
1	3	1	3	1	1	3	2	1
2	3	3	3	1	2	3	2	1
2	3	3	3	1	1	3	3	2
1	3	2	3	1	1	3	2	2
2	3	1	3	1	1	2	1	2
1	3	2	3	1	1	3	1	2
2	3	2	3	1	2	3	2	3
2	3	2	3	1	1	3	2	2
2	3	2	3	1	2	3	3	1
3	3	1	2	1	2	2	3	2
3	3	2	3	1	1	3	2	2
1	2	1	3	1	3	1	2	3
3	2	2	3	1	2	2	2	2
2	2	2	3	1	2	3	2	2
3	2	1	3	1	2	2	3	2
1	3	3	3	1	1	2	2	3
1	3	1	3	1	1	3	2	1
2	3	1	2	1	1	3	2	1
3	2	2	3	1	3	2	2	3
1	3	1	3	1	1	2	2	3
2	3	2	3	1	1	3	1	1
1	3	2	3	1	2	3	3	3
1	2	3	3	1	2	2	2	1
1	3	1	3	1	2	2	2	1
1	3	3	3	1	1	3	3	2
2	3	1	3	1	1	3	3	2
2	3	2	3	1	1	3	3	1
1	3	2	3	1	1	3	1	2

1	3	3	2	1	1	3	3	2
2	2	1	3	1	2	2	2	3
1	3	1	3	1	1	3	2	2
3	2	2	3	2	2	2	3	3
1	3	2	3	1	1	2	3	1
3	3	3	3	3	2	3	3	3
1	3	3	3	1	2	3	2	2
2	3	2	3	2	2	3	1	2
2	3	3	1	1	2	2	3	2
2	3	2	1	1	1	2	999	2
2	3	1	3	1	3	2	1	2
1	3	2	3	1	1	3	3	3
1	3	1	3	1	1	2	1	2
2	3	2	3	1	1	3	1	1
2	3	2	3	1	1	3	3	2
2	3	2	3	1	1	3	2	2
1	3	1	3	1	1	3	3	1
1	3	1	3	1	1	3	2	2
2	3	2	3	1	1	3	2	2
1	2	1	2	1	1	3	2	999
2	3	1	1	1	1	1	3	3
1	3	3	3	1	1	3	2	2
1	3	1	3	1	1	2	2	1
2	2	2	2	1	3	2	2	3
3	3	2	3	1	3	2	3	3
1	3	2	3	1	1	3	1	1
1	2	2	3	1	1	3	2	2
2	2	2	3	1	2	3	2	1
1	2	2	2	1	1	3	2	1
2	2	2	3	1	1	3	1	1
1	3	1	3	1	1	2	2	3
1	3	2	3	1	1	3	2	2
1	2	2	3	1	1	2	2	2
1	3	3	3	1	1	3	2	2
2	2	2	3	2	2	3	3	2
2	3	3	3	1	1	3	3	2
1	3	3	3	1	1	3	1	2
1	3	2	3	1	1	2	2	1
1	3	3	3	1	1	2	1	3
1	3	3	3	2	1	3	2	1
2	2	1	3	1	1	2	2	2
1	3	3	3	1	1	2	3	1
2	2	1	3	1	1	3	1	2
2	3	1	3	1	1	3	1	2
2	2	1	3	1	2	2	3	3
2	3	2	3	1	2	3	3	3
2	3	1	3	1	1	2	1	2
1	2	2	3	1	1	3	2	1
2	3	2	3	1	1	2	1	2
999	999	999	999	999	999	999	999	999

3	3	2	3	1	3	2	1	2
3	3	2	3	1	3	3	3	3
2	3	1	3	1	2	2	3	2
3	2	3	3	1	2	2	2	3
3	3	3	3	2	2	2	1	3
2	3	2	3	1	2	2	3	2
2	3	3	3	1	3	2	2	3
2	3	2	3	2	2	3	1	1
2	3	2	3	1	2	3	2	2
3	3	3	2	1	3	2	2	3
2	2	1	3	1	1	2	2	2
2	2	2	3	1	2	2	2	2
3	2	1	3	2	1	3	2	3
3	3	1	3	2	2	2	2	3
3	3	3	3	1	2	2	1	3
3	2	1	3	1	2	3	1	2
2	3	1	3	1	1	2	2	2
999	2	1	3	1	2	2	2	2
2	3	1	1	1	3	1	2	2
2	3	1	3	1	2	3	2	3
2	3	2	3	1	1	2	3	3
2	3	1	3	1	2	2	1	3
3	2	1	2	1	3	2	2	2
2	3	2	3	1	1	3	3	1
3	3	2	3	1	1	3	3	3
2	3	3	3	1	2	2	3	2
2	3	2	3	1	1	2	2	3
2	2	1	2	1	2	2	2	2
2	3	1	3	1	2	3	2	3
3	3	1	3	1	3	2	2	3
2	2	1	3	1	1	2	2	2
2	3	2	3	1	2	2	3	3
3	2	2	3	1	2	3	2	3
2	3	1	3	1	2	3	2	2
2	3	1	3	1	2	3	2	3
3	3	2	3	1	2	2	3	3
2	3	3	3	1	2	3	3	2
2	3	3	3	1	1	3	2	1
1	2	2	3	1	1	3	2	2
3	3	3	3	1	2	3	2	2
2	3	2	3	1	1	3	2	2
2	3	1	3	1	1	2	2	2
3	3	1	3	1	2	3	2	1
1	3	2	3	999	1	3	1	2
1	3	1	3	1	2	3	1	1
1	3	1	3	1	1	2	3	1
999	2	1	3	1	2	3	1	2
2	3	2	3	1	1	3	1	2
3	3	2	3	1	2	3	3	3
1	2	3	3	1	1	3	2	2

1	3	2	3	1	1	3	2	2
1	3	1	3	1	1	3	3	3
3	3	1	2	1	2	1	3	3
3	2	1	2	1	3	2	3	2
3	3	2	3	1	2	3	3	2
2	3	1	3	1	1	3	2	2
2	3	1	3	1	3	2	2	2
3	3	3	3	1	2	2	2	3
2	3	2	3	1	1	2	3	2
2	3	2	3	2	2	2	3	1
1	3	2	3	1	2	2	2	1
2	3	1	3	1	3	3	2	2
2	3	1	3	1	1	3	3	2
1	3	1	3	1	1	3	1	2
2	3	2	3	1	1	3	2	3
1	3	2	3	1	1	3	1	2
2	3	1	3	1	2	3	2	1
1	3	1	3	1	1	3	2	2
2	3	3	3	1	2	3	2	1
1	3	3	3	1	1	3	2	2
2	2	3	3	1	2	3	2	1
1	3	2	3	1	2	3	2	1
2	2	2	3	1	1	2	2	1
2	3	1	3	1	2	2	2	3
1	3	3	3	1	1	2	3	2
1	3	2	3	1	1	3	2	2
3	3	2	3	1	3	3	3	2
2	2	2	3	1	2	3	2	2
2	3	2	3	2	2	3	1	3
1	2	2	2	1	2	2	2	3
2	2	3	3	2	1	2	3	3
3	2	1	3	1	2	2	2	3
3	3	2	3	1	2	3	2	1
1	2	2	3	1	2	2	3	2
3	3	1	3	1	1	3	2	2
3	3	2	3	1	1	3	2	1
2	2	2	3	1	1	3	1	2
1	3	2	3	1	1	3	2	1
1	2	2	3	1	1	3	3	1
1	3	1	3	1	1	3	2	1
2	3	2	2	1	1	3	1	2
1	3	2	3	1	1	2	1	1
1	3	3	3	1	1	3	1	2
2	3	2	3	1	2	3	3	2
2	3	2	3	1	1	3	2	1
1	2	2	3	1	1	3	2	2
1	3	1	3	1	1	3	1	1
2	3	1	3	1	2	3	2	1
2	3	3	3	1	2	3	3	1
1	3	2	3	1	1	3	2	1

1	3	1	2	1	3	3	3	1
2	2	2	3	1	2	2	1	2
2	3	1	3	1	1	3	1	1
1	999	1	3	1	1	2	2	3
2	2	1	3	1	1	2	1	3
2	3	2	3	1	1	2	2	2
2	3	3	3	1	1	3	3	2
2	3	2	3	1	1	3	3	2
2	3	3	3	1	1	3	2	1
1	3	2	3	1	1	3	1	2
1	3	2	3	1	1	3	2	1
2	3	2	3	1	1	2	1	2
1	3	2	3	1	1	3	2	2
2	3	2	3	1	2	2	1	1
2	3	2	3	1	2	3	1	1
2	3	1	3	1	2	3	1	2
3	2	1	3	1	1	2	1	2
2	3	2	3	1	1	3	2	1
1	3	1	3	1	1	3	2	2
2	2	3	3	2	3	2	3	3
1	3	2	3	1	1	3	3	2
3	2	2	3	1	2	2	3	2
2	2	2	3	1	1	3	2	1
3	3	1	3	1	1	3	2	1
2	2	2	3	1	2	3	2	2
2	3	1	3	1	1	3	1	3
1	3	3	3	2	1	3	3	1
1	2	2	3	1	2	2	2	2
2	3	2	3	1	2	3	2	3
1	3	3	3	1	1	2	2	1
1	3	2	3	2	1	2	3	2
3	3	2	1	1	3	2	2	3
2	3	2	3	1	2	2	2	2
2	3	2	3	1	2	3	1	3
2	2	3	3	1	2	3	3	2
3	3	2	3	1	3	2	2	3
2	2	2	3	1	3	2	2	2
2	2	2	3	1	3	3	2	3
3	3	1	3	1	2	2	1	2
3	3	2	3	1	3	2	2	3
2	3	3	3	1	2	3	2	1
3	3	2	3	1	2	3	2	2
2	3	3	3	1	3	2	2	3
2	3	2	3	1	1	2	3	3
3	2	1	3	1	2	2	1	3
3	3	3	3	1	3	2	3	3
3	3	1	3	1	2	2	2	3
2	2	2	3	1	1	2	1	1
2	3	1	3	1	2	3	2	2
2	3	2	2	1	2	3	3	3

2	3	2	3	1	2	2	3	2
1	3	2	3	1	2	3	3	2
2	2	1	3	1	3	1	3	3
2	3	2	3	1	2	3	2	2
2	3	3	3	1	1	3	3	2
3	3	2	3	1	2	2	2	2
1	3	2	3	1	1	3	2	3
2	3	2	3	1	3	2	2	2
3	2	2	3	1	2	2	2	2
2	3	2	3	1	2	2	1	3
2	3	1	3	1	2	2	3	2
2	3	1	2	1	1	2	3	3
3	3	1	3	1	2	2	1	3
2	3	2	3	1	2	2	3	2
2	3	3	3	1	2	3	3	2
2	3	1	3	1	2	3	2	1
2	3	1	3	1	2	2	1	3
3	3	2	2	1	3	2	2	3
3	3	3	3	1	2	3	3	2
3	3	2	1	2	3	2	1	2
1	2	3	3	2	2	3	3	1
1	3	2	3	1	1	3	2	1
3	2	2	3	1	2	3	2	2
1	2	2	3	1	1	2	2	2
1	3	1	3	1	1	3	2	2
2	3	1	3	1	2	2	1	2
2	2	3	3	1	2	2	3	2
2	2	2	3	1	1	2	2	2
2	3	1	3	1	1	3	1	1
2	3	2	3	1	2	3	2	2
2	3	1	3	1	1	2	1	1
2	3	3	999	1	3	3	3	3
1	2	1	3	1	1	3	1	2
1	3	2	3	1	2	3	2	2
2	3	2	3	1	1	3	2	1
2	3	1	3	1	2	3	1	1
3	3	1	3	1	3	3	1	3
2	3	2	3	1	1	2	3	2
2	2	1	3	1	2	2	2	3
2	3	2	3	1	1	2	2	3
1	3	3	3	1	3	3	1	1
2	3	2	3	1	1	3	1	1
2	2	1	3	2	2	2	3	3
1	3	2	3	1	1	3	2	2
2	3	3	3	1	1	2	3	2
2	3	2	3	1	2	3	3	3
3	3	3	3	1	2	3	3	3
2	2	3	3	1	2	3	2	1
1	2	1	3	1	1	3	2	2
2	3	2	3	1	1	3	2	1

2	3	1	3	1	2	3	2	2
2	2	1	3	1	2	2	2	2
2	3	1	3	1	2	3	2	1
2	3	1	3	1	1	3	1	2
3	3	2	2	1	1	3	2	999
1	3	2	3	1	1	3	2	1
2	3	1	3	1	1	2	2	1
2	2	1	3	1	2	2	2	2
2	3	2	3	1	2	2	2	1
3	3	3	2	1	3	2	2	2
2	3	2	3	1	1	3	3	1
2	2	1	3	1	3	2	3	1
3	3	3	2	1	3	2	3	3
1	3	3	3	1	1	3	1	1
2	3	2	3	1	2	2	1	2
2	3	3	3	2	1	2	3	1
1	3	2	3	1	1	3	3	2
2	3	2	3	1	1	3	2	3
3	3	1	3	1	2	2	2	3
3	3	1	3	1	1	2	3	3
3	3	3	3	1	2	3	3	2
1	3	3	3	1	1	3	2	1
2	3	2	3	1	1	3	2	3
1	3	3	3	1	2	3	3	2
2	2	2	2	1	2	2	2	2
1	3	2	3	1	1	3	2	1
2	3	1	3	1	1	3	3	1
2	3	2	1	1	2	1	2	3
1	1	2	2	2	1	1	1	2
3	3	2	3	1	1	3	1	1
1	3	1	3	1	1	3	1	1
2	3	2	3	2	2	2	3	3
2	3	1	3	1	2	3	3	1
1	3	1	3	1	2	3	2	1
2	2	3	3	2	3	3	3	2
2	3	2	3	1	2	2	2	2
2	2	1	3	1	3	2	2	3
2	3	2	3	1	2	2	1	2
2	2	3	3	2	1	3	2	2
1	3	1	3	1	3	3	3	2
2	3	1	3	999	2	3	2	2
3	3	2	1	1	2	3	1	2
1	2	1	3	1	1	3	2	2
2	3	1	3	1	2	3	3	1
1	3	1	3	1	1	3	2	2
3	3	1	3	1	2	3	3	2
1	3	3	3	1	1	3	1	2
2	3	2	3	1	2	2	2	2
2	3	3	3	1	2	3	3	3
1	2	1	3	1	1	999	1	1

1	3	3	3	1	1	3	3	2
2	3	2	3	1	1	3	2	2
3	3	3	3	1	2	2	3	2
3	3	2	3	2	3	2	2	3
1	3	1	3	1	1	2	2	2
1	3	2	3	2	1	3	3	2
1	3	3	1	2	1	3	3	2
3	3	2	3	1	2	3	2	1
1	3	2	3	2	1	3	2	2
1	3	3	2	2	1	2	3	2
2	2	1	3	1	1	3	2	2
2	2	2	3	1	1	3	1	1
1	3	2	3	1	2	3	3	1
2	3	1	3	1	1	2	2	3
3	3	2	3	1	1	2	2	3
1	3	1	3	1	1	3	2	2
1	2	1	3	1	1	3	1	1
1	3	1	1	1	2	3	1	2
1	3	2	3	1	1	3	1	3
2	3	1	3	2	2	3	2	2
1	3	2	3	1	1	3	2	1
1	3	3	3	2	1	3	3	1
2	3	2	3	1	2	3	1	2
1	3	1	3	1	1	3	2	1
2	2	2	3	1	2	3	2	2
1	3	2	3	1	1	3	3	1
2	2	2	3	2	1	3	2	1
2	2	1	3	1	1	3	2	1
2	3	2	3	1	1	3	2	2
1	3	1	3	1	1	3	1	2
2	3	2	3	1	2	3	3	1
1	3	3	3	1	1	3	2	2
2	3	2	2	1	1	1	2	2
1	2	1	3	1	1	2	2	2
3	3	2	3	1	2	2	2	3
999	2	2	3	1	1	3	2	1
2	3	1	3	1	3	3	1	1
2	2	1	3	1	1	3	2	1
2	2	3	3	1	1	3	1	1
1	2	2	3	2	1	3	2	1
1	3	2	3	1	1	3	2	2
1	2	3	3	1	1	3	3	2
1	3	1	3	2	1	3	1	1
1	3	1	3	1	1	3	3	3
1	2	1	3	1	2	2	2	1
2	3	1	3	1	1	3	1	1
1	2	3	3	1	1	3	3	3
1	2	1	3	1	1	3	2	1
2	2	2	3	1	1	3	3	3
3	1	3	3	3	1	3	2	3

1	3	1	3	1	1	2	1	2
1	2	3	3	2	1	3	3	2
3	2	1	3	2	2	3	2	2
2	3	3	3	1	2	3	3	2
1	2	1	3	1	1	1	3	1
1	2	2	3	1	1	2	3	1
2	3	2	2	1	2	2	2	2
1	3	2	3	1	2	3	1	1
2	3	2	3	1	2	2	2	3
3	3	3	3	2	2	3	2	2
1	3	1	3	1	1	3	2	1
1	2	3	3	1	1	3	2	3
2	2	2	3	2	2	2	3	2
2	3	2	3	1	3	2	3	999
1	3	2	2	1	3	2	1	2
3	3	3	3	1	2	3	3	3
2	3	2	3	1	1	3	2	2
1	3	1	3	1	1	2	2	3
1	2	2	3	1	2	3	2	2
2	3	2	3	1	1	2	2	2
2	2	1	3	1	1	2	3	1
3	2	3	3	3	1	2	3	2
1	2	1	3	1	1	2	1	2
2	3	3	3	1	2	3	3	1
2	2	2	3	1	2	3	2	3
1	2	2	2	1	1	2	1	2
1	3	1	3	1	1	3	2	2
2	3	2	3	1	2	3	1	2
1	3	2	3	1	1	3	2	1
1	3	2	3	1	1	3	3	1
1	3	3	3	1	1	3	2	1
1	3	2	3	1	1	3	3	2
1	3	2	3	1	1	3	2	1
1	2	1	3	1	1	3	3	3
2	2	1	3	1	1	3	3	3
3	3	2	3	1	1	3	3	2
2	3	2	3	1	2	3	3	1
1	3	2	3	1	2	3	1	3
2	3	3	2	1	1	3	2	2
3	2	2	3	2	2	2	3	2
1	3	2	3	1	1	3	2	1
2	3	2	3	1	1	3	2	2
1	2	1	3	1	1	3	2	1
1	2	3	3	1	1	3	1	2
1	3	1	3	1	1	2	1	2
2	3	3	3	1	1	2	2	2
2	3	1	3	1	1	1	2	2
1	3	2	3	1	2	2	3	2
2	3	2	3	1	1	3	2	2
2	3	2	3	1	1	3	2	1

3	2	1	3	2	2	3	3	1
3	2	1	3	1	1	3	1	1
2	3	1	3	1	1	3	3	1
2	2	1	3	1	2	3	2	2
2	2	3	3	1	1	3	2	1
2	2	2	2	1	2	2	2	2
1	1	1	2	1	3	2	1	2
1	3	1	3	1	1	2	2	3
2	3	3	3	1	2	3	3	2
3	3	2	3	1	3	1	1	2
1	2	3	3	2	2	2	3	1
1	3	1	3	1	1	3	3	1
1	1	1	3	2	1	1	2	2
1	3	2	3	1	1	3	2	1
2	2	2	2	1	1	2	2	1
1	2	2	3	1	1	3	2	2
1	3	1	3	1	1	3	1	1
1	3	2	3	1	1	3	2	1
1	3	2	3	1	2	3	2	2
2	3	2	3	1	1	3	999	2
1	2	3	3	2	1	3	1	3
2	2	1	3	1	2	3	1	1
1	2	3	3	1	1	3	3	2
1	3	2	3	1	1	3	2	1
2	3	3	3	1	1	3	2	2
1	3	1	3	1	1	3	2	1
2	3	2	3	1	1	3	2	1
2	3	2	3	1	1	3	2	2
1	2	3	3	1	1	3	2	1
2	2	2	3	1	1	3	1	2
1	3	2	3	1	2	3	3	2
1	3	2	3	1	1	3	1	1
1	2	2	3	1	1	1	3	1
2	3	2	3	2	1	1	1	3
2	3	2	3	1	1	2	3	2
1	2	3	3	1	1	3	3	1
1	3	2	3	1	1	2	2	1
1	3	1	3	1	1	3	3	3
2	2	1	3	1	1	3	3	2
1	3	2	2	1	2	2	1	3
1	3	2	3	1	1	3	2	1
2	3	3	3	1	1	2	1	1
2	2	2	3	1	2	3	3	1
2	3	3	3	1	1	3	3	1
1	3	2	3	1	1	3	2	2
2	2	1	3	1	1	2	1	2
2	3	1	3	1	2	3	1	2
2	2	3	3	2	1	2	3	2
1	3	1	3	1	1	3	2	2
2	3	2	3	1	2	3	3	2

2	2	2	3	1	1	2	3	1
1	2	1	3	1	1	2	2	3
2	3	2	3	1	2	3	3	2
2	3	2	3	1	1	2	3	2
2	2	2	3	1	2	2	2	2
1	2	2	3	1	1	2	3	1
2	3	2	3	2	2	2	2	2
2	2	1	3	1	3	2	3	2
1	3	2	3	1	1	3	2	1
2	3	1	3	1	1	2	2	3
2	3	1	3	1	1	2	3	2
2	3	1	3	1	2	3	2	2
1	2	3	3	1	1	3	2	2
3	3	2	2	1	2	2	1	3
2	3	2	3	1	1	2	1	2
1	2	2	3	1	1	2	3	3
1	3	2	3	1	1	3	2	1
1	3	2	3	1	1	3	1	2
2	3	3	3	2	1	2	3	2
3	3	1	3	3	3	3	2	1
1	3	3	3	1	2	3	3	3
1	3	3	3	1	1	3	3	2
1	2	2	3	1	1	2	2	3
1	2	2	3	1	2	2	3	2
2	3	1	2	1	3	2	1	1
3	2	1	3	1	1	3	3	2
2	2	1	3	1	1	3	2	2
1	2	3	3	1	1	2	1	1
2	3	1	3	1	2	3	1	2
2	3	2	3	1	1	3	2	2
3	2	2	3	2	2	3	2	2
2	2	3	3	1	1	2	2	2
2	3	1	3	1	1	2	1	2
2	3	2	3	1	1	3	2	1
2	2	2	3	1	1	3	3	3
2	3	2	3	1	1	3	1	1
3	3	2	3	1	3	2	3	2
1	3	2	3	1	1	2	2	2
2	2	2	3	1	1	2	2	2
2	3	3	3	1	1	2	3	3
2	2	2	3	1	2	3	2	2
2	3	1	3	1	1	3	3	3
1	2	2	3	1	1	3	3	1
1	3	1	2	1	1	3	2	2
2	3	3	3	2	2	2	3	2
1	3	2	3	1	1	3	1	2
1	3	1	3	1	1	3	1	2
1	3	2	2	1	1	2	3	2
2	3	2	3	2	2	3	1	2
2	2	3	1	1	1	3	3	2

2	2	1	3	1	2	2	2	2
3	3	3	3	1	1	3	2	2
2	3	1	3	2	1	3	2	1
2	3	3	3	1	2	3	3	2
2	3	2	3	1	1	3	2	1
1	3	2	3	2	1	3	2	3
999	3	1	3	1	1	3	1	2
2	2	3	3	1	1	3	3	1
2	3	1	3	1	1	3	3	3
2	2	1	3	1	1	3	3	2
999	3	2	3	1	1	3	3	1
3	3	1	3	1	1	3	2	3
2	3	3	3	1	1	3	2	2
1	3	1	3	1	1	3	1	1
2	3	2	3	2	1	2	1	3
1	3	2	3	1	1	3	2	2
2	3	3	3	1	3	3	3	999
2	2	2	3	1	1	2	2	2
2	2	2	3	2	1	3	2	2
2	2	2	3	2	1	3	2	2
3	2	2	2	1	2	2	2	2
1	3	2	3	1	1	3	3	3
1	2	1	3	1	1	3	1	1
2	1	3	3	1	2	3	3	1
1	2	1	2	1	1	3	2	1
1	2	2	3	1	1	3	2	1
2	2	3	3	1	1	3	1	1
1	2	3	3	1	1	3	1	1
2	2	1	3	1	1	1	2	2
1	3	2	3	1	1	3	2	1
2	3	3	3	1	1	2	2	1
3	3	2	3	1	1	3	2	2
2	3	1	3	1	2	3	1	1
2	2	2	3	1	1	2	2	3
2	3	3	3	1	1	2	2	2
3	3	1	3	1	2	2	3	1
2	3	1	3	1	2	3	2	2
3	2	2	3	1	1	3	2	1
3	3	3	3	1	3	1	3	2
2	3	1	2	1	1	3	3	2
3	3	1	3	1	3	3	2	3
3	2	2	3	1	3	2	2	3
3	2	1	3	1	1	3	3	2
1	3	2	3	1	1	3	2	1
2	2	2	3	2	2	2	1	2
3	3	2	3	1	2	2	3	2
3	3	3	3	3	3	3	3	3
2	3	2	3	1	1	3	1	1
3	3	1	3	1	2	2	1	2
1	2	3	3	1	1	2	2	2

1	3	2	3	1	2	1	2	3
1	3	2	3	1	1	3	1	2
2	3	3	3	1	1	1	2	1
3	3	1	3	1	1	3	2	3
2	3	1	3	2	1	2	2	3
1	3	3	3	1	2	3	3	1
1	3	2	3	1	1	3	3	1
2	3	3	3	1	3	2	1	2
3	3	3	3	1	2	3	2	2
3	999	2	3	1	2	3	3	3
2	2	2	3	1	2	2	2	2
2	2	1	3	1	2	2	3	2
2	3	1	3	1	1	3	2	2
2	2	2	3	1	1	3	1	1
2	3	1	3	1	1	2	2	3
1	3	1	3	3	1	3	1	1
2	3	2	3	1	1	2	2	3
2	3	2	999	3	2	2	2	2
2	3	3	3	1	2	3	2	2
3	3	3	3	1	3	2	2	3
1	2	1	3	1	1	3	2	2
2	3	2	3	1	1	2	2	1
2	2	1	3	1	2	2	2	2
2	3	2	3	1	1	3	3	3
1	3	2	3	1	1	3	1	1
2	3	1	3	1	1	3	2	2
2	3	1	2	1	1	2	3	1
2	3	2	3	1	1	2	1	1
2	3	2	2	1	1	2	2	2
2	3	2	1	2	2	3	2	1
2	3	2	3	1	2	3	2	1
2	2	1	3	1	1	3	1	2
3	3	1	3	1	1	2	1	3
2	2	1	3	2	1	2	2	1
1	1	1	3	2	1	2	2	1
2	3	2	3	2	1	3	2	2
2	3	2	3	1	1	3	2	2
1	2	2	3	1	1	2	2	1
2	3	1	3	1	1	3	3	2
1	2	3	3	1	1	3	2	1
1	2	1	3	1	1	2	2	3
2	3	2	3	1	1	3	2	1
1	3	1	3	1	1	3	3	2
2	3	2	3	1	2	3	1	2
2	3	3	3	1	2	3	3	2
1	3	2	3	1	1	2	2	1
1	3	2	3	1	1	3	3	1
3	2	3	2	2	2	2	3	3
2	2	2	3	1	1	3	2	2
2	3	3	3	1	2	3	3	2

2	3	3	3	1	1	3	2	3
1	3	1	3	1	1	3	2	1
1	2	1	3	1	1	3	2	1
2	2	1	3	1	1	3	2	2
1	2	2	3	1	1	3	2	1
1	3	2	3	1	1	3	1	1
2	3	2	3	1	1	2	2	1
2	3	2	3	1	1	3	1	3
1	2	2	3	2	1	3	3	1
1	3	3	3	3	1	3	1	1
1	3	2	3	1	1	3	1	1
2	2	3	3	1	2	2	3	3
2	3	2	3	1	1	3	1	3
2	3	2	3	1	2	3	3	3
2	3	1	3	1	1	3	2	1
2	3	1	3	1	1	2	3	2

SDQ17	SDQ18	SDQ19	SDQ20	SDQ21	SDQ22	SDQ23	SDQ24	SDQ25
1	1	1	1	2	1	1	2	2
3	1	1	3	2	1	1	1	2
3	999	1	3	2	1	1	3	1
3	1	1	3	2	1	1	1	2
1	1	2	2	2	1	1	3	2
3	1	1	3	3	1	1	1	3
3	1	1	3	2	1	2	3	2
1	2	2	3	2	1	1	3	1
3	3	2	3	2	1	1	2	1
3	1	1	3	3	1	1	2	3
2	1	3	3	1	1	2	3	1
3	2	1	2	3	1	1	3	3
999	999	999	1	2	1	1	2	2
3	1	1	2	2	1	1	3	2
3	1	1	3	2	1	1	1	2
3	1	1	2	2	2	1	1	2
3	1	1	3	2	1	1	1	3
3	1	2	2	2	1	1	2	2
3	1	3	2	2	1	3	3	1
3	1	1	3	2	1	2	3	3
3	1	1	2	2	1	2	1	2
3	2	2	3	2	1	3	1	1
3	1	1	3	2	1	1	1	3
2	2	3	2	2	2	2	2	3
3	1	1	3	2	1	1	2	3
3	1	2	3	3	1	1	2	3
3	1	1	3	2	1	2	1	1
3	2	1	3	1	1	1	3	2
3	1	2	3	1	1	2	3	1
3	2	1	2	2	1	1	3	2
3	1	1	3	2	1	1	3	3
999	999	999	999	999	1	999	2	2
3	1	2	2	2	2	2	1	3
3	1	1	2	1	1	3	1	1
3	1	1	3	2	1	2	2	2
3	2	3	2	2	2	1	3	1
3	1	1	2	3	1	1	1	3
3	1	1	3	3	1	1	2	3
2	2	2	2	2	2	2	3	3
3	1	2	1	2	1	2	2	2
3	1	1	3	3	1	1	1	3
3	1	1	2	2	1	1	1	3
3	1	2	2	2	2	1	1	2
3	1	1	2	3	1	2	2	3
3	1	1	2	2	1	2	2	3
3	3	3	3	2	1	3	2	2
3	1	1	2	3	1	1	3	3
3	1	2	2	2	1	2	2	2
3	1	1	3	2	1	1	1	3

3	2	1	3	2	1	1	2	3
3	3	2	3	2	1	1	999	2
3	1	1	3	3	1	1	2	3
3	1	1	2	2	1	1	1	1
3	1	1	2	3	1	2	2	2
3	1	1	2	2	1	1	2	2
3	3	3	2	3	1	3	1	3
3	2	1	3	2	1	1	2	2
3	1	1	3	2	1	1	1	2
3	1	1	2	2	1	3	2	2
3	1	1	2	2	1	1	2	3
3	2	1	2	2	1	1	2	2
3	2	2	1	2	1	2	3	2
3	1	1	2	2	1	1	1	2
2	1	1	2	2	1	2	2	2
3	1	1	2	3	1	2	1	2
3	1	1	2	2	1	1	2	3
3	1	1	2	2	1	1	1	2
3	1	1	2	3	1	1	1	3
3	1	2	3	3	1	1	1	2
2	1	1	3	2	1	2	2	3
3	2	1	3	2	1	1	2	3
3	1	1	2	2	1	1	1	3
3	2	3	3	1	2	2	3	2
3	1	1	2	2	1	1	1	3
3	1	1	2	3	1	1	1	3
3	1	2	2	2	1	1	1	2
3	1	1	3	2	1	1	2	3
3	1	1	3	2	1	2	2	2
3	1	3	3	3	1	2	1	2
3	2	2	1	2	1	1	1	2
3	2	1	1	1	2	1	1	2
3	1	1	3	3	1	1	1	2
3	3	2	3	2	1	2	2	1
3	1	2	3	1	1	2	1	2
2	2	1	2	2	3	1	1	2
3	2	2	3	2	1	1	1	2
1	1	1	2	3	1	1	1	3
3	2	1	2	2	1	1	1	3
3	1	1	2	2	1	1	2	2
3	2	3	3	3	1	1	1	2
2	3	1	2	2	1	2	1	2
3	2	2	3	2	1	2	2	2
3	1	1	2	2	1	1	2	2
3	1	3	2	2	1	1	1	2
2	1	1	2	2	1	1	2	3
3	1	1	2	2	1	1	1	3
3	1	1	3	3	1	1	1	3
3	1	1	3	3	1	1	1	3
3	1	1	3	2	1	1	2	3

3	2	2	2	1	1	1	1	1
3	1	2	2	1	1	1	1	2
3	2	1	3	3	1	1	1	3
3	1	2	3	2	1	1	2	2
3	1	1	2	3	1	1	1	2
3	1	1	3	3	999	1	2	3
3	2	1	2	2	1	2	3	2
3	3	3	3	3	3	3	3	3
3	1	1	2	2	1	2	1	1
3	1	1	2	3	1	1	1	3
3	1	2	1	3	1	2	1	2
3	1	1	3	3	1	2	1	2
2	1	2	2	1	1	2	1	2
3	1	1	2	2	1	1	1	2
3	1	1	3	3	1	1	1	3
3	2	1	2	3	1	1	1	3
2	1	2	3	2	1	999	2	2
3	1	1	2	2	1	1	1	2
3	2	1	3	3	1	2	1	3
3	2	2	3	2	1	3	1	3
3	1	1	2	2	1	1	1	2
3	3	1	2	999	1	1	1	2
3	2	1	2	2	1	1	1	2
3	2	1	2	1	1	2	1	2
3	2	2	3	2	1	2	3	2
3	1	2	2	1	1	1	1	2
3	2	1	2	2	1	1	2	2
3	3	2	3	3	1	2	3	2
3	1	1	2	2	1	2	3	2
3	2	3	3	3	1	3	2	3
3	2	1	3	3	1	2	3	2
3	1	1	3	2	1	2	1	2
3	2	1	2	1	2	1	3	2
3	1	1	2	3	1	1	3	2
3	1	1	3	1	2	1	1	3
3	999	1	3	2	1	1	2	2
3	1	1	2	2	1	1	3	2
3	3	2	2	2	1	3	3	1
3	2	1	3	2	1	2	1	3
3	1	2	1	2	1	1	3	2
2	1	1	1	2	1	1	2	2
3	1	1	2	2	2	2	1	2
3	1	1	3	2	2	2	1	1
3	2	2	2	3	1	2	2	2
3	2	1	2	2	1	2	3	2
3	1	1	2	3	2	1	1	3
3	3	3	2	1	1	2	2	2
3	1	1	2	2	1	3	1	3
2	1	1	2	2	2	1	1	2
3	1	2	2	2	1	999	1	2

2	1	1	2	1	1	2	3	2
3	1	1	3	3	1	1	2	2
3	1	1	2	3	1	1	3	2
3	1	1	2	2	3	2	2	2
3	1	1	2	2	1	2	1	2
3	1	1	3	3	1	1	1	3
3	1	1	2	3	1	1	1	2
3	1	2	3	3	1	2	1	3
3	1	1	3	3	1	1	2	3
3	1	2	3	2	1	2	1	2
3	2	2	3	2	2	3	2	2
3	1	1	3	3	1	1	3	1
3	2	3	2	1	1	3	2	1
3	3	2	3	2	1	1	2	2
3	2	1	2	2	1	1	1	2
3	2	2	2	2	1	1	2	2
3	1	1	2	2	1	1	2	2
3	1	1	3	3	1	1	1	3
3	2	1	3	2	1	2	1	3
3	1	3	3	2	1	3	2	1
3	2	2	3	1	1	2	1	2
3	2	1	3	1	1	2	2	1
3	1	1	3	2	1	1	1	3
3	1	1	3	2	1	1	2	2
3	1	1	3	2	1	2	1	3
3	1	1	3	2	1	1	1	2
3	1	2	3	2	1	1	2	2
3	1	1	3	3	1	1	1	3
3	2	2	3	2	1	1	2	1
3	1	1	3	2	1	2	2	3
3	2	3	3	2	2	3	3	2
3	1	1	2	2	1	1	2	2
3	2	2	1	1	2	2	1	2
2	1	1	2	2	1	1	2	2
2	1	1	2	2	1	1	2	2
3	1	1	2	2	1	2	2	2
2	1	1	2	2	1	1	1	2
3	1	1	3	2	1	1	2	2
3	1	1	2	2	1	1	2	2
3	2	1	3	3	1	1	2	2
3	2	2	3	2	1	3	2	3
3	1	1	3	3	1	1	2	2
3	1	1	3	3	1	1	2	3
3	3	2	3	2	1	1	2	2
3	1	1	2	2	1	1	2	2
3	1	1	2	2	1	1	2	2
3	1	1	3	2	1	1	1	2
3	1	1	3	2	1	1	3	999
3	1	1	3	2	1	1	1	3
3	1	1	3	2	1	1	1	3

3	1	1	2	2	1	2	2	2
3	2	1	2	3	1	1	1	2
3	1	1	3	2	1	1	1	1
2	1	1	2	2	1	1	3	1
3	1	1	2	2	1	999	1	2
3	1	1	2	1	2	2	2	1
1	1	1	2	2	1	1	1	2
3	1	1	3	1	1	1	2	2
3	1	2	3	2	1	1	2	2
3	1	1	3	2	1	1	1	2
3	3	2	1	2	2	3	1	2
3	1	1	2	3	1	1	3	2
3	1	1	3	3	1	2	2	3
3	1	2	3	3	1	2	1	3
3	1	1	3	1	1	2	1	2
3	1	1	2	2	1	2	1	3
3	1	1	2	2	1	1	1	2
3	1	1	2	3	1	1	1	2
3	1	1	3	2	1	1	1	3
3	1	1	2	2	1	1	1	2
3	1	2	3	2	1	3	1	2
3	2	1	3	3	1	2	2	2
3	1	2	3	3	1	2	1	3
3	2	2	1	1	1	1	1	1
3	1	1	2	2	1	1	1	1
3	1	2	3	3	1	2	1	3
3	1	1	2	2	1	1	1	2
3	1	1	2	3	1	2	1	2
3	1	1	2	3	1	1	1	2
3	1	1	3	3	1	1	1	3
3	2	2	2	2	2	1	2	2
3	1	1	3	2	1	1	1	2
3	1	1	2	2	1	1	1	2
3	1	1	2	3	1	1	2	2
3	1	1	2	2	1	2	2	2
3	1	1	3	3	1	1	2	1
3	2	1	3	2	1	2	1	3
3	1	2	3	2	1	2	2	3
3	2	1	2	2	1	1	1	3
3	1	1	3	2	2	1	1	2
3	1	1	2	2	1	1	2	2
3	1	1	2	2	1	1	2	2
3	1	1	2	2	1	2	1	3
3	1	1	3	3	1	1	1	3
3	1	2	2	2	1	3	2	1
3	1	1	3	2	1	1	1	2
3	1	1	2	2	1	2	1	2
3	1	1	2	3	1	2	1	2
3	1	1	3	2	2	2	1	3
999	999	999	999	999	999	999	999	999

3	2	2	3	2	1	2	3	3
3	1	2	3	2	1	3	3	2
3	1	1	2	1	1	2	2	1
3	2	1	3	3	1	1	2	2
3	2	2	3	2	1	3	2	2
3	1	1	2	2	2	1	3	2
3	1	1	2	2	2	1	1	2
3	1	2	3	1	1	1	2	2
3	1	2	2	2	1	1	2	2
3	2	1	2	2	1	2	3	2
1	1	1	3	1	1	2	1	3
3	2	1	2	2	1	1	2	2
3	1	1	2	1	1	1	3	2
3	1	2	3	2	1	2	3	2
3	2	2	3	3	2	2	3	3
3	1	1	2	2	1	2	1	2
3	1	1	3	2	1	1	1	2
3	1	1	2	2	2	1	3	1
3	2	3	3	2	1	3	2	2
3	1	2	3	3	1	2	2	3
3	2	1	2	1	1	3	1	2
3	1	1	2	3	1	1	3	3
3	3	2	1	2	1	2	2	2
3	1	1	3	3	1	1	1	1
3	1	1	2	2	1	1	3	2
3	1	1	3	3	1	2	1	2
3	1	1	2	3	1	2	1	3
3	2	2	2	2	1	2	2	2
3	1	2	3	2	1	2	2	2
1	1	2	2	3	1	1	2	1
2	1	1	2	1	1	1	2	2
3	2	1	2	2	1	1	2	2
3	1	1	2	3	1	2	2	3
3	1	1	2	2	1	2	2	2
3	1	1	2	3	1	2	2	2
3	1	2	3	999	1	3	1	2
1	1	1	2	2	1	2	1	2
3	1	1	3	1	1	3	3	3
3	1	1	2	2	1	1	2	1
3	1	2	3	3	1	2	1	3
3	1	1	3	2	1	1	2	2
3	1	2	3	2	1	2	1	2
3	2	1	2	2	1	1	1	2
3	1	2	2	3	1	1	2	3
3	1	1	2	2	2	1	1	2
3	1	1	2	3	1	1	1	3
3	1	1	2	2	1	1	1	3
3	1	1	3	3	2	2	2	3
3	1	1	3	2	1	1	3	2
3	1	1	2	2	1	1	2	2

3	1	1	2	3	1	1	1	2
3	1	1	2	2	1	1	1	2
3	2	3	2	3	1	2	3	1
3	1	2	1	2	1	2	1	2
3	1	1	2	3	2	3	2	3
3	1	1	3	3	1	3	1	3
3	1	1	1	2	1	1	2	2
3	1	1	2	999	1	1	2	2
3	1	2	2	2	1	2	2	1
3	2	2	3	2	1	3	1	2
3	1	1	2	2	1	1	3	2
3	1	1	2	2	1	3	3	2
3	1	1	2	2	1	2	1	2
3	1	1	3	3	1	1	2	3
3	1	2	1	1	1	1	2	3
3	1	1	3	3	1	2	2	3
3	1	1	3	3	1	1	2	2
3	1	1	2	2	1	1	2	2
3	2	1	1	3	1	2	1	3
3	1	2	2	2	1	2	2	2
3	2	999	3	2	1	2	3	2
3	1	1	2	2	1	1	2	2
3	2	1	2	1	1	1	2	2
3	2	1	3	2	1	1	2	2
3	2	1	2	1	1	1	2	1
3	1	1	2	2	1	1	2	2
3	3	1	3	2	1	1	3	2
3	1	1	2	2	1	1	2	2
3	1	1	2	3	1	3	1	3
3	1	1	2	2	1	1	2	1
3	3	1	2	2	1	1	1	1
3	1	2	2	2	1	1	2	2
2	2	2	3	2	1	2	2	3
3	2	2	2	2	2	3	2	2
3	1	1	3	2	1	1	2	2
3	1	1	3	2	2	1	1	2
3	1	1	2	2	1	1	2	2
2	2	1	2	2	1	1	1	2
3	2	1	3	2	1	1	1	2
3	1	1	3	2	1	1	1	2
3	1	1	3	3	1	2	1	3
3	1	1	2	2	1	2	1	3
2	1	2	2	2	1	1	2	3
3	1	1	2	2	1	3	1	2
3	1	1	2	2	1	2	2	3
2	1	1	2	2	1	2	1	2
3	1	1	3	3	1	1	1	3
3	1	1	3	2	1	2	1	2
3	2	2	3	1	1	3	2	2
3	1	1	2	3	1	2	1	2

3	1	999	2	3	1	2	1	2
3	1	1	2	2	1	2	2	3
3	1	1	2	2	1	2	1	3
3	1	1	3	2	1	2	2	3
3	2	1	2	3	1	1	2	3
3	1	1	3	2	1	2	1	2
3	1	1	2	1	1	1	3	1
3	1	2	1	1	1	1	1	1
3	2	1	3	2	1	2	2	2
3	2	1	1	2	1	1	1	3
3	1	1	2	2	1	1	1	2
3	1	1	2	2	1	2	2	2
3	999	1	2	1	1	1	1	2
3	1	1	3	2	1	2	1	2
3	1	1	2	2	1	2	1	3
3	1	1	2	2	1	1	2	3
3	1	1	2	2	1	1	1	3
3	1	1	3	3	1	1	1	2
3	1	1	3	2	2	1	1	2
2	3	2	2	2	3	1	1	2
1	2	1	2	1	1	2	1	2
3	1	2	2	2	1	2	1	2
3	2	1	2	2	1	1	1	2
3	1	1	3	2	1	3	1	3
3	1	1	2	2	1	1	1	2
3	1	1	2	3	1	1	2	3
3	2	1	2	2	1	1	1	2
2	1	1	1	2	999	1	2	2
2	1	2	1	2	1	1	2	2
3	1	1	2	2	1	3	1	2
1	2	1	3	1	1	1	1	3
3	1	1	3	3	1	2	2	3
3	2	3	3	2	2	1	2	2
3	1	1	3	3	1	1	2	3
3	1	2	2	2	1	2	2	2
3	1	2	3	2	1	2	3	2
3	1	1	2	2	1	2	2	2
3	1	1	2	2	2	1	1	1
3	1	1	2	2	1	1	2	2
2	1	1	2	2	2	1	3	2
3	1	1	3	3	1	2	1	3
3	999	1	3	2	1	3	2	2
3	1	2	2	2	1	1	2	2
3	1	1	2	2	1	2	2	3
3	1	1	2	2	2	1	2	2
3	1	2	3	3	1	2	3	2
3	1	2	2	3	1	1	2	3
3	2	2	2	3	1	2	1	3
3	1	1	2	3	1	2	2	3
3	1	1	3	2	1	1	3	2

3	2	3	3	2	1	3	3	2
3	1	1	2	2	1	2	2	2
3	1	1	2	2	1	1	1	2
3	1	1	2	3	1	2	1	2
3	2	1	2	2	1	1	1	3
3	2	2	3	3	2	3	2	2
3	1	1	2	2	1	2	1	2
3	1	3	3	2	1	2	2	2
3	1	2	2	2	1	2	3	2
3	1	1	2	2	1	1	2	3
3	1	1	3	2	1	2	2	2
3	1	2	3	3	1	1	1	1
3	1	1	2	3	1	3	2	3
3	1	1	3	2	1	2	2	2
3	2	1	3	2	1	1	3	2
3	1	1	3	3	1	2	2	3
3	2	2	2	3	1	2	1	2
3	1	2	3	2	1	3	1	2
3	1	2	2	3	1	1	2	2
3	2	3	2	2	1	1	2	2
3	2	1	1	1	1	2	1	2
3	1	1	3	2	1	2	1	2
3	1	1	2	1	1	1	1	2
3	1	1	2	2	1	1	1	2
3	1	1	3	3	1	1	2	2
3	1	1	2	3	1	3	1	2
3	2	2	3	2	1	2	2	3
2	1	1	2	2	1	1	1	2
3	1	1	3	3	1	2	1	2
3	1	1	2	3	1	2	1	3
3	1	1	3	2	1	2	1	2
3	2	3	3	3	2	3	3	2
3	1	1	3	3	1	1	1	1
3	1	1	3	3	1	2	1	3
3	1	1	2	1	2	1	2	2
2	1	1	2	3	1	1	1	3
3	1	1	2	2	1	2	1	3
3	2	1	3	2	1	2	1	2
3	1	2	2	2	1	2	2	2
3	1	1	3	3	1	2	1	2
3	1	1	2	3	1	1	1	3
3	1	1	2	2	1	1	1	3
3	1	1	1	1	1	1	2	2
3	1	1	3	3	1	1	1	3
2	1	2	3	2	1	2	2	2
3	1	1	2	2	2	2	1	1
3	2	1	2	2	1	2	1	1
2	1	1	2	3	1	1	1	3
3	1	1	2	3	1	2	1	3
3	1	1	3	2	1	1	1	3

3	1	1	2	2	1	3	1	2
3	1	2	3	2	1	2	1	2
3	1	1	2	2	1	1	1	2
3	1	1	2	3	1	1	1	2
3	1	1	2	3	1	999	2	2
3	1	1	3	2	1	1	1	2
3	1	1	3	2	2	1	1	2
3	2	2	2	2	1	1	1	2
3	1	2	3	2	1	2	2	2
3	2	3	3	3	1	2	3	2
3	2	2	2	2	1	1	1	2
3	2	2	2	2	1	1	1	2
2	2	3	2	2	2	1	2	2
2	1	1	2	2	1	1	1	3
2	2	2	3	1	1	2	2	2
3	3	3	3	1	3	1	1	2
3	2	1	2	2	2	2	2	1
3	1	2	2	3	1	1	1	3
3	2	2	2	2	1	1	3	2
3	2	2	3	2	1	2	2	1
3	1	1	3	1	2	1	999	3
3	1	1	2	3	1	1	1	2
3	2	1	3	3	1	2	1	3
3	2	2	2	2	1	1	2	3
3	2	1	2	2	1	2	1	2
3	1	1	2	2	1	1	2	2
3	1	2	3	3	1	1	1	3
2	1	2	2	2	2	2	1	2
2	2	1	1	1	1	2	1	1
3	1	1	2	3	1	1	1	3
3	1	1	3	3	1	1	1	3
3	2	2	3	2	1	2	2	2
2	1	1	2	2	1	2	1	2
3	2	1	2	2	1	1	3	2
2	1	3	3	2	1	1	2	3
3	1	2	3	2	1	2	1	2
3	2	2	2	3	1	2	1	2
3	1	3	3	2	1	2	1	2
3	1	2	1	2	1	2	1	1
3	2	2	2	2	1	2	3	2
3	1	2	3	2	1	1	2	3
3	1	2	3	3	1	1	1	3
3	2	1	2	2	1	1	2	2
3	3	2	3	3	1	3	2	2
3	2	2	3	2	1	2	2	3
3	2	3	3	1	1	1	2	3
3	1	1	3	3	1	1	1	3
3	2	2	3	2	1	2	1	2
3	1	1	3	2	1	2	1	2
3	1	1	2	3	1	1	1	3

3	2	1	2	2	1	1	1	3
3	1	1	2	2	1	1	3	2
2	1	1	2	2	1	2	1	1
3	2	2	3	3	1	2	1	2
3	1	1	2	3	1	1	1	2
2	2	1	3	1	2	2	2	2
3	2	3	3	1	3	3	1	1
3	3	2	3	2	1	2	2	3
3	1	1	3	3	2	1	2	3
3	2	3	2	3	1	2	3	1
3	2	2	3	3	1	1	1	2
3	1	1	2	1	1	2	1	3
3	1	1	2	2	1	1	3	3
3	1	2	2	3	1	2	1	3
3	1	1	3	3	2	2	1	2
3	1	1	3	3	1	1	1	2
2	1	1	2	3	1	1	2	3
3	1	1	3	3	1	1	2	2
3	1	1	2	3	1	1	1	2
3	2	1	2	3	1	1	1	3
3	1	1	2	3	1	1	1	3
3	3	1	2	1	3	1	1	1
3	1	2	3	3	1	1	1	3
3	1	1	1	2	1	1	1	2
1	1	1	2	2	1	1	1	2
3	1	1	3	3	1	1	1	2
3	1	1	1	2	1	1	1	3
3	2	2	3	3	1	2	1	3
3	2	1	3	3	1	3	2	3
3	1	1	3	3	1	1	1	3
3	1	1	2	2	1	1	1	2
3	3	1	3	3	1	1	3	2
3	3	1	3	2	1	3	1	3
3	2	1	2	2	1	1	1	2
3	3	2	1	2	1	2	2	2
3	1	1	2	2	1	2	1	2
3	1	1	2	3	1	2	1	3
3	1	1	2	2	1	2	1	2
3	1	2	3	2	3	2	1	3
3	1	1	2	2	999	2	1	3
3	999	1	3	3	1	1	1	3
3	1	1	2	2	2	2	1	1
2	1	1	2	2	1	1	1	3
3	2	3	3	3	1	1	1	3
3	1	2	2	2	1	1	1	2
3	1	1	3	3	1	2	2	3
2	1	1	2	2	1	2	1	1
3	1	1	2	2	1	2	1	2
3	2	3	3	2	2	1	1	2
3	1	1	2	2	1	1	1	3

3	999	2	2	2	1	3	1	2
2	1	2	2	1	1	1	1	1
3	2	1	1	2	2	2	1	3
3	1	2	3	2	2	2	1	2
2	1	2	3	2	1	2	3	2
3	2	1	2	1	1	2	1	2
2	2	3	3	1	3	2	1	1
3	1	1	2	3	1	1	2	2
3	1	2	3	3	1	2	1	2
3	1	1	3	2	1	1	2	2
3	2	1	2	2	1	1	1	2
3	2	2	2	2	1	2	1	2
3	2	3	2	2	2	2	2	2
2	1	1	3	2	1	2	3	2
1	2	2	3	2	3	1	1	3
3	2	1	3	1	1	1	3	3
3	2	2	3	2	1	1	2	2
3	1	1	2	2	1	2	1	3
3	3	1	2	1	1	1	1	3
2	2	1	2	2	1	1	1	2
3	2	1	2	1	1	1	1	1
3	3	2	3	1	1	3	1	2
3	3	2	2	2	1	2	1	3
3	1	2	3	1	1	1	1	1
3	2	2	2	1	1	1	1	2
2	1	1	2	2	1	1	1	2
3	1	1	3	2	1	2	1	2
3	1	1	3	2	1	1	2	3
3	2	1	2	2	1	1	1	3
3	2	2	3	2	1	1	1	2
3	1	1	3	2	2	1	1	2
3	3	2	3	3	1	1	3	2
3	1	1	3	2	1	1	1	2
3	2	1	2	2	1	2	2	3
3	1	1	2	2	2	2	1	2
3	1	1	2	2	1	1	1	2
1	1	1	2	2	1	1	1	2
3	2	1	3	3	1	2	2	2
2	1	1	3	3	1	1	1	3
3	3	1	2	2	1	3	2	2
3	1	1	3	2	1	1	1	2
3	1	1	2	2	1	1	1	2
1	1	2	2	3	1	2	1	3
3	2	1	2	2	1	1	1	2
3	1	1	2	2	1	3	1	3
3	1	3	3	2	1	2	2	2
3	1	1	2	3	1	2	3	2
3	3	1	3	2	1	3	2	1
3	1	1	3	2	1	2	1	2
2	2	1	3	3	1	1	1	2

3	2	2	2	1	3	2	1	2
2	1	1	2	3	1	1	1	3
3	1	1	3	3	1	1	1	3
3	1	2	3	2	1	2	1	2
3	2	1	2	2	3	2	1	2
2	2	2	2	2	1	1	1	2
1	2	2	3	2	1	2	3	1
3	1	2	1	3	1	1	2	2
3	1	1	2	2	1	1	1	2
3	3	3	2	2	1	2	1	2
1	3	1	1	3	1	1	1	2
3	1	2	2	2	1	1	1	3
3	2	1	2	3	1	2	1	2
3	1	1	2	2	1	1	1	2
3	999	1	3	2	1	3	1	2
3	999	1	3	3	1	999	1	3
3	1	1	3	2	1	1	1	3
3	1	1	2	2	1	1	1	2
3	2	1	3	3	1	2	2	3
3	2	2	2	1	2	1	1	2
3	3	1	3	2	3	2	1	2
3	2	2	2	2	1	1	1	2
3	1	1	2	2	1	3	1	2
3	1	1	3	3	1	2	1	3
3	1	1	2	2	1	2	1	2
3	1	1	2	3	1	1	1	2
3	1	1	3	2	1	2	1	2
3	2	1	3	3	1	1	2	2
3	1	1	2	2	1	1	1	2
3	1	1	2	2	1	1	1	3
3	1	1	3	3	1	1	1	1
3	1	1	3	999	3	1	1	3
3	1	1	2	2	1	1	1	2
3	1	2	3	3	1	3	1	2
3	1	1	3	1	1	2	1	2
3	1	1	2	3	1	2	1	2
3	2	1	3	1	1	3	2	2
3	1	1	3	2	1	2	1	2
3	2	1	1	2	2	1	1	2
2	2	1	3	2	1	3	1	2
3	1	1	2	2	1	1	1	2
3	2	2	2	2	1	2	1	2
3	2	1	1	1	1	1	1	2
3	1	2	3	3	1	1	1	2
3	1	1	2	1	2	1	2	2
3	1	1	2	2	2	2	1	2
3	1	3	3	2	1	2	3	2
1	2	1	2	2	3	3	2	2
3	2	2	3	2	1	2	1	2
3	1	2	2	3	1	1	2	1

3	1	1	2	1	1	2	1	2
3	1	1	3	3	1	2	1	2
3	1	1	3	2	1	2	1	2
2	2	1	2	2	1	1	1	2
2	1	1	2	3	1	1	1	2
3	999	1	3	2	1	1	1	2
3	2	1	2	2	1	2	1	2
2	3	1	2	2	1	1	3	1
3	2	2	3	3	1	2	1	2
3	2	2	2	2	3	1	1	2
3	2	1	2	2	1	2	2	3
3	1	1	3	3	1	1	2	3
3	1	2	1	2	1	1	1	2
3	2	2	3	2	1	3	2	3
3	2	1	2	2	1	1	1	2
3	1	1	2	2	1	1	2	2
3	1	1	3	3	1	2	2	2
3	1	1	3	3	1	1	1	3
3	2	2	3	2	1	1	1	2
2	3	1	2	2	3	2	1	2
3	1	1	3	2	2	3	3	2
3	1	1	2	3	1	2	2	3
3	1	1	2	2	1	1	1	2
3	3	1	1	1	2	1	2	2
3	2	1	2	999	2	2	1	2
3	1	1	1	2	1	1	1	2
3	2	1	2	3	1	1	1	2
1	1	1	1	1	2	1	1	1
3	1	2	3	3	1	2	1	3
3	2	1	3	1	1	1	1	2
3	1	3	2	1	1	1	2	2
3	1	1	3	2	1	3	2	2
3	1	1	3	3	1	2	1	3
2	2	1	2	2	1	2	1	2
3	2	1	2	2	1	2	2	2
3	2	1	2	2	1	1	1	2
3	1	2	2	1	1	3	2	2
2	2	1	2	2	1	1	1	1
3	1	1	1	3	1	2	1	2
3	2	1	2	2	1	1	2	2
3	2	1	3	2	1	1	1	2
3	3	1	2	2	3	1	1	2
2	3	1	1	1	2	1	1	1
3	1	2	3	3	1	2	1	3
3	2	3	2	2	1	2	2	3
3	2	1	2	1	1	1	1	3
3	1	1	2	2	1	2	2	2
3	1	1	2	1	2	1	1	1
3	2	1	3	2	1	2	1	3
3	1	1	2	2	1	1	1	1

3	2	1	2	3	1	2	2	3
3	1	1	3	3	1	2	1	2
1	1	1	3	3	1	3	1	2
3	1	1	2	3	1	2	1	1
3	2	1	3	2	1	2	1	3
3	1	1	2	3	2	3	1	3
2	2	1	3	2	1	1	1	3
2	1	2	2	1	1	2	1	2
3	2	1	1	2	1	3	1	1
3	2	1	1	2	1	1	1	2
3	2	1	1	2	1	1	1	1
3	1	1	2	3	1	1	1	2
3	1	1	3	3	1	1	1	3
3	1	1	2	2	1	1	1	3
2	999	1	3	2	1	1	2	3
3	2	1	3	3	1	2	1	3
3	2	2	3	2	2	2	1	2
3	2	1	2	3	1	1	1	2
3	1	1	2	3	1	2	999	2
3	1	1	2	2	2	2	1	2
3	1	2	2	3	1	2	1	2
3	1	1	2	2	1	1	1	1
3	1	1	3	3	1	1	1	3
1	2	1	1	2	2	2	1	2
3	1	1	2	2	1	2	1	3
3	1	1	1	2	1	2	1	2
2	2	1	1	2	1	1	1	2
3	1	2	2	2	1	2	1	2
3	1	1	2	3	1	2	1	3
2	2	1	2	3	1	2	1	2
3	1	2	3	3	2	2	1	2
3	2	2	3	2	1	1	3	2
3	1	2	3	2	1	2	1	2
2	1	1	2	2	1	2	1	2
3	2	2	3	2	2	2	1	2
3	2	2	3	2	1	2	1	1
3	1	1	2	3	2	2	1	2
2	1	1	2	2	2	2	1	2
1	3	3	2	1	1	3	1	1
3	3	2	3	2	1	2	2	3
3	3	1	3	3	1	3	3	3
3	2	1	2	1	1	1	2	3
2	1	2	2	999	999	1	1	2
3	1	1	2	2	1	1	1	3
3	1	1	2	2	1	1	2	2
3	3	2	2	2	1	2	2	3
1	3	2	3	3	1	3	3	1
3	1	1	3	2	1	2	1	3
3	1	2	3	3	1	2	2	3
3	2	1	3	3	1	1	1	2

3	2	2	3	2	1	1	2	3
3	1	1	3	2	1	1	1	2
3	2	2	2	1	1	3	1	2
3	1	1	3	3	1	1	1	3
3	2	2	3	3	1	2	1	2
3	1	1	3	3	1	2	1	2
3	1	1	2	2	1	1	1	1
3	1	1	2	2	2	2	1	3
3	1	1	2	3	3	3	2	2
3	2	1	2	3	1	2	1	1
3	2	2	2	2	1	3	2	2
2	1	1	1	2	1	1	2	2
3	1	1	3	3	1	2	1	3
3	1	1	2	2	1	2	1	2
3	1	1	2	3	1	2	1	3
3	2	2	1	2	3	1	1	3
3	1	2	3	2	1	2	1	2
3	1	1	3	2	1	1	2	3
3	1	1	2	2	1	1	2	2
2	2	2	3	2	1	1	1	2
2	1	1	1	2	1	1	1	2
3	1	1	2	3	1	2	1	2
3	1	2	1	2	2	1	1	1
3	2	1	2	2	2	3	1	1
3	1	1	2	2	1	1	1	2
3	1	1	2	2	1	2	1	3
2	2	1	1	2	1	1	1	1
3	1	2	2	2	2	1	1	2
3	1	2	2	2	1	1	1	2
3	2	1	3	2	1	2	1	2
3	1	1	3	3	1	3	1	3
3	2	1	2	2	1	2	1	3
3	1	1	3	3	1	2	2	3
3	999	1	2	2	1	1	1	2
3	2	1	1	2	1	1	1	2
1	1	1	2	2	1	1	2	2
3	1	1	3	3	1	1	1	2
2	1	1	1	2	1	1	1	2
2	1	1	1	2	1	1	2	1
3	1	1	2	2	1	1	1	3
3	1	1	2	2	1	2	1	2
3	3	1	1	2	2	2	1	3
3	999	2	1	2	1	1	1	3
3	2	2	3	2	1	2	1	2
3	2	2	3	1	1	3	1	3
2	1	1	2	3	1	1	1	3
3	1	2	1	2	1	1	1	2
3	2	1	1	1	3	1	2	2
3	2	1	2	3	2	1	1	2
2	3	1	2	3	1	1	1	1

3	1	1	2	2	1	2	1	2
3	2	1	2	3	1	2	1	3
3	1	1	2	3	1	2	1	2
3	1	1	2	3	2	2	1	3
3	1	1	2	3	1	1	1	3
3	1	1	2	2	1	3	1	3
3	2	2	3	3	1	2	1	2
3	1	1	3	2	1	2	2	3
3	2	1	2	1	1	1	1	2
3	3	1	1	1	3	2	1	3
3	1	1	3	2	2	2	1	3
3	2	2	3	3	1	1	3	3
3	1	1	2	3	1	1	1	2
3	2	1	3	2	2	2	1	2
3	1	1	1	3	1	1	1	2
3	1	1	2	2	1	1	2	2

BES1	BES2	BES3	BES4	BES5	BES6	BES7	BES8	BES9
1	5	5	5	5	4	1	1	3
1	2	5	5	4	3	3	1	3
1	4	5	5	5	1	5	1	5
1	5	5	4	5	5	1	1	4
1	2	4	5	4	2	1	1	5
3	5	5	2	5	3	2	1	5
999	999	999	999	999	999	999	999	999
1	1	5	1	999	1	5	1	5
4	2	5	5	4	5	1	1	4
5	5	5	5	5	3	1	1	5
1	5	5	5	2	3	1	1	4
2	5	5	2	1	2	2	1	4
999	2	999	3	4	4	2	2	2
1	5	4	5	4	3	1	1	3
2	4	5	5	4	2	1	1	3
2	3	4	5	2	1	4	2	3
2	5	5	2	4	3	1	1	3
3	3	5	5	4	3	3	1	4
3	2	4	3	2	3	3	4	3
999	999	999	999	999	999	999	999	999
1	4	5	5	5	1	1	1	5
5	4	5	5	5	3	1	1	5
1	4	5	4	5	2	1	1	3
1	4	4	3	2	3	4	1	3
1	1	5	5	4	1	2	1	1
4	4	4	999	5	2	4	4	4
2	4	5	2	4	2	2	2	5
1	5	5	5	5	2	5	1	5
2	3	4	5	4	2	3	1	5
4	3	4	5	4	1	3	1	4
5	1	5	5	5	1	4	1	5
999	4	4	3	3	3	5	3	999
4	4	4	4	3	4	3	3	3
1	3	5	5	4	3	2	1	4
2	3	4	5	4	2	2	1	4
2	2	2	5	4	4	4	4	4
2	3	3	4	4	4	2	1	1
1	4	5	999	4	1	3	1	5
2	4	4	5	4	2	4	2	4
1	5	5	5	4	3	1	1	5
999	999	999	999	999	999	999	999	999
999	999	999	999	999	999	999	999	999
3	1	4	2	3	3	2	2	2
2	4	5	1	3	4	5	1	3
999	999	999	999	999	999	999	999	999
2	4	5	5	3	2	1	1	5
5	5	999	5	5	1	5	1	5
3	4	4	5	4	2	2	2	3
2	5	4	5	4	3	1	1	3

2	3	4	5	4	1	1	1	5
999	2	5	1	4	4	4	2	4
2	1	4	4	5	3	1	3	5
1	4	5	2	4	1	1	1	5
2	2	4	5	5	2	1	1	5
4	5	5	5	4	3	1	5	5
4	4	5	1	4	1	1	1	5
3	5	5	3	4	3	1	1	5
2	3	4	2	4	3	2	1	3
4	2	4	5	4	4	2	2	2
1	3	5	4	5	5	4	1	5
1	4	5	3	3	1	1	1	4
5	1	2	5	1	1	1	1	1
1	4	5	999	2	2	2	1	2
1	5	5	4	5	1	1	1	5
2	4	4	3	4	3	1	1	4
4	4	5	5	4	2	1	1	3
1	4	5	4	4	3	1	1	4
999	999	999	999	999	999	999	999	999
999	999	999	999	999	999	999	999	999
3	4	5	5	5	3	2	1	5
1	5	5	4	5	1	1	1	5
999	999	999	999	999	999	999	999	999
3	1	5	5	1	3	3	1	1
2	2	999	999	999	999	999	999	999
999	999	999	999	999	999	999	999	999
999	999	999	999	999	999	999	999	999
4	3	4	2	4	3	2	2	2
1	5	5	5	5	2	1	1	5
4	3	5	1	4	5	5	1	5
2	5	5	5	3	3	1	1	1
2	4	4	1	3	3	4	1	3
1	5	5	1	5	5	1	1	5
1	2	5	4	3	3	1	1	1
1	4	4	1	3	1	2	1	5
1	5	5	1	5	1	1	1	5
4	4	5	5	3	3	2	1	4
3	2	5	3	3	3	1	1	2
1	2	5	3	999	2	1	1	5
1	5	5	3	4	1	1	1	4
5	5	5	5	4	3	1	1	5
3	2	4	4	3	3	5	2	4
1	5	4	5	3	4	1	3	4
999	999	999	2	3	4	999	999	999
5	3	5	2	4	2	5	1	2
2	4	4	5	3	4	2	1	3
5	5	5	5	4	1	1	1	4
2	4	5	2	3	3	3	1	3
5	2	5	3	3	4	1	1	4
1	4	4	4	4	2	1	1	4

1	2	5	1	2	1	5	3	5
2	4	5	1	4	1	1	1	5
2	2	5	4	4	3	2	1	5
1	4	5	5	5	1	1	1	5
2	5	5	5	5	2	2	1	5
4	5	5	5	3	5	5	2	5
1	5	4	5	4	3	1	1	4
5	5	5	5	5	5	1	1	5
5	3	3	3	5	3	3	4	3
5	1	5	3	5	999	1	1	5
4	2	4	4	4	4	3	2	3
1	2	4	5	4	2	2	1	4
1	5	5	1	1	1	5	5	1
5	1	5	3	999	5	5	3	1
4	3	5	3	5	4	2	1	5
5	4	5	1	4	2	2	1	3
1	5	5	5	4	3	2	1	3
1	4	4	1	3	1	1	1	4
1	5	5	5	5	1	5	1	5
4	5	5	5	4	3	4	1	4
1	4	4	5	4	2	1	1	4
3	4	5	5	4	3	4	2	4
1	4	5	4	5	1	1	1	5
3	3	4	5	4	2	3	3	4
3	999	2	3	4	2	1	2	4
1	2	5	2	4	1	1	1	3
2	4	5	4	4	3	4	2	4
1	5	5	5	4	1	1	1	5
2	3	3	5	2	3	2	1	3
4	3	3	4	2	3	5	3	5
3	2	4	4	3	3	4	1	5
1	3	5	5	4	4	1	1	5
4	4	3	5	4	3	1	2	2
3	3	3	2	3	2	2	3	2
2	1	1	3	2	2	3	1	2
3	2	3	5	2	3	4	1	3
1	4	4	5	3	3	1	1	4
1	5	5	5	5	2	1	1	5
1	4	4	3	5	1	1	1	4
2	3	4	1	3	3	2	2	4
4	3	5	5	4	3	2	1	4
1	5	5	4	4	1	1	1	3
1	5	4	2	4	2	2	2	3
1	3	5	5	4	3	1	1	4
3	2	4	5	3	3	4	4	4
3	2	4	4	4	2	2	1	4
1	5	5	5	5	4	1	1	5
2	3	5	5	4	3	2	1	5
2	4	4	5	4	3	1	1	4
5	4	999	2	5	1	4	1	4

3	2	5	4	3	4	3	1	5
1	5	5	5	4	4	1	1	4
2	1	4	5	1	2	1	1	4
1	1	5	5	5	2	1	1	5
2	5	5	5	4	1	1	1	1
1	4	5	5	5	1	1	1	5
1	4	5	3	3	1	2	1	4
1	5	5	4	5	2	1	1	5
2	3	5	5	4	3	3	1	1
4	3	5	4	3	4	2	1	3
1	4	5	5	4	3	2	1	5
1	5	5	5	4	1	1	1	3
2	3	4	1	2	1	5	1	5
2	4	4	5	3	3	1	1	4
1	3	4	3	3	3	3	1	3
1	4	4	3	4	3	2	1	3
2	3	5	4	5	2	3	3	4
1	4	5	5	4	1	1	1	4
1	4	4	5	3	1	1	1	3
3	3	5	5	4	2	2	2	4
2	5	4	4	4	1	1	1	4
1	2	5	3	5	1	1	1	5
1	5	5	4	5	2	1	1	4
3	4	5	3	4	2	1	1	1
3	5	5	5	5	1	1	1	2
1	4	5	5	3	2	1	1	3
1	4	5	5	5	1	1	1	4
2	4	5	5	4	3	1	1	4
5	5	4	5	4	3	1	1	5
1	2	5	5	5	2	1	1	5
2	4	4	4	3	2	2	1	4
1	2	4	5	3	3	2	1	4
1	4	5	1	2	3	1	1	4
4	5	4	5	2	4	2	2	3
3	5	5	5	4	4	1	1	3
2	3	4	5	3	3	1	1	5
5	4	4	1	3	4	4	1	4
1	4	5	1	4	2	1	1	5
1	4	5	1	4	3	1	1	3
5	5	5	5	5	5	5	4	5
1	3	4	4	3	4	3	2	4
1	2	5	4	3	2	4	1	4
2	4	4	5	5	2	2	1	4
2	4	5	4	4	2	2	2	4
2	4	4	5	5	3	1	2	3
1	5	5	1	5	3	5	1	5
1	2	5	4	4	2	2	1	4
1	4	4	5	2	3	3	4	3
1	4	4	5	3	3	1	1	3
1	999	5	3	4	2	2	2	5

1	3	5	5	3	4	1	1	4
2	3	1	5	1	1	1	1	1
1	5	5	5	5	1	1	1	5
1	5	5	4	5	2	1	1	5
1	5	5	4	5	1	1	1	5
1	4	5	5	5	2	1	1	5
2	5	5	5	4	3	1	1	4
5	5	5	5	5	1	1	1	5
1	4	5	4	5	4	1	1	2
3	1	5	4	3	4	5	1	3
4	1	2	5	2	5	5	3	2
1	1	5	5	4	3	1	3	5
2	5	4	5	4	3	1	1	4
2	4	5	5	4	2	2	2	5
1	1	5	4	5	1	3	1	5
1	4	4	3	3	2	2	1	4
1	4	5	5	5	1	4	1	2
3	4	5	1	3	4	3	1	4
2	2	5	5	4	1	5	1	4
3	4	3	5	2	1	2	3	4
2	1	4	1	1	1	5	4	5
1	4	5	4	4	2	2	3	4
5	4	5	5	4	2	1	1	4
3	2	3	5	4	3	2	3	4
3	3	4	2	4	4	2	1	4
1	4	5	5	4	1	2	1	4
4	4	5	3	4	3	2	1	3
2	5	5	1	3	4	2	1	4
1	2	4	2	4	4	1	1	2
3	3	5	2	2	999	4	2	2
2	3	4	4	4	3	2	1	5
3	4	5	4	4	2	2	1	5
4	4	5	4	3	2	2	1	4
2	3	4	3	3	2	999	3	3
4	4	5	4	4	3	2	1	4
1	3	5	5	5	2	1	1	5
2	3	5	5	4	3	1	1	5
2	3	4	5	3	2	2	2	4
1	4	4	2	4	3	1	1	4
2	3	5	1	5	3	1	1	3
1	3	5	4	4	3	2	1	4
1	4	4	5	3	1	1	3	3
3	4	5	5	4	4	1	1	5
1	4	5	5	4	2	2	1	4
3	4	5	5	3	4	1	1	4
1	5	4	2	4	2	1	1	5
4	3	4	5	4	2	2	1	3
1	5	5	5	4	2	1	1	5
1	4	4	5	5	2	1	1	4
999	999	999	999	999	999	999	999	999

2	5	4	5	5	2	1	1	5
3	3	4	5	4	3	2	1	3
5	5	5	5	2	1	1	1	5
2	3	5	5	3	2	4	1	4
2	1	3	5	3	4	1	2	3
1	5	5	5	4	1	1	1	2
2	3	4	2	4	2	2	1	5
1	5	4	5	5	1	1	1	4
3	5	4	4	3	3	1	1	3
4	3	5	3	2	2	2	2	4
3	4	5	1	4	2	5	2	5
1	5	5	4	3	3	1	1	4
2	3	4	5	5	4	1	2	4
1	5	5	5	5	4	1	1	5
1	4	5	5	5	3	1	1	5
5	4	4	4	5	2	2	1	4
1	4	4	5	5	1	1	1	4
2	4	5	5	4	3	2	1	4
1	4	2	5	4	4	1	1	4
2	5	5	5	2	4	1	2	3
3	4	5	2	1	2	3	1	3
2	4	5	4	3	1	1	1	3
1	2	1	1	2	1	1	2	5
4	4	4	1	4	1	4	4	4
1	5	5	3	1	3	1	1	1
5	4	5	2	5	1	4	1	4
1	5	5	3	3	3	1	1	5
2	1	5	5	5	3	2	1	5
4	4	5	5	4	4	1	1	5
1	5	5	2	5	1	1	1	5
1	4	5	4	4	3	3	2	4
4	3	4	2	3	4	1	3	1
2	2	5	5	3	2	1	1	1
3	4	5	5	4	3	2	1	4
2	2	5	5	2	2	4	1	3
1	5	5	5	4	999	4	2	4
2	4	4	3	4	999	2	1	4
2	1	5	5	1	1	2	3	2
1	4	5	5	4	3	1	1	3
2	4	5	4	5	4	1	1	4
1	4	5	4	4	4	1	1	3
2	4	4	5	4	3	1	2	4
3	3	3	4	2	3	3	1	3
2	4	4	5	4	2	2	1	3
3	3	3	4	3	3	2	1	3
1	2	4	5	3	3	2	1	3
1	5	5	3	5	5	3	1	4
3	3	1	3	4	1	1	1	4
1	2	5	5	5	1	1	1	5
2	4	5	5	3	3	2	2	4

1	4	5	2	4	5	1	1	4
1	2	5	5	5	4	2	1	5
2	2	5	4	4	1	1	1	5
2	4	4	4	3	2	1	3	4
1	2	5	5	5	1	1	1	5
2	2	1	3	2	2	1	1	2
1	4	5	4	4	3	1	1	4
1	4	5	5	4	2	2	3	5
1	1	5	3	4	2	1	3	4
1	5	5	4	5	1	1	1	5
4	5	5	5	3	4	4	1	4
1	2	5	1	2	2	1	1	5
1	2	5	4	5	1	2	1	5
1	5	5	5	4	2	1	1	5
1	5	5	5	4	1	1	1	5
1	4	5	5	5	1	4	1	4
1	4	5	5	5	1	4	1	5
1	4	4	2	3	2	1	2	3
3	3	4	5	4	5	1	4	4
3	3	3	3	4	2	2	1	3
4	3	3	5	2	4	1	1	2
1	5	5	4	5	4	1	1	2
1	4	5	2	3	4	3	2	5
4	3	4	2	3	4	1	4	3
1	2	5	5	4	2	1	1	5
2	4	4	5	4	3	1	1	3
4	4	5	3	4	3	1	1	4
3	3	4	5	4	3	2	2	3
2	3	5	2	4	2	3	2	4
2	1	4	5	4	2	2	1	5
3	2	1	4	3	2	2	2	2
1	4	5	4	3	3	2	1	4
2	4	5	5	4	1	1	1	5
5	4	5	999	3	4	4	4	4
1	4	4	2	4	4	3	2	4
1	2	5	5	4	4	1	1	4
4	4	4	4	4	3	2	1	4
1	2	2	999	3	4	4	3	4
3	4	5	4	3	3	3	2	3
1	5	4	5	5	2	1	1	5
1	4	5	5	4	3	1	1	4
1	4	5	3	4	1	1	1	5
2	4	4	3	5	3	1	1	3
2	4	3	5	4	3	1	1	5
1	5	5	5	4	4	1	2	5
2	1	5	2	3	2	2	3	3
1	5	5	3	5	1	1	1	5
1	2	5	4	5	3	1	1	5
3	3	5	4	4	2	3	2	4
2	2	5	1	5	1	2	1	4

1	4	5	4	5	2	1	1	4
1	4	5	4	1	3	3	2	5
4	3	5	2	2	3	4	1	4
4	2	5	5	4	5	1	1	5
4	4	4	5	4	3	2	2	4
2	5	4	5	4	3	1	1	5
2	5	5	2	5	1	2	2	5
1	4	5	4	5	3	2	1	5
3	4	4	1	3	3	2	2	4
1	4	5	5	999	1	1	999	4
1	1	5	5	5	5	1	1	1
2	4	3	5	3	3	2	1	3
1	1	4	2	3	3	2	4	3
1	2	4	1	3	2	1	1	4
2	3	5	3	4	1	2	3	4
3	4	5	5	4	3	1	1	5
1	4	5	1	4	1	1	1	2
5	5	5	5	999	5	1	1	3
1	5	5	5	5	4	1	1	5
2	3	5	1	4	2	2	2	5
4	5	4	4	5	3	2	2	3
2	2	4	3	3	2	4	1	4
3	1	2	2	4	3	3	1	4
1	4	5	4	5	2	2	1	4
1	5	5	5	5	4	1	1	4
1	4	5	5	4	3	1	1	3
4	2	5	5	4	3	2	4	3
2	4	4	5	3	4	3	1	3
4	3	5	3	4	4	4	1	5
1	3	5	5	3	4	3	1	3
1	4	3	5	4	1	3	2	3
1	4	5	2	4	999	999	999	999
2	4	5	5	3	3	1	1	3
1	3	4	4	4	3	2	1	4
2	5	4	3	5	2	1	1	4
2	4	5	5	4	3	1	1	1
1	5	4	3	4	2	1	1	5
3	4	5	5	3	4	3	2	5
1	4	5	5	4	3	1	1	4
2	4	5	5	3	2	1	1	5
1	2	5	4	4	2	2	1	2
2	3	5	5	4	4	1	1	4
1	4	5	3	4	2	1	5	4
1	4	5	5	4	2	1	1	4
2	4	3	4	3	3	1	1	3
1	5	5	5	4	2	1	1	5
4	4	4	4	4	3	2	1	5
2	4	5	5	4	2	2	1	3
1	5	5	5	4	4	1	1	4
1	4	5	5	4	2	2	1	5

2	4	5	4	2	1	1	3	5
1	2	5	4	4	2	4	4	4
5	4	5	5	3	4	1	3	5
4	3	4	4	4	4	3	3	4
3	3	5	4	3	3	3	2	4
4	3	5	5	4	2	4	1	2
999	999	999	999	999	999	999	999	999
2	4	5	1	3	2	3	1	4
4	3	4	3	3	1	1	4	4
1	4	5	1	4	1	1	1	5
2	4	5	5	4	4	1	5	1
2	3	5	3	5	1	5	1	5
1	2	5	4	3	2	2	1	5
1	5	5	5	4	4	2	1	5
1	5	5	5	5	1	1	1	4
2	5	5	5	4	4	1	1	4
2	2	5	4	4	1	5	1	4
1	4	4	4	5	4	1	1	5
1	4	4	4	4	1	5	1	5
2	4	5	3	3	3	1	1	5
3	2	5	5	5	2	2	3	2
4	4	5	3	4	2	3	2	4
1	4	5	4	4	3	1	1	4
1	4	5	2	4	4	2	1	4
1	4	4	5	5	1	1	1	5
1	4	4	4	4	3	2	2	3
2	2	4	5	4	3	2	1	4
1	5	4	4	4	2	2	1	4
1	3	5	3	4	3	2	1	3
1	5	5	5	4	2	3	1	4
1	1	1	4	1	1	3	2	5
1	2	5	5	5	999	4	1	1
999	999	999	999	999	999	999	999	999
1	1	5	2	5	2	2	1	4
2	2	4	5	5	2	2	1	5
3	3	4	3	3	4	2	1	4
1	5	5	5	5	1	1	1	5
2	2	5	4	3	3	3	1	3
1	4	5	5	4	1	1	1	4
1	3	4	5	4	2	1	1	4
1	5	5	5	5	2	1	1	4
1	3	5	4	3	2	1	1	3
3	5	4	5	3	2	999	3	4
3	5	4	3	4	4	1	1	5
2	3	3	4	3	3	2	2	3
4	3	5	4	4	2	2	2	4
3	5	999	1	4	2	1	3	5
3	3	5	5	4	2	3	2	4
1	3	5	4	3	2	2	1	3
1	5	1	4	5	1	2	1	5

1	2	5	5	5	3	3	2	4
2	4	5	5	5	1	5	1	5
2	3	5	4	4	2	4	1	4
1	5	5	5	4	1	1	1	4
1	3	5	4	4	1	1	1	5
1	4	5	3	4	2	2	1	4
1	3	1	5	2	1	3	1	1
3	3	4	5	4	3	4	1	4
2	4	4	3	4	1	2	1	4
3	2	4	3	3	3	3	3	3
2	3	2	4	3	5	3	4	2
5	4	3	1	3	3	4	2	3
1	4	5	4	4	3	2	2	4
1	3	5	4	3	2	3	999	3
4	5	5	5	4	3	1	1	4
4	3	1	2	3	4	1	2	1
3	3	4	4	3	4	1	1	4
999	999	999	999	999	999	999	999	999
1	3	4	5	1	4	1	1	4
5	2	3	4	1	3	2	4	2
2	2	5	5	5	2	3	2	4
2	1	3	1	2	2	2	3	3
2	1	5	2	1	4	3	1	3
2	3	4	5	4	3	2	1	2
1	1	2	1	1	1	1	1	3
2	3	4	5	4	4	2	1	4
2	4	5	2	4	1	3	2	5
5	2	5	3	4	3	4	999	5
2	1	3	2	1	1	2	1	2
1	5	5	2	3	4	1	1	5
1	1	5	1	5	1	1	1	5
2	2	4	3	2	1	1	3	2
2	4	5	5	3	1	1	1	3
2	3	4	2	4	2	4	5	4
1	2	3	2	3	4	2	3	2
4	3	2	3	2	2	2	3	2
1	5	5	5	5	1	5	1	5
2	3	4	3	4	4	3	2	4
999	999	999	999	999	999	999	999	999
999	999	999	999	999	999	999	999	999
999	999	999	999	999	999	999	1	999
2	2	5	5	2	5	5	2	4
2	2	4	999	999	999	999	999	999
5	4	3	4	5	5	5	4	4
5	4	5	5	5	5	4	1	5
1	5	5	5	3	4	1	1	5
1	5	5	1	3	2	1	1	5
2	4	4	2	4	2	2	1	4
3	4	5	5	5	4	3	3	3
5	1	1	1	1	2	1	5	5

3	3	4	4	3	1	3	2	3
3	1	2	5	5	3	2	1	5
4	4	4	5	3	2	4	4	2
2	5	5	5	5	2	1	1	5
3	4	5	4	2	2	4	1	3
2	2	4	4	1	2	1	2	2
3	1	5	1	2	1	1	1	3
1	5	4	2	5	1	2	1	4
1	2	4	1	5	4	2	2	5
3	2	4	3	4	3	5	1	3
2	4	3	3	4	4	3	5	1
1	3	5	4	3	2	2	1	4
999	999	999	999	999	999	999	999	999
999	999	999	999	999	999	999	999	999
3	4	5	3	4	3	4	2	4
1	4	5	2	4	1	2	1	4
4	2	5	5	4	1	2	1	4
3	2	5	3	4	2	4	2	3
1	5	5	5	5	1	2	2	4
4	3	5	2	5	1	1	1	4
1	2	5	2	5	4	1	1	5
999	999	999	999	999	999	999	999	999
1	4	5	2	4	1	2	1	5
2	3	4	5	3	4	2	3	2
3	4	5	5	1	1	2	3	5
1	5	5	2	5	1	1	1	5
3	1	5	1	4	1	2	2	4
4	4	5	1	5	4	5	1	5
1	5	5	1	4	4	4	1	5
2	2	5	4	4	1	2	1	4
2	3	5	5	5	2	2	1	5
1	2	5	4	1	1	3	1	5
5	3	5	5	4	1	2	1	3
3	2	3	4	3	4	3	2	2
3	3	5	2	5	5	999	2	2
999	3	3	999	1	3	2	4	2
2	1	4	5	3	1	4	3	3
3	2	4	5	3	4	3	2	3
3	2	5	4	2	2	1	1	5
1	2	3	3	2	1	1	1	1
1	4	4	1	5	1	1	1	5
3	1	4	2	2	3	1	3	3
2	4	4	1	5	4	2	1	4
3	3	4	5	5	3	3	1	3
2	2	4	4	3	3	3	2	3
3	3	5	5	4	4	3	3	4
2	4	4	3	4	3	3	2	4
4	3	4	2	4	4	1	2	4
3	3	4	3	3	3	2	3	4
4	2	3	5	4	1	1	1	5

5	3	4	1	4	3	4	2	4
2	999	4	4	3	4	2	2	3
4	1	3	4	3	2	5	3	4
1	5	5	1	5	4	1	1	4
2	1	3	3	3	3	3	3	3
2	3	4	1	4	3	4	1	4
1	3	5	5	3	1	1	3	3
2	2	5	4	4	3	1	1	4
2	3	4	5	4	2	2	1	4
1	5	4	4	5	1	1	1	2
3	1	4	5	3	999	4	2	3
1	2	5	2	4	3	3	2	5
2	2	5	3	2	5	4	3	3
1	4	3	5	3	2	1	1	5
1	2	5	1	2	1	1	5	3
1	4	5	5	5	1	1	1	5
2	1	5	5	5	2	1	1	2
2	4	4	3	4	3	2	2	5
2	4	5	3	4	2	2	1	4
3	4	5	4	4	4	4	2	5
4	3	4	4	4	4	4	1	3
2	2	3	5	3	3	1	3	4
3	3	3	5	4	2	2	1	3
4	2	5	4	2	5	5	4	5
3	4	5	2	4	1	5	1	5
4	2	4	2	4	4	4	2	4
1	4	5	1	4	1	4	1	4
3	4	5	3	4	2	1	1	5
1	4	5	3	4	3	4	1	5
1	3	4	1	4	3	4	1	5
5	5	5	3	5	5	1	1	3
2	5	5	5	4	3	1	1	4
1	4	5	3	5	2	2	1	4
1	2	4	5	3	3	2	1	4
3	2	3	3	3	2	3	1	3
3	2	4	5	3	2	1	2	3
3	1	5	3	4	4	2	2	2
1	3	4	5	5	2	2	1	4
2	3	2	1	4	2	1	2	2
3	3	4	4	3	3	2	1	4
3	4	5	5	5	4	2	2	4
2	3	4	5	5	3	4	2	3
1	1	4	1	4	4	4	1	3
3	4	5	2	4	2	1	1	4
4	4	5	4	3	4	1	1	4
2	3	1	2	4	2	2	1	4
4	5	4	5	2	3	4	3	5
2	1	3	5	3	5	5	5	3
2	4	5	3	4	4	1	1	4
2	3	5	1	4	1	2	1	4

1	4	4	5	4	2	2	1	2
2	1	4	3	4	2	5	2	5
2	4	5	1	4	4	1	1	4
2	2	4	4	4	3	4	2	4
3	4	3	4	3	3	3	3	3
1	2	5	5	5	1	5	3	5
1	2	1	2	1	2	4	4	3
2	1	5	5	3	1	1	1	5
2	5	4	5	4	1	1	2	5
1	4	5	4	3	1	2	1	5
1	3	5	1	5	1	3	1	3
2	5	5	1	4	2	1	1	4
2	2	3	3	3	3	4	3	2
3	2	4	2	3	2	3	1	2
1	4	5	5	5	1	1	1	5
4	3	5	2	4	2	2	3	5
1	4	5	3	3	4	3	1	4
1	2	5	2	4	2	2	1	1
1	5	4	4	3	3	5	1	3
1	2	3	3	3	3	3	3	5
1	1	5	1	1	1	2	1	5
3	2	4	4	4	3	3	2	3
2	2	4	4	3	2	3	4	1
2	2	4	3	4	2	2	3	4
1	5	5	4	4	4	1	1	5
2	2	3	5	3	3	3	1	4
3	2	4	3	4	2	1	2	3
2	4	5	4	4	2	2	1	4
2	3	5	3	4	3	2	1	3
2	4	5	5	4	2	1	1	4
1	4	5	1	5	1	1	1	5
2	3	4	3	2	3	2	1	2
3	2	4	3	3	2	3	2	3
4	4	5	1	5	4	1	1	5
1	5	5	5	5	1	1	1	5
1	4	5	5	4	2	3	1	4
1	4	5	5	4	1	1	1	5
2	3	4	2	4	4	1	1	4
2	1	4	1	3	2	2	2	4
3	4	5	1	3	1	1	3	5
2	3	5	5	5	5	4	3	4
1	2	4	1	4	4	5	1	4
2	4	4	5	2	4	2	1	4
2	2	5	1	4	2	999	1	5
1	4	4	2	3	3	3	3	3
2	4	2	3	2	3	2	3	2
2	5	5	4	4	3	3	4	4
1	3	4	5	2	3	4	2	3
2	4	3	2	3	2	2	1	4
2	5	5	5	4	3	2	1	3

5	4	4	2	3	1	2	3	3
3	4	4	3	3	5	3	1	3
3	2	5	2	3	3	2	1	2
2	2	4	1	5	3	3	3	5
2	2	4	2	3	3	2	1	4
4	4	4	4	4	4	4	4	3
5	4	4	3	4	3	1	5	4
3	3	4	4	3	3	2	2	2
4	3	5	5	4	4	1	1	3
1	3	4	1	2	3	2	1	3
1	2	5	4	4	4	2	1	2
4	3	5	5	4	1	3	1	5
3	4	4	5	3	3	3	2	4
3	2	4	5	5	1	3	1	4
2	2	3	4	3	4	3	1	4
3	2	4	3	3	2	4	4	3
1	2	5	5	4	2	2	1	4
3	3	5	5	4	3	3	1	3
3	2	4	5	4	3	4	2	3
1	5	4	2	5	1	2	3	5
1	5	5	5	5	1	1	1	4
1	5	5	5	5	1	2	1	5
1	2	4	1	2	3	1	1	2
1	4	5	4	4	3	1	1	3
2	4	4	3	4	2	2	1	4
4	3	5	2	4	2	1	1	2
2	2	4	1	4	4	3	1	4
4	2	3	3	2	1	999	999	2
2	4	5	3	4	2	1	1	3
4	2	4	4	4	2	2	3	3
1	4	4	3	4	5	2	2	4
2	2	4	3	3	2	4	1	3
2	3	4	4	3	2	3	2	999
2	3	5	4	2	2	2	1	2
4	2	5	3	3	4	5	1	2
2	2	4	3	4	3	2	2	4
5	1	2	3	2	2	1	2	2
3	2	4	1	3	2	3	2	2
4	2	5	3	3	3	3	2	3
1	4	4	2	3	1	1	1	2
2	3	4	1	3	3	2	2	5
3	5	5	4	4	4	3	3	4
2	1	4	3	2	4	5	5	3
2	4	4	1	2	3	2	4	3
4	4	5	5	3	2	1	1	5
4	3	5	1	3	2	2	1	5
3	1	5	3	5	1	5	1	5
3	2	4	5	3	2	2	2	3
4	3	4	1	4	2	2	1	4
1	4	3	4	5	2	3	4	3

3	4	4	5	3	2	3	2	3
2	4	5	5	4	3	1	2	4
2	2	4	5	4	2	1	2	3
4	3	5	2	3	2	2	4	4
2	1	5	3	4	2	2	2	4
3	4	5	1	5	2	3	3	4
1	4	5	4	4	3	2	4	5
1	1	5	4	5	1	5	1	2
4	5	5	5	5	1	1	1	5
4	2	5	1	4	2	1	1	5
3	1	3	2	3	4	2	3	2
3	2	3	4	2	2	2	1	2
2	3	4	3	2	3	4	2	2
3	3	5	3	4	3	3	1	3
2	4	4	1	4	2	1	1	2
3	2	4	2	2	2	3	1	4
3	2	3	1	2	3	4	1	3
4	3	4	2	4	3	4	2	4
3	3	4	2	3	4	2	2	3
3	3	4	3	3	2	3	3	3
2	4	4	3	5	2	1	1	4
2	3	5	3	4	1	2	3	4
3	3	4	3	4	2	3	2	4
3	2	4	1	3	3	3	2	3
5	4	4	2	2	1	5	4	4
4	1	4	3	4	3	5	3	4
5	5	4	1	2	1	4	2	4
1	3	4	3	4	4	4	1	4
2	4	5	5	5	3	2	2	4
1	2	5	1	5	5	2	1	4
2	4	5	3	4	3	1	1	4
3	3	5	4	4	3	3	2	4
2	3	5	3	3	2	2	1	4
2	3	4	1	3	3	4	1	4
2	4	5	1	4	2	3	2	4
2	3	3	4	4	2	3	1	5
3	2	5	4	1	2	4	1	3
3	3	4	4	4	2	4	2	4
5	4	5	1	5	2	2	1	4
1	4	4	5	3	3	2	1	4
1	4	4	1	5	2	2	1	5
2	4	3	4	999	2	2	1	5
3	3	4	5	2	2	3	1	5
2	1	5	3	4	3	3	1	3
2	2	3	1	3	3	3	3	3
4	3	5	3	4	1	3	1	5
5	3	4	1	4	1	2	1	2
1	2	2	5	4	2	2	1	2
2	3	5	1	4	2	3	1	5
4	5	3	2	3	4	4	2	1

4	4	4	4	3	3	2	2	4
3	2	4	1	4	1	5	1	4
4	1	5	3	4	2	5	5	4
1	5	5	4	5	1	1	1	5
5	2	5	1	3	2	5	1	4
2	4	5	5	5	2	2	1	4
1	4	5	2	999	3	3	1	5
1	4	2	3	2	2	2	1	3
4	4	1	4	4	1	1	1	5
4	4	4	3	3	2	4	1	4
3	2	4	5	3	4	4	2	4
2	4	3	4	2	2	5	1	4
3	4	4	2	3	3	1	1	4
5	4	5	5	5	3	4	1	5
4	4	4	3	3	2	4	2	3
1	2	5	5	2	5	5	5	5
3	4	4	4	4	3	3	2	3
4	4	4	5	3	3	2	1	4
2	4	5	5	5	2	5	5	5
2	3	4	4	3	3	2	1	2
3	1	5	1	3	2	5	3	4
2	4	3	2	4	2	3	1	4
3	3	5	5	3	3	4	1	3
4	2	5	3	5	1	3	1	4
2	2	4	1	3	2	4	2	3
3	3	4	3	2	3	1	2	4
999	999	999	999	999	999	999	999	999
3	4	5	1	4	5	2	1	4
4	3	4	2	4	3	3	1	3
2	2	4	1	4	2	3	1	5
2	2	5	4	4	5	2	1	4
2	3	5	1	4	3	1	4	5
2	3	4	5	4	1	2	1	3
1	2	2	1	3	2	1	2	1
2	3	4	1	4	3	3	2	3
3	2	4	3	2	3	3	3	2
1	5	5	1	5	1	999	1	5
3	2	2	1	3	3	5	4	2
2	3	5	5	4	3	5	1	4
2	3	4	999	999	3	2	1	4
2	3	4	4	4	2	4	3	4
2	3	4	4	4	2	1	1	4
2	2	5	2	4	1	4	1	5
2	2	2	3	3	4	4	3	2
3	4	2	3	4	2	3	4	3
2	4	4	4	4	4	3	1	4
1	4	5	5	5	2	2	1	5
5	4	3	5	4	3	2	3	5
3	3	2	2	2	2	3	3	3
2	2	4	5	4	4	1	1	5

3	3	5	1	4	1	1	2	4
3	2	3	1	4	2	4	2	4
3	3	4	2	3	2	1	2	5
2	3	4	3	4	3	2	2	4
1	2	5	2	2	2	2	1	4
1	5	5	1	5	1	1	1	5
1	2	4	2	1	2	3	2	2
4	4	4	5	3	4	1	2	4
3	3	4	2	3	1	3	999	3
3	3	5	3	5	1	4	1	5
1	4	5	1	4	3	2	1	5
3	3	4	5	3	2	2	1	4
3	4	5	1	3	4	1	1	2
1	4	4	3	4	2	2	2	4
3	3	5	4	3	2	3	2	5
2	4	4	5	4	5	2	1	4

BES10	BES11	BES12	BES13	BES14	BES15	BES16	BES17	BES18
5	3	5	1	5	4	5	5	1
5	3	4	3	5	1	4	3	1
4	5	4	1	5	4	5	3	1
5	3	4	1	4	4	5	5	1
4	5	3	3	5	2	5	1	1
3	5	5	2	5	2	4	4	2
999	999	999	999	999	999	999	999	999
4	5	5	1	5	5	5	5	1
3	3	1	4	2	4	5	4	1
5	5	5	1	5	4	5	5	1
3	5	1	2	5	1	5	2	1
4	1	4	2	4	3	4	3	1
999	4	2	4	4	2	3	3	3
4	999	1	3	3	4	3	2	2
3	4	3	1	5	4	5	4	1
4	3	4	3	4	3	4	2	3
3	5	3	2	4	3	4	3	1
4	3	4	2	4	3	5	3	1
2	1	2	2	3	4	5	4	3
999	999	999	999	999	999	999	999	999
5	1	4	1	5	5	999	4	1
2	3	5	4	5	1	5	3	1
3	2	4	2	5	3	5	3	1
2	5	3	3	4	4	3	3	3
2	5	4	3	4	3	5	3	1
4	5	4	3	4	3	3	2	1
4	5	3	2	3	3	3	4	1
5	5	5	2	5	3	5	4	1
2	1	2	2	4	1	5	4	1
4	5	4	2	4	4	5	5	2
5	4	5	5	5	3	5	1	1
999	5	3	3	4	3	4	3	3
3	2	3	2	3	2	3	3	2
4	4	3	1	4	2	4	2	2
4	4	3	2	5	4	3	4	2
3	3	3	5	4	3	3	3	4
3	5	4	4	4	3	4	3	1
5	5	5	5	5	4	5	1	4
4	4	4	3	4	2	4	4	3
2	5	4	2	4	1	5	4	1
999	999	999	999	999	999	999	999	999
999	999	999	999	999	999	999	999	999
4	3	4	3	4	2	4	3	4
3	5	3	2	3	1	3	2	1
999	999	999	999	999	999	999	999	999
2	5	4	2	3	4	3	3	1
4	4	3	1	5	4	3	1	4
2	4	4	3	4	2	4	3	2
3	5	3	3	999	3	4	2	1

2	5	5	4	5	3	4	5	3
5	2	2	3	5	3	4	4	1
5	1	5	4	5	2	5	3	4
4	5	4	1	4	1	5	1	2
5	5	5	1	5	5	5	1	1
5	5	5	1	5	5	5	2	5
5	4	5	1	5	4	5	3	1
5	5	5	1	5	5	5	3	3
4	3	3	4	4	3	4	3	1
2	4	4	5	4	2	4	2	2
3	5	3	1	5	1	5	4	1
2	5	2	4	5	3	4	4	1
2	5	1	1	5	1	1	1	1
4	4	3	3	4	4	4	4	2
5	4	4	2	5	4	5	2	1
3	3	3	3	4	2	4	3	1
4	2	4	2	4	3	4	3	3
3	4	4	3	4	3	4	3	1
999	999	999	999	999	999	999	999	999
999	999	999	999	999	999	999	999	999
4	4	4	2	5	3	4	1	1
4	4	5	1	5	5	5	3	1
999	999	999	999	999	999	999	999	999
3	3	3	3	3	5	3	3	3
999	999	999	999	999	999	999	999	999
999	999	999	999	999	999	999	999	999
999	999	999	999	999	999	999	999	999
3	1	3	2	2	1	4	3	2
5	5	5	1	4	4	4	4	1
5	5	5	5	5	1	5	5	5
3	4	3	2	5	2	4	4	1
2	1	3	1	5	1	5	4	1
5	3	4	1	5	3	5	4	1
3	3	1	1	999	999	3	1	1
4	4	4	3	5	2	5	4	2
5	4	5	3	5	5	5	5	1
2	4	3	4	3	4	3	1	3
3	4	3	3	4	2	999	2	1
3	4	3	3	4	2	4	2	2
2	5	4	3	4	1	4	2	5
4	5	3	2	4	3	5	4	1
4	4	5	5	5	1	3	3	4
5	5	3	2	5	4	3	5	1
999	3	3	3	4	2	3	2	1
2	1	3	2	5	1	4	1	3
3	5	3	2	4	2	4	3	2
4	4	4	1	4	3	5	3	1
3	1	2	1	3	3	3	2	2
3	5	3	1	4	3	3	2	1
4	5	3	3	4	4	4	3	1

2	4	1	5	5	1	5	4	1
4	4	4	1	4	4	3	1	1
4	5	4	1	4	4	5	3	2
4	5	4	1	5	5	5	4	1
5	4	5	3	5	3	4	3	2
5	3	5	4	5	3	5	3	4
4	4	3	4	5	2	4	2	2
5	5	5	1	5	4	5	5	1
5	5	2	2	3	3	5	3	2
5	1	5	5	5	1	5	1	1
3	4	3	3	4	2	4	2	3
4	4	4	3	4	3	4	3	1
1	1	1	1	1	1	5	5	1
1	1	1	3	1	1	1	1	3
3	4	5	3	5	3	5	4	2
5	5	4	5	5	1	4	1	2
4	4	4	1	4	3	4	4	1
3	5	1	3	5	1	3	2	1
5	5	5	1	5	5	5	999	1
1	2	3	1	3	1	5	2	2
3	4	3	1	4	2	3	2	1
4	3	3	4	999	2	3	2	2
5	4	4	2	5	4	5	4	1
4	3	4	999	4	3	4	3	3
2	999	1	2	999	3	4	3	2
4	3	3	2	4	2	4	2	1
4	3	4	2	5	2	4	2	2
4	5	4	1	5	4	4	2	1
2	2	2	2	4	3	2	3	2
4	2	3	1	2	1	3	2	3
4	4	5	1	5	5	5	4	1
3	4	4	2	5	2	5	3	1
3	4	5	5	4	2	5	2	2
2	3	2	3	2	3	2	3	3
3	4	5	3	2	4	3	2	2
2	5	3	2	3	2	3	2	3
3	5	2	3	4	2	5	2	1
4	3	4	2	4	3	5	5	1
4	4	5	1	4	3	2	4	1
3	1	3	4	3	4	4	2	2
4	3	3	4	4	4	4	3	2
5	4	5	5	5	1	5	4	1
3	4	4	4	4	3	4	3	3
4	3	3	3	4	2	4	2	1
3	5	3	3	4	2	3	2	3
3	3	3	3	4	2	4	3	2
3	4	4	3	5	4	4	5	1
1	3	4	1	4	5	5	3	1
3	5	4	2	5	4	4	3	1
4	4	2	3	3	2	4	4	1

4	4	3	3	5	4	4	3	3
4	4	3	2	4	4	3	2	1
4	2	4	1	5	1	5	2	1
2	1	1	5	5	2	5	5	1
1	3	4	2	4	4	5	3	1
4	5	4	1	5	3	5	3	1
4	3	4	2	5	2	4	2	1
5	3	5	1	5	4	5	3	5
2	3	5	2	4	2	5	1	1
5	3	3	1	3	2	4	3	2
3	5	4	2	5	3	3	2	1
1	5	4	3	5	4	4	3	1
1	5	1	2	1	5	5	1	4
3	4	3	2	3	3	3	4	2
3	2	2	4	4	1	4	3	1
4	5	3	2	4	3	4	4	1
5	3	5	4	5	3	5	3	2
4	5	4	2	5	4	999	1	1
3	3	4	1	3	3	5	3	1
4	5	4	2	4	3	4	3	2
4	5	4	3	4	3	4	4	1
5	3	5	2	5	2	5	2	1
5	3	4	2	4	4	5	4	1
3	5	4	3	5	4	5	3	2
3	4	5	1	4	2	5	3	1
3	5	2	1	4	3	3	4	1
5	3	4	2	5	2	5	3	1
4	4	3	4	5	4	5	3	1
4	5	5	2	5	4	5	3	1
4	5	4	2	4	2	4	2	1
4	4	3	2	4	4	4	5	1
3	5	2	4	5	4	5	1	1
2	1	3	2	4	1	5	4	1
4	4	3	3	4	4	4	2	2
5	4	4	1	3	999	3	4	1
2	5	2	1	3	2	2	3	1
4	2	4	5	3	2	4	2	1
2	1	5	3	999	2	4	2	3
4	3	2	3	5	3	4	999	1
3	5	5	4	5	5	4	5	5
4	5	4	4	4	4	5	4	1
3	3	4	2	4	3	5	4	1
3	4	3	2	5	3	5	3	1
4	2	4	2	4	3	5	3	2
2	3	4	3	3	3	2	1	1
1	4	2	4	5	4	5	3	1
4	4	4	2	4	4	4	4	1
3	4	2	2	3	2	3	1	2
3	4	3	2	3	2	4	3	1
4	1	4	3	4	3	5	3	2

3	4	2	3	4	3	4	4	1
1	4	5	1	5	1	5	1	1
5	4	5	1	5	4	5	4	1
5	4	5	4	5	3	5	3	1
4	5	5	2	5	4	5	3	1
3	5	4	3	5	3	5	5	1
4	3	3	2	5	2	5	3	1
5	5	5	2	5	5	5	5	1
4	4	4	1	4	3	4	3	1
3	5	1	5	4	1	1	4	1
2	2	1	5	1	1	1	1	3
5	1	3	3	5	4	5	5	1
3	5	4	4	4	5	5	3	1
4	4	4	1	5	1	5	1	2
4	5	4	1	5	5	5	1	1
4	5	4	4	2	2	4	4	1
4	5	3	5	5	3	4	5	1
4	3	4	2	4	999	2	1	3
4	4	3	2	4	3	5	3	1
1	5	2	1	1	2	3	1	2
5	4	5	5	5	1	5	1	3
4	5	3	4	4	3	4	4	1
3	4	4	2	3	2	5	2	1
3	4	3	4	4	2	999	1	2
2	4	4	5	5	3	5	4	2
4	3	4	3	4	3	4	2	1
4	1	1	5	4	1	4	3	3
3	2	3	4	3	1	4	3	1
4	1	4	1	4	2	5	2	1
5	3	4	3	4	3	5	3	3
3	3	4	2	4	4	4	3	1
2	4	4	4	5	4	4	3	2
4	2	2	2	4	3	4	3	1
5	3	3	2	4	3	3	4	3
4	4	3	3	4	4	4	3	1
5	4	5	1	5	4	5	4	1
4	5	4	2	5	3	5	3	2
4	4	3	2	4	4	4	4	2
4	4	3	3	5	1	5	3	1
4	1	2	3	4	1	4	1	3
4	3	2	4	4	4	5	3	1
5	4	4	3	4	2	3	2	1
5	5	4	1	5	4	4	3	1
4	5	4	1	5	4	5	3	1
3	2	4	3	4	3	5	1	2
4	3	5	3	4	3	4	5	1
4	4	4	4	4	4	4	4	2
5	4	4	2	4	3	5	3	1
4	5	5	2	5	3	4	4	1
999	999	999	999	999	999	999	999	999

4	5	5	3	5	1	5	4	1
4	3	3	2	4	5	5	4	2
4	5	4	3	1	4	5	4	5
3	4	3	2	3	4	4	4	2
3	2	3	2	3	3	3	3	3
5	3	4	1	5	4	5	5	1
2	2	4	3	4	1	4	4	3
4	3	4	1	5	4	5	3	1
4	5	4	2	4	4	4	4	4
3	5	3	3	3	3	4	3	3
3	1	3	2	4	2	3	4	3
3	4	3	3	4	3	4	3	1
4	5	3	2	5	2	5	1	1
5	4	4	2	5	3	5	4	2
4	5	5	2	5	3	4	2	1
4	5	3	2	4	4	5	3	1
4	5	4	1	4	4	4	3	1
3	4	4	4	5	4	4	3	1
4	5	4	4	4	2	4	2	2
2	4	2	5	4	3	5	4	5
2	4	3	5	4	4	4	2	2
4	4	3	2	4	3	4	4	2
5	3	2	2	5	2	5	4	1
4	1	4	1	4	1	4	4	1
5	4	4	5	5	4	5	3	1
4	3	4	2	4	2	4	3	1
3	1	3	5	5	3	5	5	1
5	5	5	2	3	4	3	4	1
3	5	4	1	4	4	4	3	1
4	2	5	2	4	2	4	3	1
3	4	4	2	4	5	3	999	1
2	3	2	3	2	3	2	3	2
4	4	3	3	4	3	4	2	1
4	4	5	3	4	2	5	4	2
3	4	3	3	4	3	2	2	2
4	1	3	4	4	1	4	3	1
4	4	3	2	4	3	4	4	1
1	999	3	5	1	5	5	1	4
3	5	3	3	4	2	4	4	1
3	5	5	1	4	4	4	3	1
5	5	2	3	4	3	5	4	1
4	4	4	2	4	4	4	1	1
4	5	4	1	3	4	3	2	1
2	4	3	3	4	5	4	3	2
2	4	2	4	4	4	3	3	999
4	3	3	2	4	1	4	1	1
4	2	5	3	5	5	5	4	1
4	4	4	4	4	3	5	3	2
5	5	5	4	5	3	5	2	1
3	4	3	2	3	4	4	2	2

4	4	5	3	4	4	5	4	1
3	4	3	3	4	4	5	2	1
4	3	4	2	4	4	5	5	1
4	5	4	3	4	3	5	3	2
5	4	5	999	1	3	999	2	1
2	1	3	2	1	3	2	3	3
3	5	4	999	4	5	4	5	1
5	1	4	3	4	2	4	4	1
5	3	3	3	5	4	5	4	1
5	5	5	3	5	1	5	2	1
3	2	3	4	5	3	4	3	2
5	3	4	1	5	4	5	3	1
5	5	5	2	5	3	5	3	1
4	4	5	4	5	5	5	1	1
5	5	4	2	5	4	5	4	1
5	2	4	2	5	1	5	2	1
5	3	5	3	4	2	5	3	1
3	4	4	2	4	2	4	2	1
4	3	4	2	4	3	4	3	3
5	3	4	2	4	2	4	2	1
3	5	2	3	4	3	4	3	1
3	5	4	4	5	5	5	4	1
4	4	3	3	4	3	4	3	1
2	3	3	3	3	4	3	3	2
4	3	3	3	4	3	5	2	4
4	3	4	2	4	3	4	4	2
5	5	4	1	5	2	5	5	1
3	3	2	3	3	1	3	1	2
4	2	4	3	4	3	4	3	2
4	3	3	3	4	3	5	4	2
3	1	3	2	4	3	4	3	1
3	5	3	2	4	4	3	3	1
4	4	4	3	4	3	5	4	1
4	3	4	2	4	3	3	3	3
5	1	4	2	4	4	5	4	1
3	4	4	3	4	3	4	3	1
4	4	2	2	3	3	3	4	2
3	3	5	4	3	5	2	3	4
3	3	3	3	3	2	3	3	3
5	5	5	4	5	5	5	4	1
4	5	4	3	5	3	4	3	1
4	3	4	1	4	2	4	1	1
4	4	4	2	4	3	4	4	1
4	1	3	5	5	1	4	1	1
5	4	4	2	5	5	4	4	2
4	1	4	3	4	3	5	2	2
5	3	5	2	5	2	5	2	2
4	3	2	1	4	3	5	3	1
3	5	2	3	5	1	4	2	1
4	2	2	3	4	3	5	2	2

5	4	4	4	5	1	5	1	1
4	4	4	5	5	3	5	3	1
2	4	2	3	4	2	4	1	3
5	5	2	4	999	1	2	1	2
2	4	4	3	4	2	2	3	1
5	2	4	2	5	4	4	4	2
4	4	2	1	5	1	5	1	1
4	3	4	3	4	4	5	4	1
4	4	5	2	4	3	4	3	2
4	5	999	2	5	2	5	4	1
5	4	4	5	5	1	5	1	1
3	4	3	3	3	3	2	3	3
3	2	2	2	4	3	4	2	3
4	4	2	2	4	3	4	2	2
4	1	2	4	4	2	4	3	2
4	3	3	2	5	3	5	1	1
5	3	4	5	4	4	5	4	1
3	1	3	4	5	3	5	3	1
4	5	3	1	4	3	4	3	1
4	3	3	3	4	2	4	1	2
4	5	3	4	2	3	5	3	2
5	4	3	2	4	3	5	4	1
4	4	2	2	4	4	3	3	3
4	3	4	5	5	2	4	3	1
3	5	4	1	5	4	5	4	1
3	4	3	2	4	4	4	4	1
3	4	2	2	4	2	5	2	2
4	4	3	2	4	3	999	2	1
3	5	4	3	4	3	5	3	2
2	2	1	2	4	3	3	1	3
4	2	4	1	4	3	4	3	1
999	999	999	999	999	999	999	999	999
2	4	3	2	4	3	3	4	3
4	3	3	4	4	1	5	2	2
2	3	4	3	4	2	2	3	1
3	5	3	4	3	4	3	2	1
5	4	5	2	5	4	4	4	1
4	4	4	1	3	2	4	4	1
4	3	4	2	4	3	4	5	1
4	5	4	1	4	3	5	5	2
5	3	4	2	5	2	4	2	1
4	5	5	1	4	5	4	2	1
3	5	2	1	4	4	4	4	2
4	4	4	999	4	4	5	4	1
2	1	4	1	4	2	4	1	2
4	5	4	3	5	2	5	3	1
4	5	4	2	4	3	5	4	1
4	5	3	3	4	2	3	1	2
3	4	3	3	4	4	4	3	1
3	5	3	2	5	4	5	3	2

4	4	5	3	5	5	5	2	1
4	4	4	2	4	4	3	4	1
4	5	3	2	4	2	4	4	1
3	3	3	3	3	3	3	3	3
4	2	4	3	2	2	4	2	2
4	4	5	3	5	2	4	3	1
999	999	999	999	999	999	999	999	999
3	5	4	3	4	3	3	3	2
4	4	4	1	4	4	4	4	1
5	4	3	3	4	1	4	3	1
4	5	4	1	4	5	5	3	1
3	3	4	3	5	1	5	3	2
4	3	2	2	4	4	3	3	1
4	4	4	2	4	3	4	2	2
5	5	5	2	5	2	5	3	999
4	4	4	1	5	2	4	3	1
4	1	4	5	5	1	4	2	3
4	5	4	1	5	4	4	4	1
4	5	4	3	5	3	4	4	1
3	5	3	2	5	3	3	2	1
3	4	3	3	5	2	5	2	3
2	4	4	2	5	3	4	2	1
3	4	4	3	4	3	5	4	1
4	4	4	2	4	3	4	3	4
5	4	4	2	2	999	4	2	1
4	4	4	2	4	4	3	3	3
4	4	4	2	4	2	3	4	2
4	4	3	2	4	3	4	4	1
2	4	4	1	4	2	3	3	1
4	5	3	2	5	3	4	4	1
4	3	4	3	5	1	4	1	2
5	5	4	4	5	5	4	3	1
999	999	999	999	999	999	999	999	999
4	2	3	1	4	2	4	3	1
4	4	3	2	4	2	1	3	1
4	5	3	2	4	3	5	2	2
5	4	4	1	5	4	4	4	1
4	5	3	2	3	3	3	3	1
4	4	4	3	5	3	5	3	1
3	3	4	2	4	2	5	3	1
5	5	5	2	5	2	5	4	1
2	3	4	3	4	2	4	1	2
3	2	3	5	3	3	3	1	1
5	4	2	3	4	3	1	1	1
3	3	4	2	4	2	3	3	2
3	4	4	3	4	2	4	2	2
4	2	5	3	5	1	5	3	4
3	5	4	3	4	3	4	2	3
4	4	3	2	4	3	5	3	1
5	4	5	1	5	2	5	2	1

3	4	3	2	4	2	4	2	2
4	4	5	3	5	5	5	3	2
4	2	3	3	4	2	4	2	1
4	5	4	4	5	4	5	4	1
4	5	3	1	3	3	3	3	1
3	5	4	1	4	2	5	2	1
5	4	4	1	4	3	5	1	1
3	4	4	2	4	2	3	2	3
4	4	3	2	4	4	5	4	1
3	3	4	2	3	2	4	4	2
3	5	4	3	4	4	3	5	4
2	4	3	2	2	1	3	2	4
4	4	4	2	4	3	4	4	2
4	4	3	3	4	3	4	3	2
5	4	4	3	5	4	4	1	2
3	4	3	1	4	2	5	2	4
2	2	1	4	4	2	3	1	1
999	999	999	999	999	999	999	999	999
1	4	2	3	3	4	2	4	1
5	3	4	2	1	4	4	2	3
4	5	3	3	5	4	4	5	2
2	1	2	1	3	1	4	1	3
2	2	1	2	5	1	3	2	3
3	4	4	2	4	3	4	3	1
999	3	4	3	2	1	1	999	1
3	4	3	2	5	3	5	3	1
5	3	3	2	5	3	5	3	2
999	1	2	1	1	1	5	3	1
1	1	1	5	1	1	1	2	2
4	3	2	1	4	1	5	1	1
5	1	5	1	5	1	5	1	1
2	3	2	1	3	1	3	1	2
4	2	1	2	4	1	4	1	1
3	2	4	3	5	4	3	2	3
1	5	4	3	2	1	2	3	1
3	4	3	2	3	2	3	3	3
5	5	5	1	5	5	5	2	1
4	3	4	2	4	3	4	3	1
999	999	999	999	999	999	999	999	999
999	999	999	999	999	999	999	999	999
999	999	999	999	999	999	999	999	999
4	1	4	4	5	4	5	1	3
999	999	999	999	999	999	999	999	999
2	3	1	2	1	1	1	2	3
5	4	4	1	5	4	4	4	1
3	5	5	1	4	3	5	2	1
1	1	3	1	5	1	5	2	1
4	4	4	4	4	3	4	4	2
3	4	3	1	5	5	5	1	5
5	3	5	1	5	1	5	2	1

1	4	3	4	3	2	4	3	2
3	2	4	4	5	3	4	1	3
4	3	2	4	4	1	4	1	3
4	4	3	1	5	4	4	4	1
4	2	4	4	5	2	5	2	2
999	2	3	3	2	3	2	1	2
5	5	2	1	5	1	5	2	1
5	4	4	2	4	1	5	4	1
4	4	4	1	5	2	4	3	1
2	3	4	3	5	3	4	3	2
4	3	3	4	4	3	4	3	2
4	1	4	3	5	3	5	3	2
999	999	999	999	999	999	999	999	999
999	999	999	999	999	999	999	999	999
4	4	4	2	3	3	4	4	4
5	1	4	1	4	4	4	3	5
4	3	5	1	5	1	5	2	1
3	3	4	5	4	4	4	3	4
5	4	4	2	5	1	5	4	1
3	3	4	2	4	2	5	3	2
4	1	2	3	5	1	5	2	1
999	999	999	999	999	999	999	999	999
4	1	4	3	4	1	4	2	1
4	4	3	2	4	2	3	4	3
4	1	3	3	5	1	4	2	1
5	5	5	1	5	4	5	999	1
3	1	3	5	3	2	5	3	1
5	4	3	1	5	1	5	1	1
5	5	5	1	5	4	5	1	1
4	4	5	1	5	2	4	4	2
4	4	4	3	4	3	5	3	2
5	1	5	1	5	1	5	3	5
3	5	4	3	5	2	5	3	4
3	2	2	2	3	2	4	3	2
4	5	3	5	5	4	5	1	3
3	2	2	3	3	2	2	3	3
4	4	3	4	5	1	3	1	1
4	2	2	3	4	1	4	1	3
3	2	1	3	2	2	2	1	1
2	999	1	1	2	1	4	1	4
5	1	1	1	5	1	5	1	1
2	2	2	4	4	2	3	1	2
3	2	3	2	4	3	4	2	3
3	5	3	3	5	1	3	3	1
3	3	3	4	4	2	4	3	2
4	2	3	3	4	2	3	1	3
4	2	2	2	4	2	4	2	2
3	2	3	2	2	3	3	3	2
5	3	3	3	4	2	4	3	2
4	1	4	5	3	1	4	3	2

4	2	1	1	4	1	3	2	2
3	3	2	3	4	4	5	4	2
4	1	2	4	4	1	4	2	2
3	4	5	1	5	1	5	1	1
3	3	2	3	2	3	2	3	2
3	2	4	4	4	2	4	3	1
3	3	3	3	3	3	3	3	3
4	3	2	3	4	2	4	4	2
3	4	4	2	4	3	4	999	2
4	5	4	1	5	5	4	5	1
3	2	3	2	4	2	4	3	2
3	1	3	4	5	2	4	2	3
3	4	2	3	3	2	2	3	3
3	5	1	5	4	3	2	4	3
1	1	3	4	3	2	5	3	1
5	5	5	5	5	5	5	2	1
1	3	2	2	5	2	5	1	3
3	4	3	3	4	3	4	4	2
3	3	4	3	5	3	4	2	1
3	4	4	4	4	2	3	3	2
3	3	2	4	5	1	4	2	3
3	3	2	3	3	3	3	1	2
3	4	3	4	3	3	3	3	2
5	1	2	4	4	2	5	1	3
5	4	4	2	4	2	5	3	1
3	3	3	3	3	3	3	3	3
4	4	4	1	4	2	4	2	1
5	5	2	3	2	1	2	1	1
4	4	3	2	4	3	4	5	1
5	1	4	1	5	3	4	3	1
5	5	5	1	5	3	5	3	1
3	4	3	1	4	4	4	3	1
2	3	4	2	4	3	4	3	1
4	2	3	2	4	4	4	4	1
3	3	3	4	4	3	2	2	3
4	4	3	2	4	2	4	4	1
3	2	1	5	4	3	3	2	2
5	4	4	2	4	3	4	2	2
2	2	2	2	2	2	2	2	2
4	3	3	3	3	2	2	2	2
3	1	1	1	4	1	2	1	3
5	2	3	4	4	2	4	2	2
3	4	4	2	3	3	4	3	1
4	3	4	3	4	3	4	2	1
4	2	4	2	4	3	4	4	2
3	5	2	2	2	3	2	3	1
3	1	3	2	4	2	5	3	3
5	5	2	5	4	2	5	1	5
4	4	2	1	4	4	4	4	1
3	4	3	2	4	1	4	2	4

2	5	5	2	2	3	4	2	2
4	2	4	5	4	1	5	3	1
4	3	4	2	5	2	4	3	1
3	2	3	2	5	2	3	2	2
3	3	3	3	3	3	3	3	3
5	5	5	5	999	1	5	1	1
2	3	2	4	2	4	2	3	4
4	2	1	1	5	5	5	1	5
4	4	4	3	4	3	5	3	2
5	2	5	2	5	3	5	4	1
3	1	3	2	4	1	5	1	3
4	1	4	2	4	4	4	3	1
3	4	3	3	4	3	3	2	4
3	1	1	2	2	2	1	1	4
5	5	5	1	5	1	5	5	1
4	3	4	3	4	1	5	3	999
3	4	3	3	4	2	3	4	3
4	4	4	2	4	3	4	3	2
3	4	5	5	5	3	4	4	1
3	3	3	3	3	3	3	4	1
3	4	1	4	2	1	5	2	1
3	999	4	2	4	2	3	2	3
1	1	1	1	2	2	2	1	2
4	3	4	1	5	3	5	1	3
4	5	3	2	5	5	4	3	1
3	3	3	2	3	2	4	1	2
2	1	2	2	4	3	4	2	1
4	4	3	1	4	4	4	3	1
4	4	3	2	4	3	5	3	1
4	4	3	4	4	3	4	4	2
5	4	5	1	5	3	5	4	1
3	4	5	3	5	1	3	3	2
2	3	2	4	4	2	2	2	2
2	5	4	1	3	1	4	4	1
5	5	5	1	5	5	5	5	1
4	5	3	3	4	4	4	4	1
4	4	4	1	5	4	5	4	1
3	3	4	4	5	2	5	3	1
1	1	1	3	5	1	2	1	4
2	4	1	3	1	2	3	2	4
3	4	2	5	4	4	3	3	2
4	4	4	1	5	2	5	2	1
3	4	4	1	4	2	4	4	1
3	3	4	3	5	1	5	3	3
3	3	3	3	3	3	3	3	3
2	3	4	3	5	3	2	3	4
4	5	5	2	4	4	5	4	1
4	1	2	2	3	1	4	5	2
4	3	2	4	4	2	3	2	1
3	1	1	2	5	3	5	2	1

3	4	4	4	4	3	3	3	2
2	3	2	3	3	2	3	2	1
3	2	3	3	4	2	3	3	2
4	3	3	1	3	2	3	3	1
3	2	4	2	4	1	4	1	3
3	3	3	3	3	3	3	3	3
4	4	2	1	2	1	4	1	1
3	5	3	2	3	4	3	3	2
3	2	4	2	5	3	5	2	1
3	3	3	3	3	2	2	1	2
2	2	1	2	2	2	2	3	2
4	2	5	4	5	3	5	2	1
3	4	3	3	4	3	4	2	3
5	4	5	3	5	2	5	3	1
4	4	3	2	4	4	4	2	1
3	4	3	4	4	2	3	4	2
4	4	4	5	5	2	5	2	1
3	2	3	3	3	2	3	3	2
3	2	3	4	4	3	4	3	3
5	4	5	3	4	1	5	4	1
1	5	4	1	4	1	5	4	1
5	4	5	3	5	2	5	4	2
5	4	3	2	5	1	5	3	1
4	5	3	5	5	3	4	4	1
4	1	3	2	2	1	2	2	1
3	1	3	1	4	2	5	2	1
4	4	3	2	4	2	4	3	3
4	3	2	4	3	999	2	3	4
3	2	3	1	3	2	4	3	2
2	2	3	2	4	2	3	4	2
4	3	1	2	5	3	4	3	1
3	1	1	2	3	3	2	3	2
4	1	3	4	4	2	4	3	2
4	3	1	2	3	4	5	3	1
3	4	2	3	4	1	4	4	1
4	4	3	3	4	2	3	3	3
3	4	2	5	4	2	1	2	3
2	3	4	3	3	3	3	2	1
4	3	2	3	3	3	3	3	1
3	4	3	3	5	2	4	3	2
4	3	3	3	3	3	2	3	2
4	4	3	3	3	3	3	4	4
1	1	2	2	3	5	999	5	3
2	2	2	2	4	2	3	2	3
4	3	3	2	4	2	4	4	2
4	4	3	1	5	4	5	2	5
3	4	4	2	5	2	5	2	3
5	2	3	3	4	2	3	2	3
5	1	3	5	4	1	5	2	3
3	4	5	4	4	4	3	2	3

4	3	4	3	5	3	5	3	2
3	4	2	1	5	3	4	3	2
2	1	4	1	3	5	5	5	1
4	3	2	5	3	1	4	2	5
5	2	5	3	5	2	5	3	1
5	3	4	2	5	1	4	3	2
4	4	3	3	4	3	4	4	1
5	3	4	3	4	2	4	3	1
5	1	5	1	5	3	5	2	1
3	4	1	5	2	1	1	4	1
3	2	4	3	3	3	2	3	4
2	2	2	3	3	1	3	3	4
2	3	3	3	3	2	3	2	3
3	4	999	3	3	3	999	3	2
4	3	4	2	4	2	3	4	2
3	2	4	2	3	2	4	2	2
2	4	2	3	4	2	2	3	4
4	1	4	2	4	1	4	3	2
3	3	3	3	3	4	3	3	3
2	3	3	3	4	3	3	3	3
2	3	5	2	5	2	4	4	1
4	1	2	4	4	2	4	3	2
4	2	4	3	4	2	4	3	3
3	3	3	1	4	3	5	4	3
4	1	3	3	4	1	4	3	4
4	1	4	5	4	1	4	1	3
1	1	4	4	4	4	999	2	1
4	3	3	3	3	2	4	4	2
4	3	4	1	5	4	4	5	1
4	3	4	2	5	4	5	3	2
2	5	3	4	5	1	5	3	2
3	3	3	4	5	2	5	3	1
3	1	3	3	4	2	4	1	2
3	3	3	4	4	1	3	3	2
4	1	4	2	4	3	4	2	2
999	4	4	4	5	1	4	2	2
2	1	2	3	4	3	4	1	2
4	2	3	2	4	3	4	4	2
4	5	5	5	2	1	5	3	2
4	2	3	1	3	1	4	3	3
2	2	2	4	5	1	2	2	3
3	5	4	1	3	4	3	2	3
4	3	2	3	4	1	2	3	2
3	3	4	4	4	3	3	2	1
4	2	3	3	3	2	4	3	3
5	3	3	3	4	3	4	1	3
4	3	3	5	4	1	5	1	3
2	3	3	1	4	2	2	1	1
4	1	3	4	4	1	5	2	2
2	4	3	4	3	4	5	4	1

4	2	2	4	3	4	4	2	2
4	4	3	5	5	1	4	2	3
3	1	4	5	4	1	4	3	4
5	4	5	1	5	5	5	4	1
4	3	3	4	4	2	5	1	4
4	4	4	3	4	3	5	4	1
3	2	4	4	4	2	4	2	1
3	2	2	5	5	1	3	1	1
4	1	4	2	4	4	4	4	2
4	4	2	3	4	3	4	1	3
3	4	3	4	4	2	4	4	4
2	4	2	2	3	2	2	3	2
2	1	2	1	3	1	5	2	2
5	5	5	5	5	1	5	3	4
3	2	4	3	4	2	4	4	2
5	1	3	5	5	1	5	3	1
3	3	3	3	4	2	4	4	2
4	5	4	2	4	3	3	2	4
4	3	3	4	4	4	4	3	5
3	4	3	3	3	2	3	3	2
2	3	4	1	4	1	4	4	3
2	3	3	3	4	4	4	3	2
4	3	1	4	4	4	4	3	3
4	2	4	4	4	3	5	4	2
1	3	1	3	4	2	2	3	3
3	4	3	1	4	1	4	2	1
999	999	999	999	999	999	999	999	999
4	3	5	2	4	1	4	4	1
2	2	2	3	3	3	2	3	2
4	4	4	2	4	3	4	1	2
4	5	4	2	5	3	4	1	3
4	2	4	1	5	1	4	2	3
4	2	3	2	4	4	4	3	2
3	1	3	1	5	3	2	2	1
3	2	3	4	4	2	3	4	1
4	4	3	1	5	3	2	4	3
5	5	4	1	5	2	4	4	1
2	1	3	3	3	3	2	4	3
4	3	4	4	4	3	4	3	3
4	3	4	2	5	4	3	4	1
4	3	4	3	3	3	4	3	2
1	4	3	2	3	1	3	4	3
5	4	4	2	4	2	999	3	2
3	4	3	3	4	2	3	2	3
2	3	3	3	4	3	3	4	3
4	3	3	3	4	2	4	4	2
5	3	5	1	5	1	3	1	5
4	3	2	1	2	4	3	2	3
3	4	3	1	5	4	4	3	2
4	2	4	2	4	2	4	3	1

1	1	3	5	4	2	3	2	1
4	1	4	4	4	2	4	2	2
3	2	3	3	4	2	4	2	2
4	4	4	2	4	3	4	3	2
4	1	4	1	4	4	4	4	2
4	2	5	2	5	1	5	3	1
3	2	1	2	1	2	3	2	2
3	5	4	2	4	4	4	3	2
4	3	2	3	999	1	5	3	4
5	1	5	5	5	1	4	1	1
3	1	3	5	2	1	2	3	2
3	5	3	4	2	2	3	2	2
3	2	4	4	5	5	5	3	1
2	4	4	4	4	1	4	3	2
4	2	3	3	4	3	5	4	2
4	4	4	2	4	4	4	4	1

BES19	BES20
1	1
1	1
1	1
1	2
4	3
1	1
999	999
1	1
4	3
1	1
1	2
1	2
3	2
1	1
1	1
2	2
3	1
2	2
3	2
999	999
1	1
2	1
1	1
2	3
1	1
1	1
3	2
1	1
1	2
1	1
5	1
3	2
3	2
3	3
3	2
4	4
2	2
1	1
2	2
2	1
999	999
999	999
3	2
1	1
999	999
1	2
5	1
1	2
1	1

1	1
1	1
1	1
1	1
1	1
1	1
5	5
1	1
2	1
2	4
1	1
1	1
1	2
2	1
1	1
2	2
2	2
2	2
999	999
999	999
1	1
1	1
999	999
1	1
999	999
999	999
999	999
4	3
1	1
5	5
3	3
2	1
1	1
1	1
1	1
1	1
2	3
2	1
5	2
1	1
3	3
3	2
1	2
1	1
2	1
2	3
1	1
2	2
2	5
3	2

1	1
1	2
1	3
2	1
1	1
3	1
2	2
1	1
4	3
1	1
2	2
1	1
1	5
5	5
1	1
2	1
3	2
1	1
1	1
1	2
1	1
2	2
1	1
3	3
1	3
1	1
2	2
1	1
3	3
4	5
2	1
1	1
3	2
2	3
2	2
2	3
1	1
4	1
2	2
4	2
2	2
1	1
1	1
2	1
3	4
4	3
1	4
1	1
1	1
1	1

2	2
2	2
1	1
1	1
1	1
1	1
1	1
2	2
5	4
1	1
1	1
1	1
3	5
2	2
1	1
1	1
1	1
1	1
1	1
2	2
2	2
1	1
1	1
1	1
1	1
2	3
1	1
1	1
2	1
2	1
1	1
1	1
2	3
3	3
3	2
1	2
1	4
2	1
1	1
4	3
2	2
1	4
1	1
2	2
1	3
1	1
5	1
2	3
1	2
1	1

2	2
1	1
1	2
1	1
1	1
1	2
1	1
1	1
2	2
1	4
2	4
1	4
2	2
1	1
1	1
2	2
1	1
4	4
1	1
1	2
3	1
1	1
1	1
2	3
1	3
4	1
4	1
1	1
1	1
2	3
2	2
2	1
2	2
2	1
1	1
1	1
999	1
2	1
1	1
1	4
2	1
2	2
1	1
2	1
3	2
2	4
1	1
1	1
1	1
999	999

1	1
2	3
2	2
2	3
3	3
1	1
2	2
1	1
4	4
2	2
4	1
1	1
1	1
1	3
1	1
1	1
1	2
1	1
2	2
2	3
3	2
1	1
1	1
1	4
1	1
1	1
1	3
1	1
4	2
1	1
1	1
3	2
2	1
2	2
2	3
3	2
3	2
4	1
1	1
1	2
1	1
1	2
3	1
4	2
2	1
3	4
1	1
2	1
1	1
3	3

1	1
2	3
2	1
2	1
1	1
2	1
1	1
1	1
2	1
1	1
2	1
2	1
1	1
1	1
1	1
1	1
5	1
3	2
2	1
1	2
3	3
1	1
1	1
999	3
5	1
2	3
1	1
2	2
1	1
1	1
4	2
3	2
1	1
4	1
1	1
1	1
2	2
3	2
2	2
1	1
1	1
1	1
1	2
3	1
1	1
1	1
1	1
1	1
3	2
1	1

1	1
1	1
3	2
2	1
1	1
2	1
5	2
1	1
2	3
1	1
1	1
2	2
2	3
2	1
1	1
1	2
4	1
4	1
1	1
2	2
2	2
1	2
3	2
1	1
1	1
2	2
1	1
2	3
2	2
3	5
1	1
999	999
2	2
2	1
1	1
4	2
1	2
3	2
1	2
2	2
1	1
3	2
2	2
1	1
3	3
1	1
2	1
4	4
1	1
2	3

4	1
2	2
1	1
3	3
2	2
2	2
999	999
1	1
1	1
4	2
1	1
1	1
2	2
1	1
1	1
1	2
1	2
4	1
1	1
2	2
3	3
1	2
1	1
3	4
1	1
3	3
2	1
2	2
4	1
2	1
2	3
5	5
999	999
1	1
1	1
2	2
4	1
1	2
4	1
1	1
1	1
1	1
3	3
3	2
2	2
2	2
1	1
1	2
1	1
1	1

1	1
2	1
2	2
1	1
1	1
1	1
1	1
1	1
4	1
3	3
5	4
3	2
2	2
3	3
4	2
3	5
1	1
999	999
3	4
4	1
4	4
3	1
3	1
3	1
1	1
1	1
2	1
2	1
1	3
1	1
1	1
3	3
2	2
2	3
5	5
2	2
1	1
1	4
999	999
999	999
999	999
4	3
999	999
3	5
1	1
1	4
1	1
3	2
1	3
1	1

2	2
3	2
1	3
1	1
3	2
2	2
5	1
2	1
2	1
3	1
4	1
1	1
999	999
999	999
3	4
5	1
1	1
2	2
1	1
1	1
1	1
999	999
5	1
999	999
3	1
1	1
2	3
1	1
1	1
2	1
1	1
5	1
1	3
3	3
1	4
3	4
1	2
2	1
1	1
3	3
1	1
3	3
3	2
3	1
2	2
3	3
2	4
3	2
3	4
3	4

1	1
2	3
3	4
1	1
3	3
1	2
3	3
2	1
2	2
1	1
3	3
2	3
3	4
2	2
5	1
1	1
5	2
2	1
2	2
2	2
5	3
3	5
3	2
2	4
1	1
3	3
4	1
1	1
1	4
3	3
1	2
2	3
2	2
1	1
2	2
2	2
3	2
2	2
2	2
2	3
2	2
2	1
2	3
1	1
2	3
3	3
2	3
2	5
4	3
1	1

2	2
1	1
2	3
1	2
3	3
5	999
2	4
1	1
1	1
1	1
2	2
1	1
3	3
3	3
1	1
1	2
2	1
2	2
4	3
1	1
1	1
3	3
1	3
1	1
2	1
3	2
3	2
4	2
2	1
3	2
1	1
3	3
3	2
1	2
1	1
1	1
1	1
2	2
2	1
5	3
3	4
3	2
2	1
3	3
3	3
2	3
1	1
2	3
3	999
2	2

3	2
3	2
3	2
1	1
2	3
3	3
4	4
3	3
1	2
1	1
2	1
1	1
3	2
3	1
2	4
3	2
2	1
2	1
2	2
2	1
1	1
1	1
1	1
1	1
1	2
1	1
1	1
3	2
1	2
4	2
1	1
4	2
2	2
2	3
2	2
3	2
2	1
3	3
3	3
2	1
2	3
2	1
2	1
2	5
2	2
2	1
2	1
2	3
2	2
4	4

1	2
3	2
2	5
3	1
1	1
2	1
4	2
1	1
1	1
4	2
2	3
2	2
3	3
3	4
2	2
3	3
3	4
2	4
2	4
3	3
1	5
1	1
2	2
4	3
1	3
2	1
3	1
1	1
2	1
1	1
3	1
2	2
2	1
2	1
2	1
1	1
3	2
2	2
1	1
4	3
1	1
4	1
3	4
1	2
3	3
1	1
3	3
1	1
5	1
1	2

2	2
1	1
2	2
1	1
1	2
2	1
2	2
1	1
2	3
2	2
2	2
1	2
2	2
1	1
3	4
1	1
3	2
4	3
2	2
4	3
2	2
1	2
2	1
1	1
2	2
3	3
999	999
1	1
2	3
2	2
3	3
1	4
2	1
2	3
2	3
2	4
1	1
4	1
2	2
2	2
4	3
4	2
4	4
4	4
3	3
2	2
1	1
4	3
2	3
2	2

1	1
2	2
2	2
4	2
2	1
5	1
2	3
2	3
3	3
2	1
3	3
2	3
1	1
3	3
2	1
1	1
